# Total synthesis of feglymycin based on a linear/convergent hybrid approach using micro-flow amide bond formation

**DOI:** 10.1038/ncomms13491

**Published:** 2016-11-28

**Authors:** Shinichiro Fuse, Yuto Mifune, Hiroyuki Nakamura, Hiroshi Tanaka

**Affiliations:** 1 Laboratory for Chemistry and Life Science, Institute of Innovative Research, Tokyo Institute of Technology, 4259 Nagatsuta-cho, Midori-ku, Yokohama 226-8503, Japan; 2 Department of Chemical Science and Engineering, School of Material and Chemical Technology, Tokyo Institute of Technology, 2-12-1 Ookayama, Meguro, Tokyo 152-8552, Japan

## Abstract

Feglymycin is a naturally occurring, anti-HIV and antimicrobial 13-mer peptide that includes highly racemizable 3,5-dihydroxyphenylglycines (Dpgs). Here we describe the total synthesis of feglymycin based on a linear/convergent hybrid approach. Our originally developed micro-flow amide bond formation enabled highly racemizable peptide chain elongation based on a linear approach that was previously considered impossible. Our developed approach will enable the practical preparation of biologically active oligopeptides that contain highly racemizable amino acids, which are attractive drug candidates.

## Abstract (web summary)

Feglymycin is a biologically active peptide but a challenging synthetic target due to the highly racemizable nature of the 3,5-dihydroxyphenylglycine groups. Here the authors report the synthesis of feglymycin using a microflow system, allowing amide bond formation without severe racemization.

## Graphical abstract

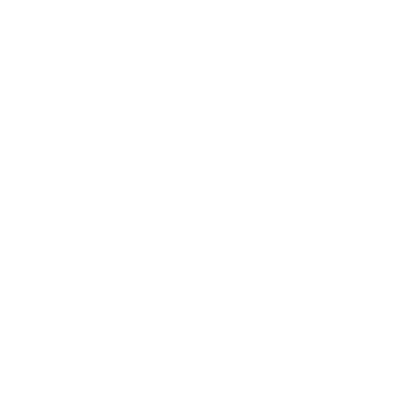

Biologically active peptides have garnered considerable attention as drug candidates because their target specificity is generally higher than that of small molecule-based drugs, and their production cost is lower than that of protein-based drugs^1^,^2^,^3^. In particular, oligopeptides consisting of <20 amino acids account for three-fourths of existing marketed peptide drugs^3^. Therefore, efficient and practical synthesis of these oligopeptides is highly important. In general, a linear/convergent hybrid approach is used for the synthesis of these oligopeptides: (1) short peptides (<10 amino acids) are prepared based on a linear strategy wherein peptide chains are elongated one-by-one. This linear strategy enables the facile installation of various amino acids for rapid analogue synthesis, and it minimizes the protecting group manipulation of terminal carboxylic acids while avoiding undesired diketopiperazine formation; and (2) the prepared short peptides are coupled in a convergent manner to afford the target oligopeptides because longer peptides are often poorly soluble against solvents. In addition, this approach facilitates purification because the molecular weight of the desired oligopeptides is much different from that of their precursors.

Many arylglycine-containing biologically active oligopeptides have been reported, and they are highly important as drugs and drug candidates. Phenylglycine (Phg), 4-hydroxyphenylglycine (Hpg) and 3,5-dihydroxyphenylglycine (Dpg) are the most important representatives of the arylglycines because they can be found in various biologically active natural products such as formadicin, ramoplanin, vancomycin and teicoplanin^4^. These arylglycines significantly contribute to the biological activity of these natural products^4^.

Despite their importance, synthetic methodologies that can be used for arylglycines-containg oligopeptides is very limited due to their racemization-prone nature. Reportedly, phenylglycine is 60 times more prone to racemization than alanine^5^. Dpg is even more racemization-prone than the phenylglycine^4^.

Feglymycin isolated from *Streptomyces* sp. DSM 11171 (refs 6, 7) is a biologically active oligopeptide composed of 13 amino acids involving Hpgs and highly racemizable Dpgs. The biological activity of feglymycin involves strong anti-HIV activity^8^ and moderate antimicrobial activity^9^,^10^, and its unique helical conformation^11^ makes it an attractive lead compound for drug development. In 2009, the Süssmuth group reported the first total synthesis of feglymycin based on a highly convergent approach, in which D-Dpgs were coupled with the neighbouring C-terminal side of amino acids at an initial stage^12^. This amidation of the D-Dpgs with amino acids was performed using a coupling agent, 3-(diethyloxyphosphoryloxy)-1,2,3-benzotriazin-4(3*H*)-one (DEPBT). Then, the resultant dipeptides/tripeptides were coupled to afford hexapeptide and heptapeptide. The DEPBT was crucial to avoid an undesired racemization reaction. A linear approach to the syntheses of the hexapeptide and the heptapeptide would have previously described merits. However, the Süssmuth group reported that the coupling of D-Dpgs with longer peptides based on a linear approach resulted in severe racemization of the D-Dpgs and provided an inseparable mixture of diastereomers even when using DEPBT. The Süssmuth group concluded that a linear approach for the hexapeptide and the heptapeptide containing D-Dpgs was not possible^12^. The development of peptide chain elongation based on a linear approach is important not only for the synthesis of feglymycin and related compounds, but also for the synthesis of other arylglycines-containg biologically active oligopeptides.

Recent marked progress in micro-flow technology^13^,^14^,^15^,^16^,^17^,^18^,^19^,^20^ has enabled precise control of the reaction time (<1 s) and temperature^21^,^22^,^23^. We have studied micro-flow acylations^24^,^25^ and recently reported micro-flow amide bond formation using a high atom economy and an inexpensive coupling agent, triphosgene^26^. We envisaged that if micro-flow amide bond formation enables the amidation of Dpgs without severe racemization, then it should be possible to use a linear synthetic approach to prepare the hexapeptide and the hepapeptide.

Herein, we wish to report the total synthesis of feglymycin based on a linear/convergent hybrid synthetic approach using a micro-flow amide bond formation that suppresses the undesired racemization. In detail, we planned to couple the hexapeptide and the heptapeptide in a convergent manner for the total synthesis of feglymycin and synthesize both oligo peptides based on a linear approach using micro-flow amide bond formation. The challenge was to suppress any undesired racemization of the highly racemizable Dpgs using our micro-flow amidation.

## Results

### Examination of micro-flow amide bond formation of arylglycines

**Figure 1:**
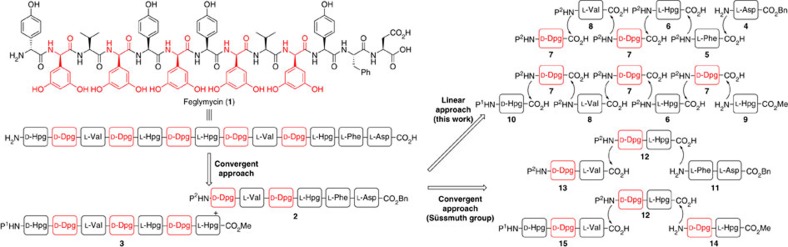
Synthetic strategy for feglymycin (1). Our linear synthetic approach and Süssmuth's convergent synthetic approaches. Süssmuth concluded that the linear approach for oligopeptides **2** and **3** containing D-Dpgs was not possible.

The key micro-flow amide bond formation of arylglycines was examined using readily available D-Hpgs **10** and **16** (Table 1). We connected two T-shaped mixers with Teflon tubing and immersed them in a water bath. A solution of carboxylate **10** in solvent A was introduced into the first mixer with a syringe pump. The solution of triphosgene in MeCN was also introduced into the first mixer with a syringe pump to rapidly generate symmetric anhydride *in situ*^26^ (<0.5 s). To accomplish amidation (<4.3 s), a solution of amine **16** in solvent B was then introduced into the second mixer with a syringe pump. The reaction was quenched by pouring the mixture into a saturated aqueous solution of NH_4_Cl, brine and ethyl acetate (micro-flow reactor setup, see Supplementary Methods). To investigate the influence of the protecting group on a nitrogen atom (=P), three protecting groups were examined (Table 1): *t-*butoxycarbonyl (Boc), **10a**; benzyloxycarbonyl (Cbz), **10b**; and, allyloxycarbonyl (Alloc), **10c**. The 9-fluorenylmethoxycarbonyl (Fmoc) group was not examined because we feared that the basic deprotection conditions might cause undesired racemization. We did not protect the phenolic hydroxyl group (p*K*_a_=*ca*. 10) in D-Hpgs **10** because we did not expect the carboxylate generated from an equimolar amount of *N,N*-diisopropylethylamine (DIEA) and D-Hpg (p*K*_a_ of CO_2_H in D-Hpg=*ca.* 2) to deprotonate the phenolic hydroxyl group in D-Hpg to afford an undesired highly nucleophilic phenoxide. On the other hand, the nucleophilicity of neutral phenolic hydroxyl group is much lower than that of an amino group, and, therefore, we speculated that the undesired nucleophilic attack of the phenolic hydroxyl group would not occur. In our previous report, *N,N*-dimethylformamide (DMF) and MeCN were used for solvents A and B, respectively^26^. However, carboxylate **10a** and amine **16** were not soluble against these solvents. Therefore, *N,N*-dimethylacetoamide (DMA) was used instead of DMF and MeCN (entries 1–5). Carboxylates **10b** (P=Cbz) and **10c** (P=Alloc) afforded the desired products **18b** and **18c** in good yields (entries 2 and 3), respectively, whereas **10a** (P=Boc) afforded **18a** in a low yield (entry 1). To suppress racemization, the reaction was carried out at 10 °C. As expected, the generation of undesired epimers **19b** and **19c** was suppressed (entries 4 and 5), although a slight decrease in yield was observed in the case of carboxylate **10b** (entry 2 versus 4). The use of H_2_O/MeCN for solvent B improved the yield of **18b** in the case of carboxylate **10b** (P=Cbz) without increasing racemization (entry 4 versus 6). On the other hand, in the case of **10c** (P=Alloc), comparable results were observed (entry 5 versus 7). The corresponding batch reaction resulted in increased racemization (entry 7 versus 8). The use of *N*-methylpyrrolidone (NMP) and *N*,*N′*-dimethylpropyleneurea (DMPU) that could dissolve **10c** resulted in a decrease in the yields (entries 9 and 10). Entries 5 (**condition A**) and 7 (**condition B**) were used for the coupling of arylglycines retaining an Alloc group, and entry 6 (condition C) was used for the coupling of arylglycine retaining a Cbz group in the following peptide chain elongations. We decided to use the Cbz group for P^1^ and the Alloc group for P^2^ (Fig. 1) because the Cbz group could be removed along with the benzyl (Bn) group under hydrogenolysis conditions.

### Synthesis of C-terminal hexapeptide 2

**Figure 2:**
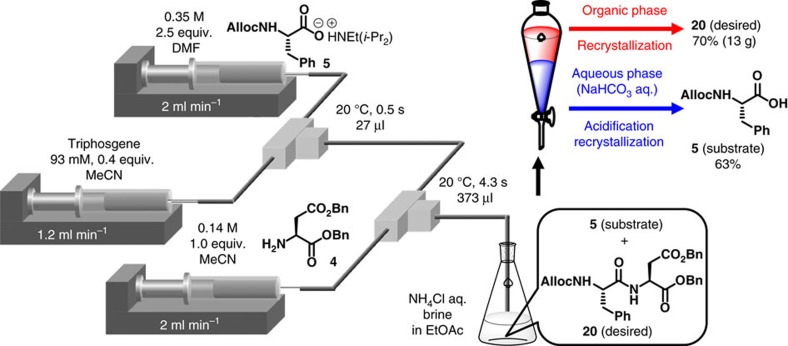
Micro-flow synthesis of dipeptide 20. Dipeptide **20** was prepared by micro-flow amide bond formation. The obtained dipeptide was readily purified via simple aqueous workup and recrystallization.

We started with a synthesis of the C-terminal dipeptide **20** (Fig. 2). Micro-flow amide bond formation afforded a mixture of **5**, **20** and the HCl salt of DIEA. The desired dipeptide **20** was readily isolated via simple aqueous workup and recrystallization (70% yield, 13 g). Salt-free carboxylic acid **5** was also recovered in a pure form (63% based on unreacted **5**) via simple aqueous workup and recrystallization.

**Figure 3:**
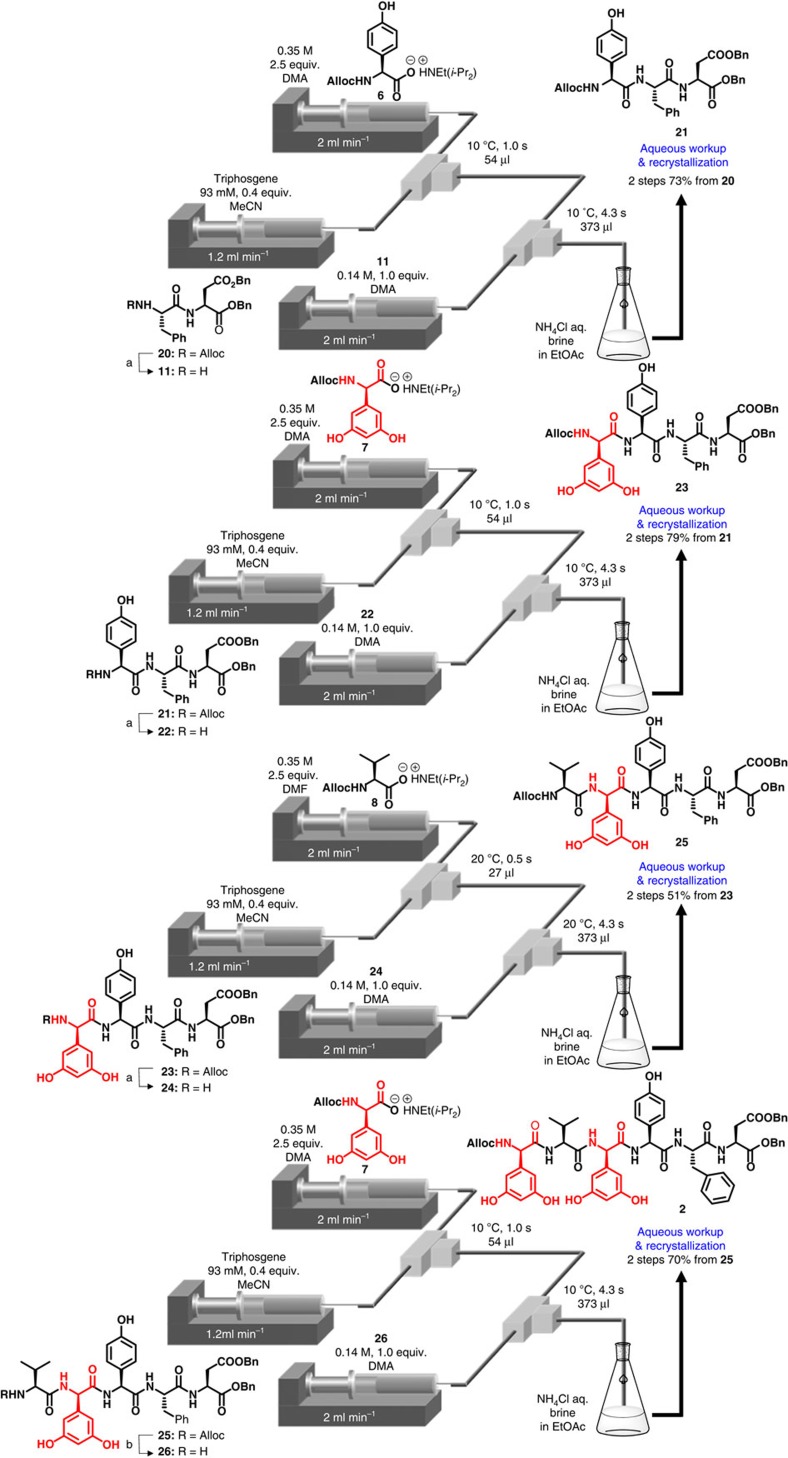
Synthesis of C-terminal hexapeptide 2 using micro-flow amidation. Conditions: (**a**) Pd(PPh_3_)_4_, PhSiH_3_, CH_2_Cl_2_/MeOH, r.t., 1–1.5 h, batch. (**b**) PS-Ph_3_P-Pd, PhSiH_3_, CH_2_Cl_2_/MeOH/H_2_O, r.t., 1 h, batch.

Peptide chain elongation was performed based on the linear approach, as shown in Fig. 3. First, an Alloc group was removed from the protected dipeptide **20** under neutral conditions^27^,^28^,^29^ using a batch reactor. Micro-flow amidation with carboxylate **6** was performed under **condition A** with slight modification (activation time: 1.0 s), because dipeptide **11** was not dissolved in the H_2_O/MeCN mixed solvent with the use of **condition B**. After recrystallization, the desired pure tripeptide **21**, which did not contain an epimer, was obtained in a 73% yield (two steps). To our delight, a coupling of **22** with highly racemizable D-Dpg **7** (refs 30, 31) afforded the desired pure tetrapeptide **23**, with no epimer, in a 79% yield (two steps) under **condition A**. The coupling of sterically hindered carboxylate **8** and tetrapeptide **24** afforded the desired pure pentapeptide **25** in a 51% yield (two steps). The removal of the Alloc group of protected pentapeptide **25** was performed using a solid-immobilized Pd catalyst in a batch reactor to facilitate the purification of polar pentapeptide. The subsequent coupling of highly racemizable D-Dpg **7** with pentapeptide **26** afforded the desired pure hexapeptide **2** with no epimer in a 70% yield (two steps).

### Synthesis of N-terminal heptapeptide 3

**Figure 4:**
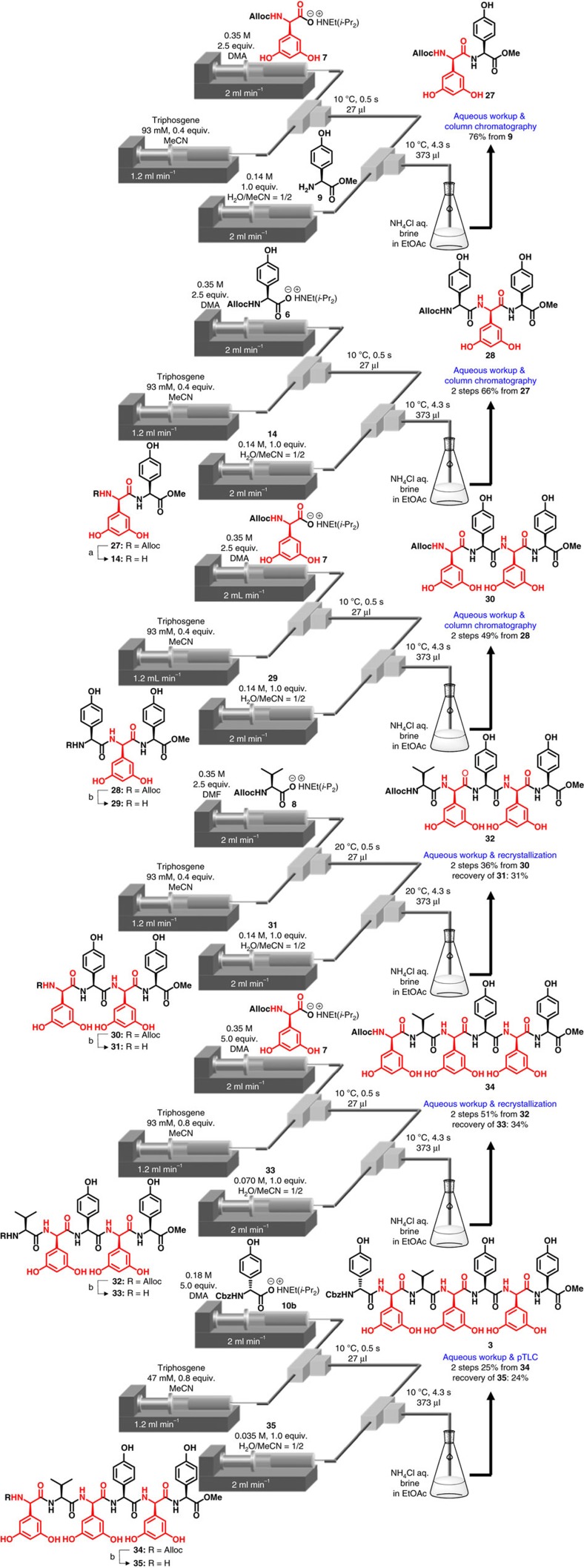
Synthesis of N-terminal heptapeptide 3 using micro-flow amidation. Conditions: (**a**) Pd(PPh_3_)_4_, PhSiH_3_, CH_2_Cl_2_/MeOH/H_2_O, r.t., 40 min, batch. (**b**) PS-Ph_3_P-Pd, PhSiH_3_, CH_2_Cl_2_/MeOH/H_2_O, r.t., 1.5–2.5 h, batch.

Synthesis of N-terminal heptapeptide **3** was performed based on the linear approach, as shown in Fig. 4. The coupling of **9** with highly racemizable D-Dpg **7** under **condition A** caused a substantial generation of the undesired epimer (11%), whereas **condition B** afforded the desired peptide **27** in a 76% yield without severe racemization (1%). Dipeptide **14** was coupled with **6** under **condition B** to afford the desired tripeptide **28** in a 66% yield (two steps). The coupling of **29** with highly racemizable D-Dpg **7** was performed under **condition B** to afford tetrapeptide **30** in a 49% yield (two steps). The next coupling reaction of tetrapeptide **31** with **8** was performed to afford pentapeptide **32** in a 36% yield (two steps). In this reaction, 31% of the unreacted tetrapeptide **31** was recovered. The subsequent coupling of **33** with highly racemizable D-Dpg **7** under **condition B** using 5.0 equiv. of **7**, and 0.8 equiv. of triphosgene afforded the desired hexapeptide **34** in a 51% yield (two steps). The use of an excess amount of **7** and triphosgene was important for the return of a good yield. In this reaction, 34% of unreacted pentapeptide **33** was recovered. Hexapeptide **35** was coupled with **10b** under **condition B** using 5.0 equiv. of **10b** and 0.8 equiv. of triphosgene, and the subsequent preparative thin layer chromatography separation afforded the pure heptapeptide **3** with no epimer in a 25% yield (two steps). In this reaction, 24% of the unreacted **35** was recovered. In these reactions as well as in other amide bond formations, expensive Hpgs and Dpgs were recovered and reused. It should be noted that peptide chain elongation without severe racemization was achieved based on a linear approach that was considered impossible in feglymycin total synthesis. In addition, the coupling of racemizable amino acids usually requires low temperature and a long reaction time, whereas our developed process allowed rapid coupling (≤5.3 s).

### Total synthesis of feglymycin (**1**)

**Figure 5:**
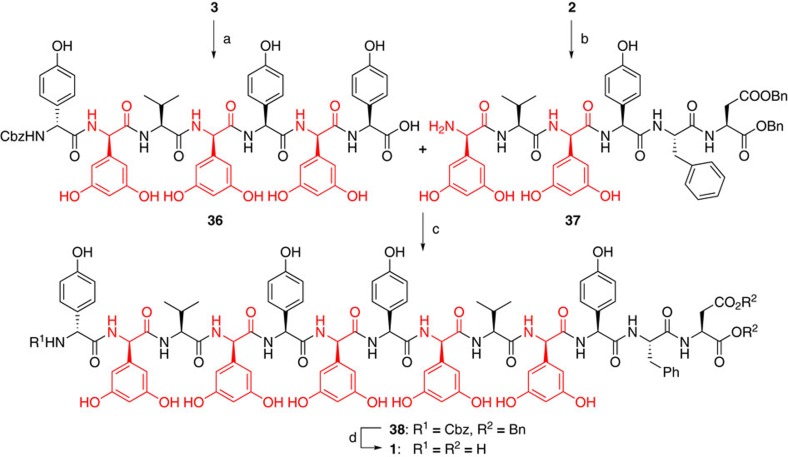
Total synthesis of feglymycin (1). Conditions: (**a**) Me_3_SnOH, 1,2-dichloroethane; 85 °C, 3.5 h; (**b**) PS-Ph_3_P-Pd, PhSiH_3_, CH_2_Cl_2_/MeOH/H_2_O, r.t., 50 min; (**c**) DEPBT, NaHCO_3_, DMF, 0 °C, 24 h, then 25 °C, 11 h, 2 steps 44% from heptapeptide **3**. (**d**) H_2_, Pd/C, MeOH, r.t., 3 h, quant.

Deprotection and coupling of hexapeptide **2** and heptapeptide **3** was performed in accordance with the Süssmuth group report (Fig. 5). The structure of obtained feglymycin (**1**) was confirmed by ^1^H and ^13^C NMR, IR, and HRMS spectra, as well as by specific rotation. The observed spectra were consistent with the previously reported data^12^.

## Discussion

In summary, we demonstrated a total synthesis of feglymycin based on a linear/convergent hybrid synthetic approach. Our originally developed micro-flow amide bond formation enabled the efficient preparation of hexapeptide **2** and heptapeptide **3** containing highly racemizable D-Dpgs based on a linear synthetic approach that was previously thought to be impossible. The developed synthetic approach will be useful for the rapid preparation of feglymycin analogues in the future. Our micro-flow amide bond formation uses triphosgene, and only emits CO_2_ and HCl salt of DIEA. One of the advantages of using microreactors is the ease of scaling up. It should be possible to scale-up our developed process by either continuous operation or by a numbering-up of the microreactors. Our developed process will enable the practical preparation of biologically active oligopeptides containing highly racemizable amino acids.

## Methods

### General

NMR spectra were recorded on JEOL Model ECP-400 (400 MHz for ^1^H, 100 MHz for ^13^C) or Bruker Biospin AVANCE II 400 (400 MHz for ^1^H, 100 MHz for ^13^C), Bruker Biospin AVANCE III HD 500 (500 MHz for ^1^H, 125 MHz for ^13^C) instrument in the indicated solvent. Chemical shifts are reported in units of parts per million (p.p.m.) relative to the signal (0.00 p.p.m.) for internal tetramethylsilane for solutions in CDCl_3_ (7.26 p.p.m. for ^1^H, 77.0 p.p.m. for ^13^C) or DMSO (2.50 p.p.m. for ^1^H, 39.5 p.p.m. for ^13^C). Multiplicities are reported by using the following abbreviations: s; singlet, d; doublet, t; triplet, q; quartet, m; multiplet, br; broad, *J*; coupling constants in Hertz (Hz). IR spectra were recorded on Perkin-Elmer Spectrum One FT-IR spectrometer or JASCO FT/IR-4100. Only the strongest and/or structurally important peaks are reported as IR data given in cm^−1^. Optical rotations were measured using a JASCO P-1020 or P-2200, Rudolph Research Analytical AUTOPOL IV. HRMS (ESI-TOF) were measured with a Bruker micrOTOF II.

All reactions were monitored by thin-layer chromatography carried out on 0.25 mm E. Merck silica gel plates (60F-254) with ultraviolet light, visualized by 10% ethanolic phosphomolybdic acid or 0.5% ninhydrin *n*-butanol solution. Flash column chromatography was performed on Silica Gel 60 N purchased from Kanto Chemical Co or Silica Gel PSQ 60B purchased from Fuji Silysia Chemical LTD. Analytical HPLC was carried out on Shimadzu LC-10AT VP Liquid Chromatograph with a Shimadzu RID-10A Refractive Index Detector and a Shimadzu SPD-10A VP UV–vis Detector, Shimadzu SCL-10A VP System Controller. MeCN was dried using a Glass Contour. DIEA was distilled from ninhydrin and KOH. For HPLC analysis of **18a–c** and **19a–c**, see Supplementary Figs 2–7, and for NMR spectra of synthesized compounds and analysis of **18a–c** and **19a–c**, see Supplementary Figs 8–45.

### Data availability

The authors declare that the data supporting the findings of this study are available within the article (and its Supplementary Information files). The data supporting the findings of this study are available from the authors upon reasonable request.

## Additional information

**How to cite this article:** Fuse, S. *et al*. Total synthesis of feglymycin based on a linear/convergent hybrid approach using micro-flow amide bond formation. *Nat. Commun.* **7,** 13491 doi: 10.1038/ncomms13491 (2016).

**Publisher's note**: Springer Nature remains neutral with regard to jurisdictional claims in published maps and institutional affiliations.

## Supplementary Material

Supplementary Information Supplementary Figures 1-45 and Supplementary Methods.

## Figures and Tables

**Table 1:**
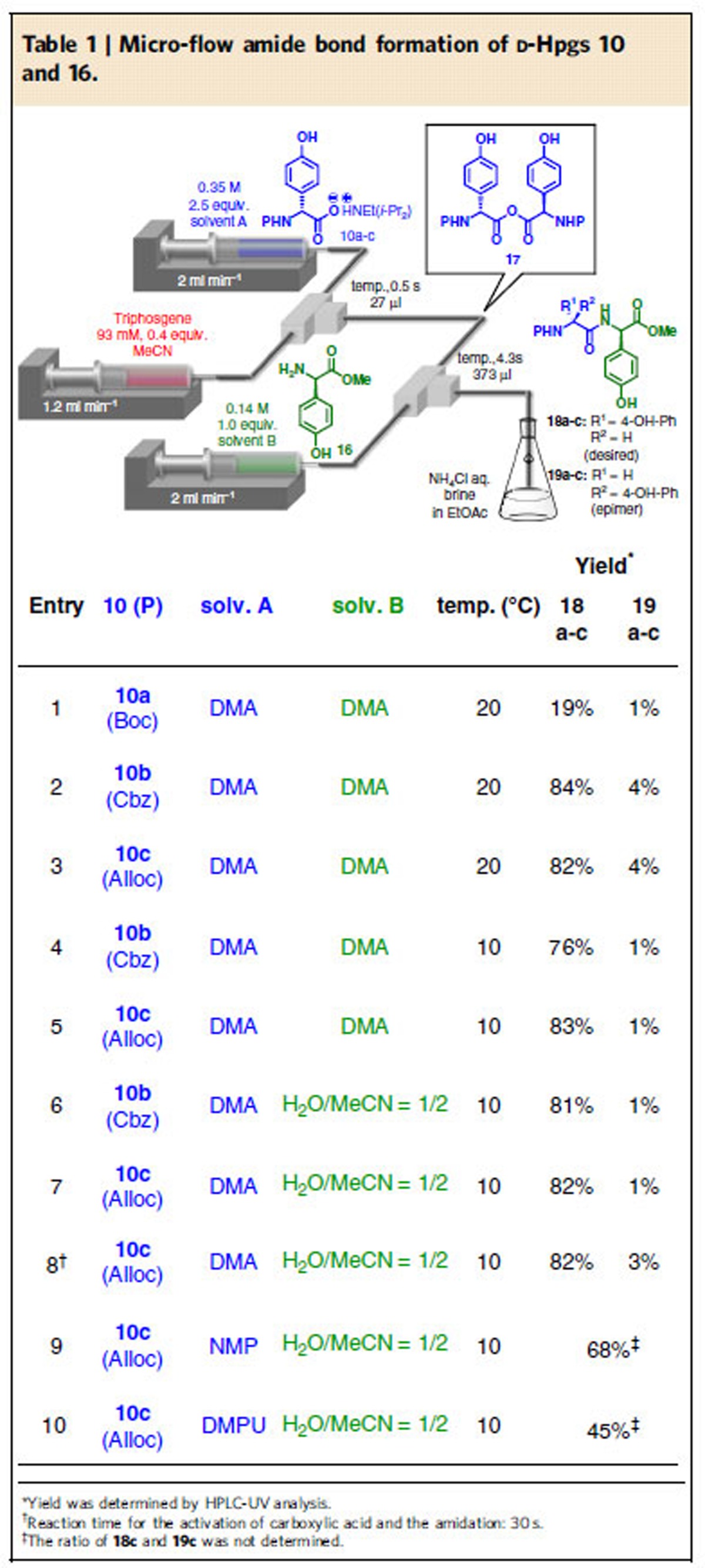
Micro-flow amide bond formation of D-Hpgs 10 and 16.
