# Supplementary material for: Total synthesis of feglymycin based on a linear/convergent hybrid approach using micro-flow amide bond formation
Source: Nat Commun. 2016 Nov 28;7:13491. doi: 10.1038/ncomms13491 (PMC5133696; doi:10.1038/ncomms13491)
Supplement: Supplementary Information — Supplementary Figures 1-45 and Supplementary Methods. [file ncomms13491-s1.pdf]

Supplementary Figure 1. Micro-flow reactor setup

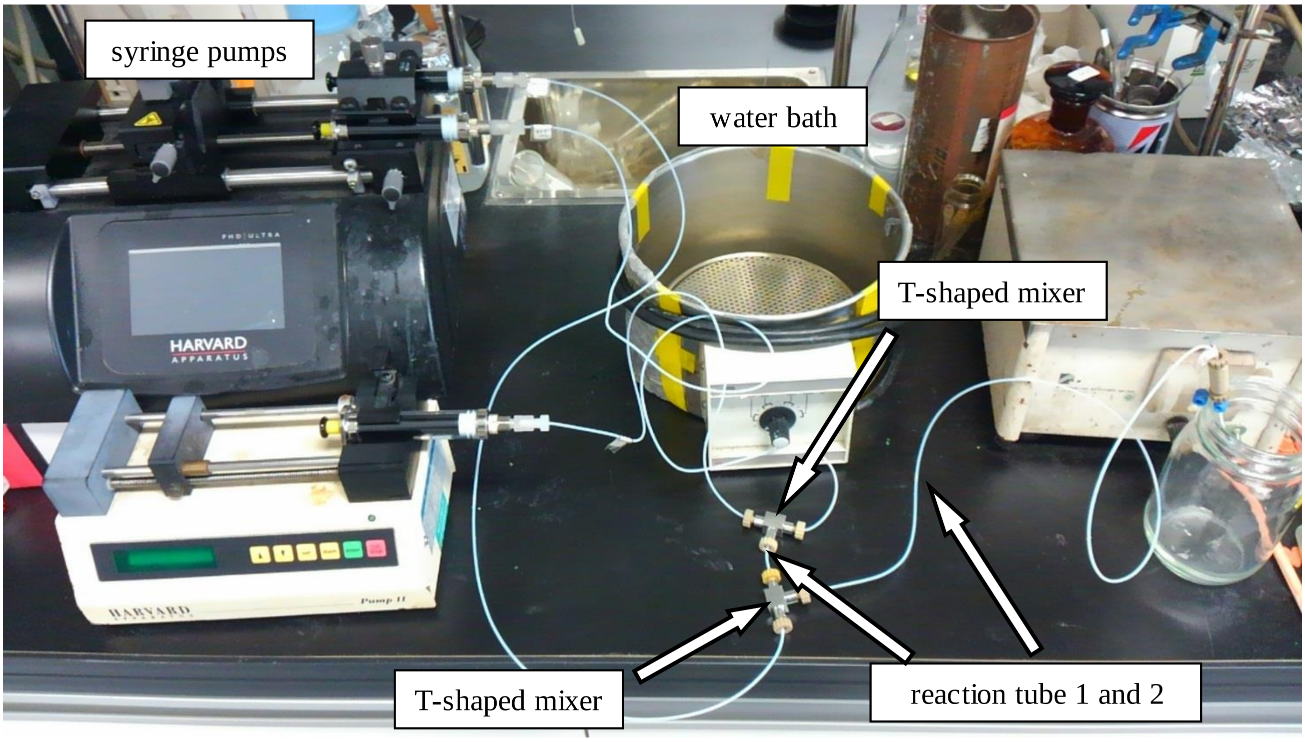

Supplementary Figure 2. HPLC chart of Boc-D-Hpg-D-Hpg-OMe (**18a**)

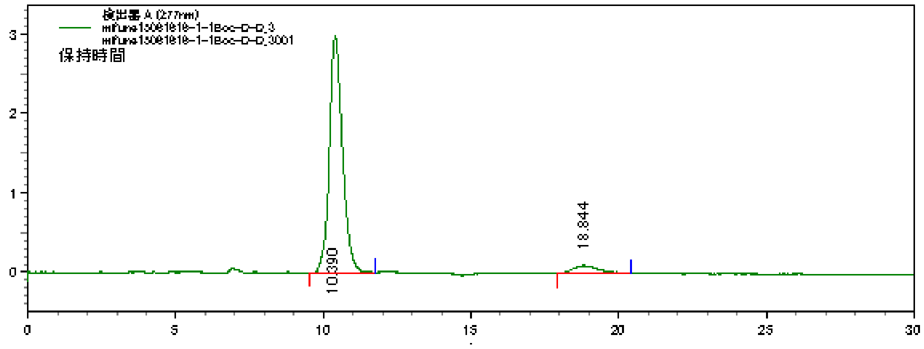

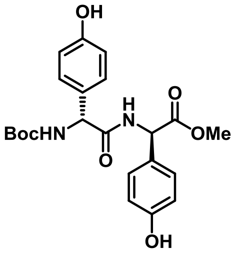

18a

Supplementary Figure 3. HPLC chart of Boc-L-Hpg-D-Hpg-OMe (**19a**)

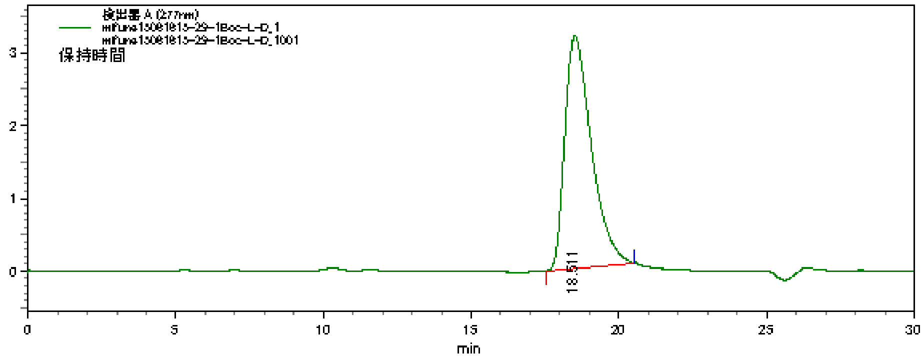

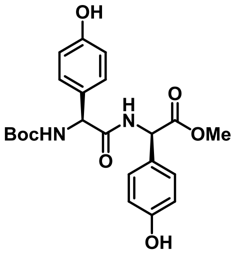

19a

Supplementary Figure 4. HPLC chart of Cbz-D-Hpg-D-Hpg-OMe (**18b**)

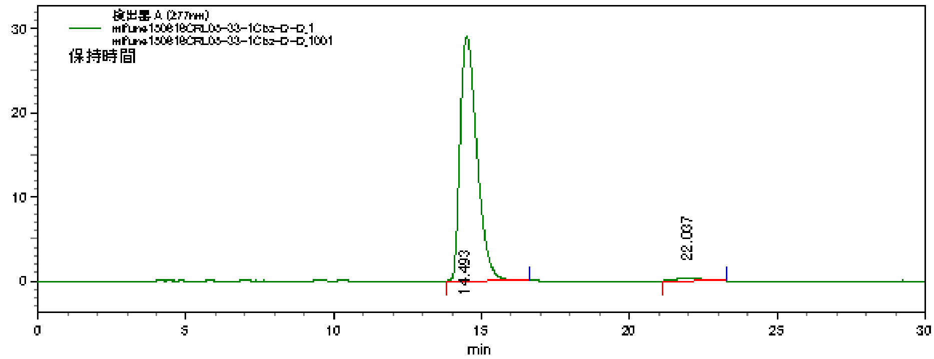

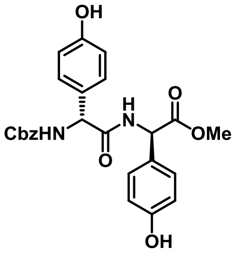

18b

Supplementary Figure 5. HPLC chart of Cbz-L-Hpg-D-Hpg-OMe (**19b**)

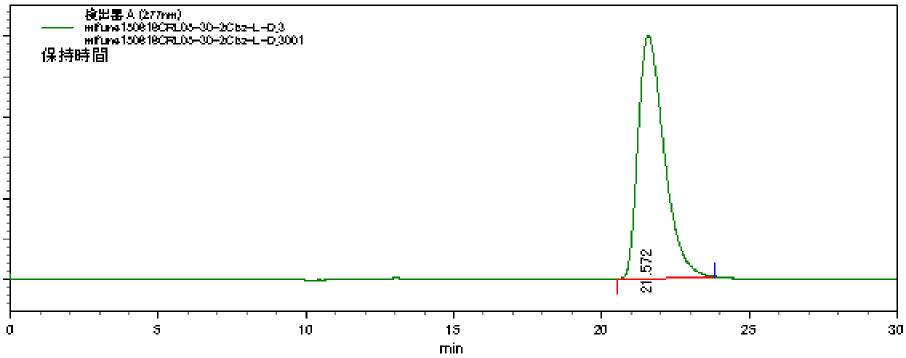

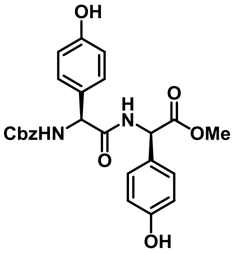

19b

Supplementary Figure 6. HPLC chart of Alloc-D-Hpg-D-Hpg-OMe (**18c**)

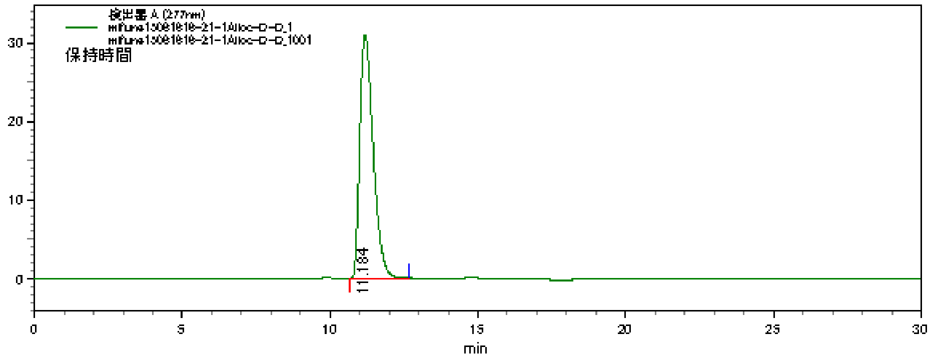

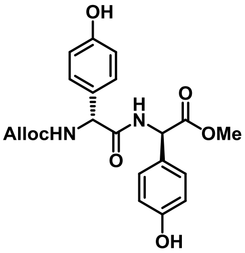

18c

Supplementary Figure 7. HPLC chart of Alloc-L-Hpg-D-Hpg-OMe (**19c**)

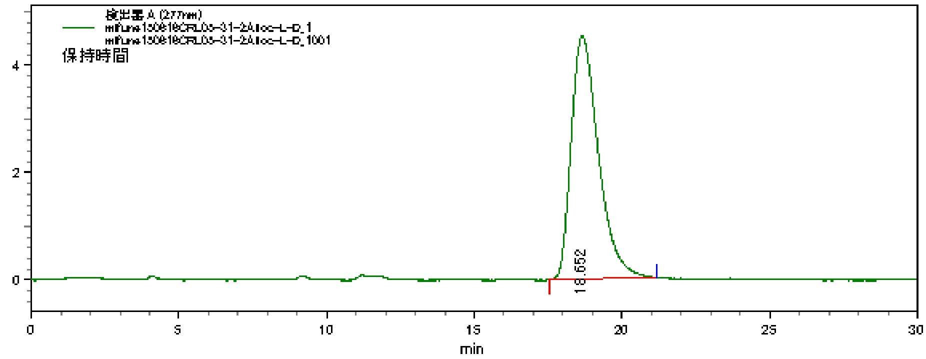

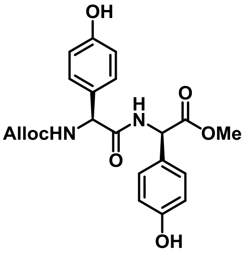

19c

Supplementary Figure 8. ^{1}H NMR spectrum of Boc-D-Hpg-D-Hpg-OMe (**18a**)

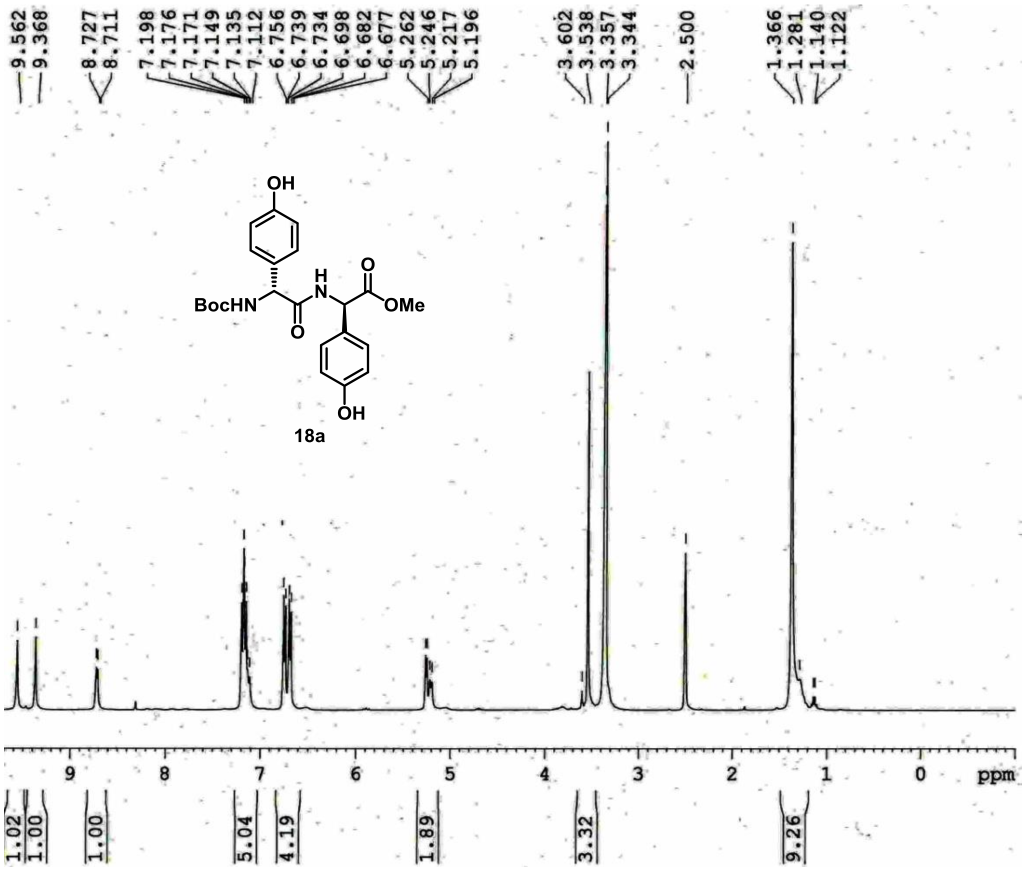

Supplementary Figure 9. ^{13}C NMR spectrum of Boc-D-Hpg-D-Hpg-OMe (**18a**)

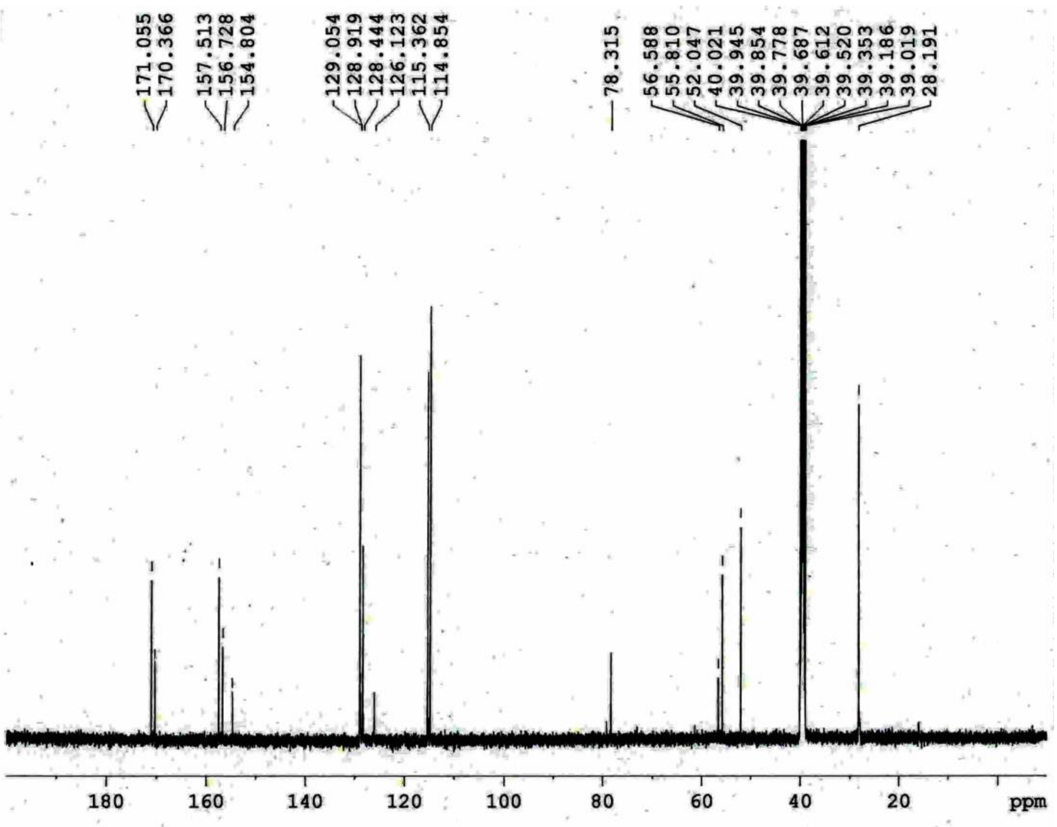

Supplementary Figure 10. ^{1}H NMR spectrum of Cbz-D-Hpg-D-Hpg-OMe (**18b**)

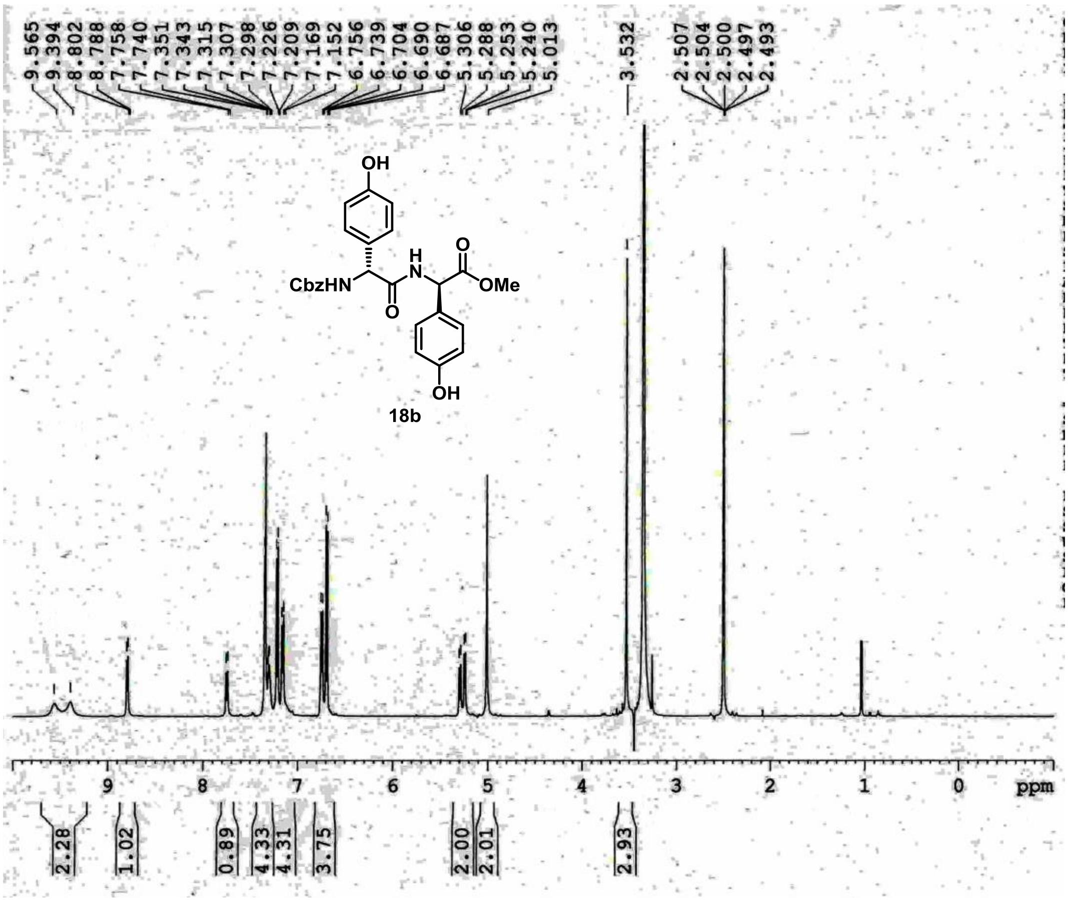

Supplementary Figure 11. ^{13}C NMR spectrum of Cbz-D-Hpg-D-Hpg-OMe (**18b**)

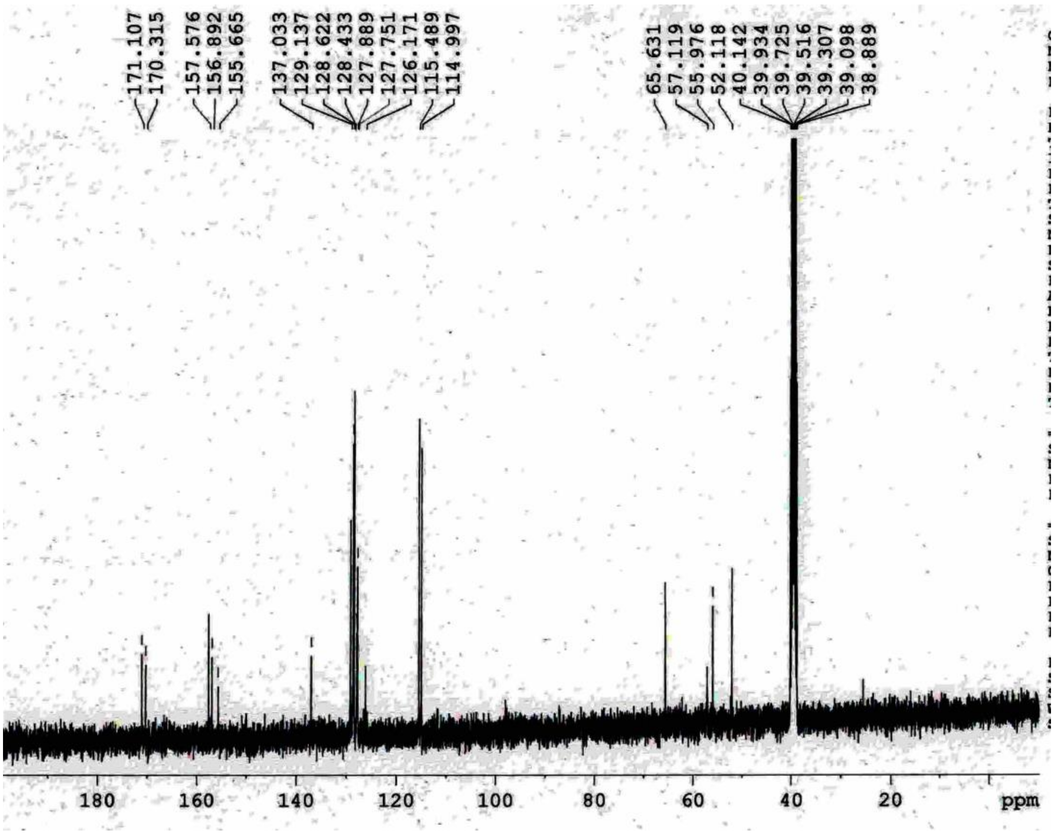

Supplementary Figure 12. ^{1}H NMR spectrum of Alloc-D-Hpg-D-Hpg-OMe (**18c**)

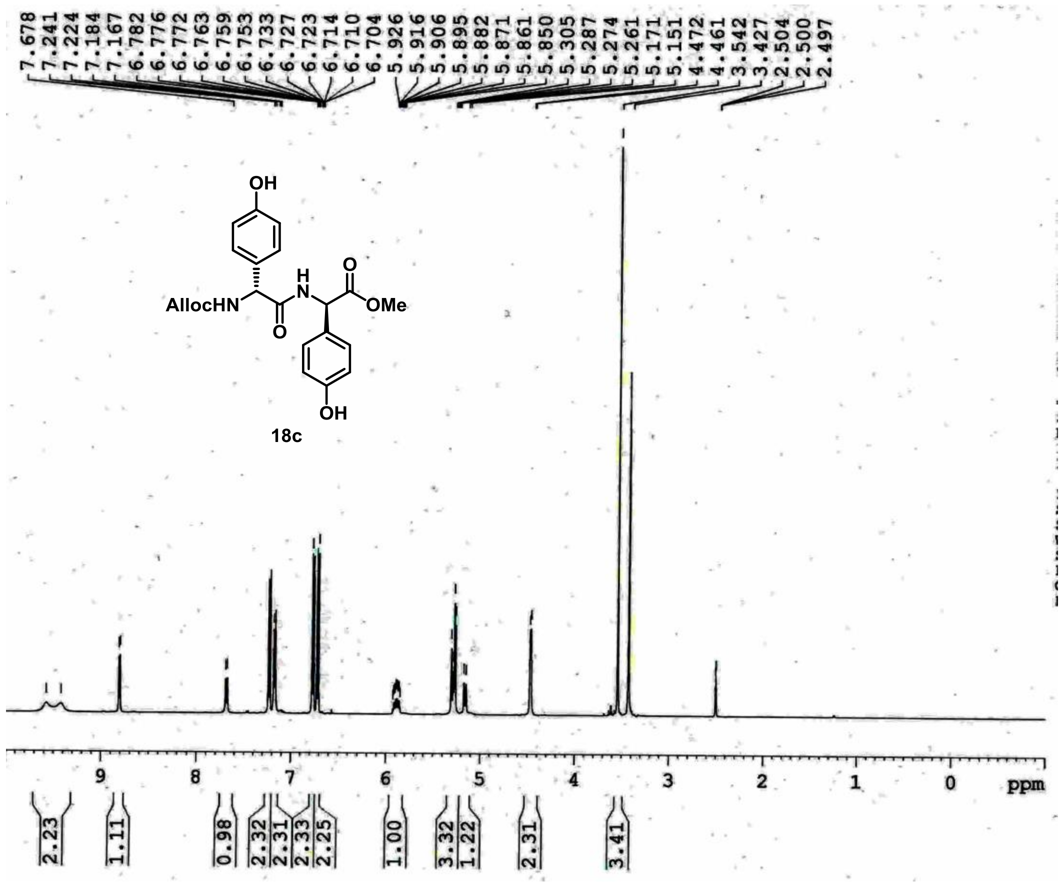

Supplementary Figure 13. ^{13}C NMR spectrum of Alloc-D-Hpg-D-Hpg-OMe (**18c**)

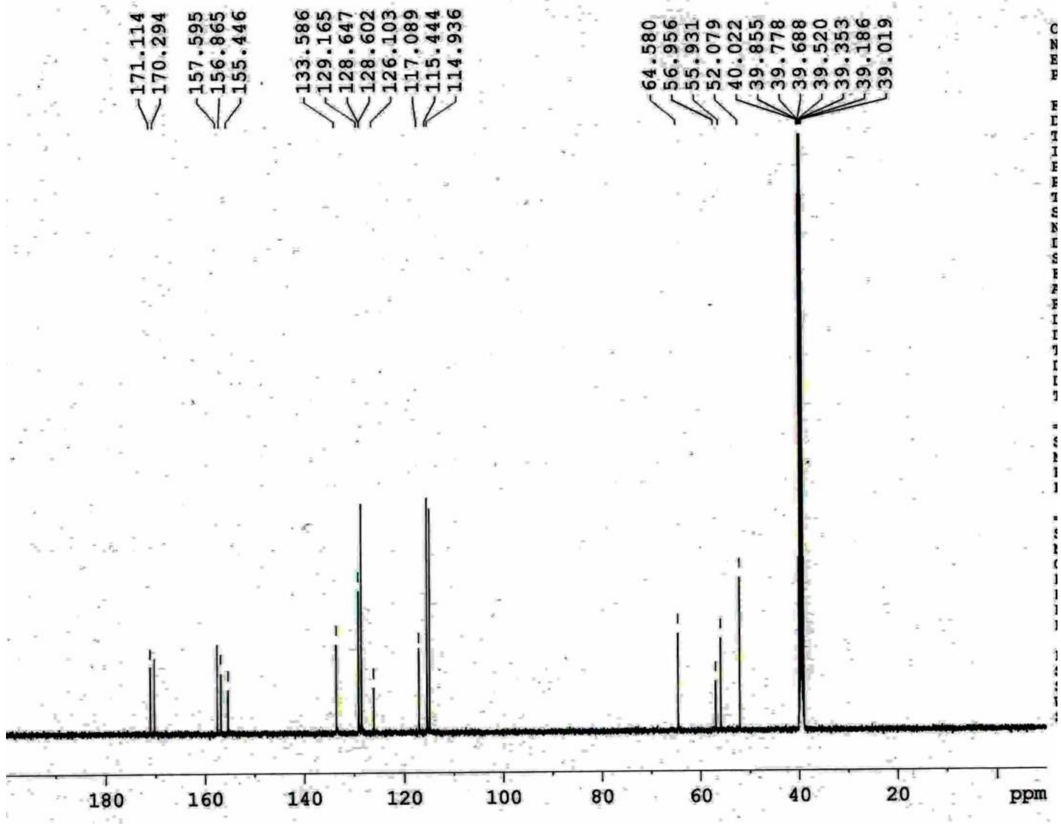

Supplementary Figure 14. ^{1}H NMR spectrum of Boc-L-Hpg-D-Hpg-OMe (**19a**)

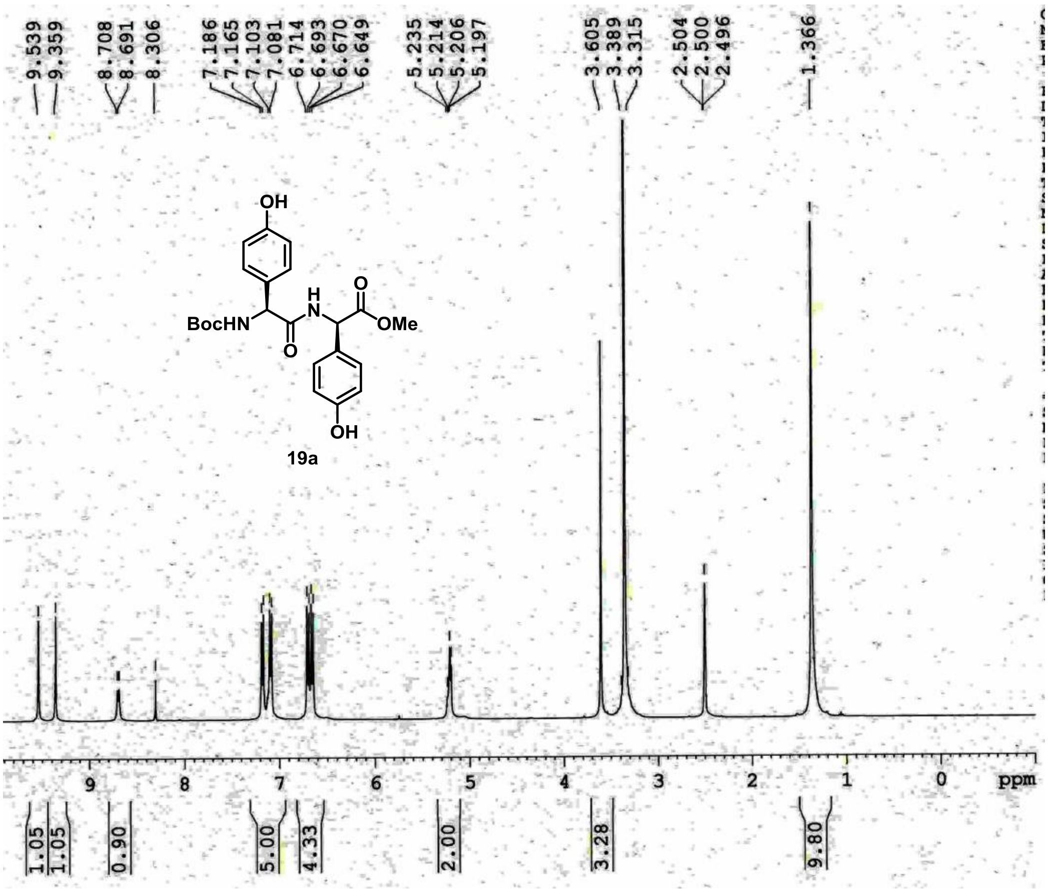

Supplementary Figure 15. ^{13}C NMR spectrum of Boc-L-Hpg-D-Hpg-OMe (**19a**)

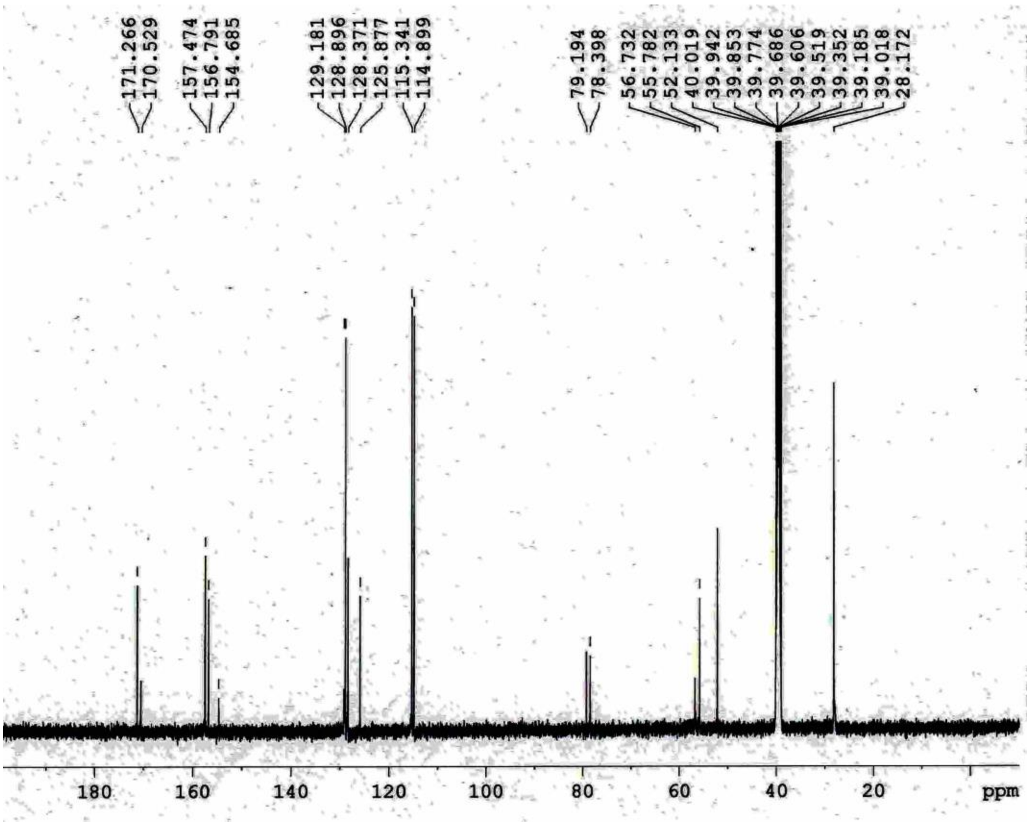

Supplementary Figure 16. ^{1}H NMR spectrum of Cbz-L-Hpg-D-Hpg-OMe (**19b**)

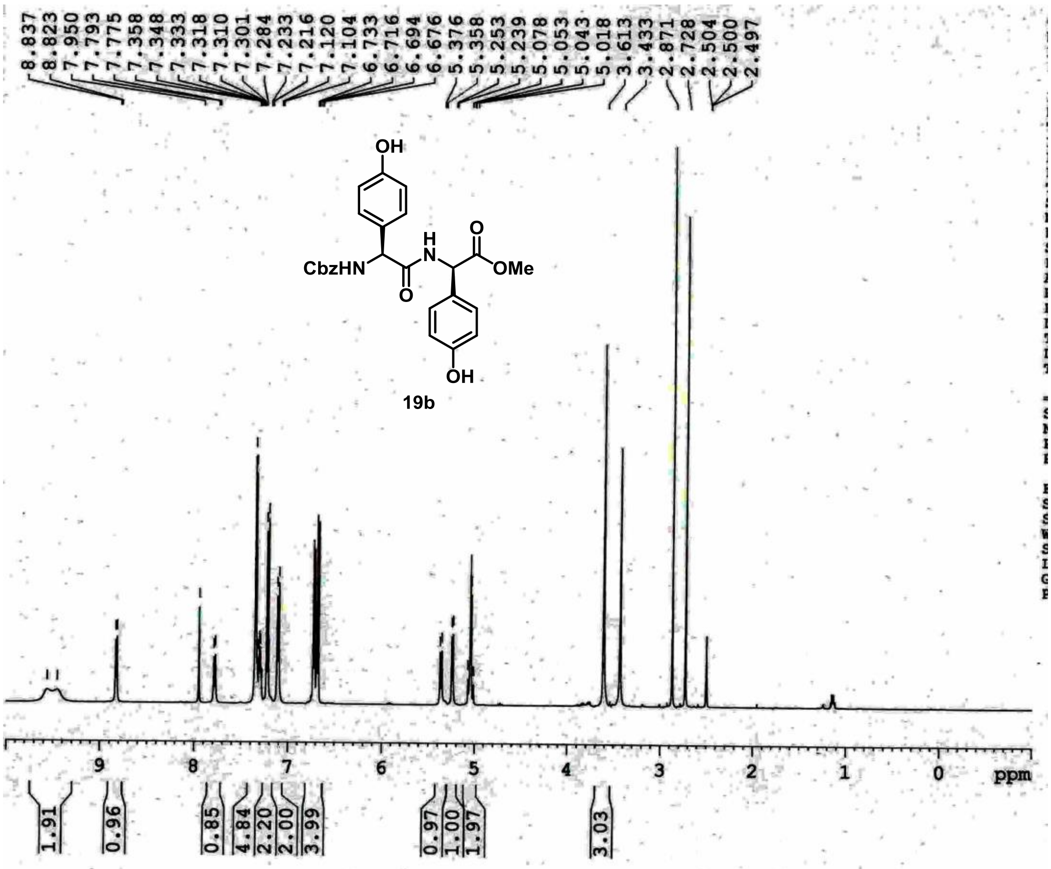

Supplementary Figure 17. ^{13}C NMR spectrum of Cbz-L-Hpg-D-Hpg-OMe (**19b**)

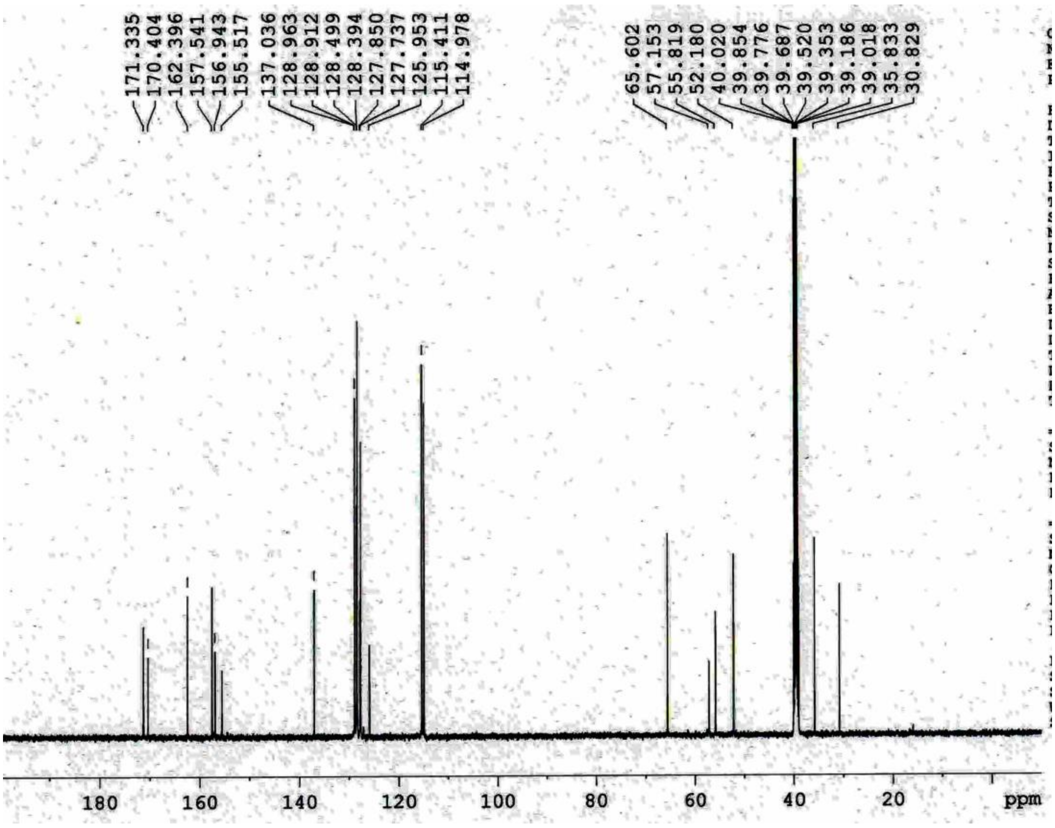

Supplementary Figure 18. ^{1}H NMR spectrum of Alloc-L-Hpg-D-Hpg-OMe (**19c**)

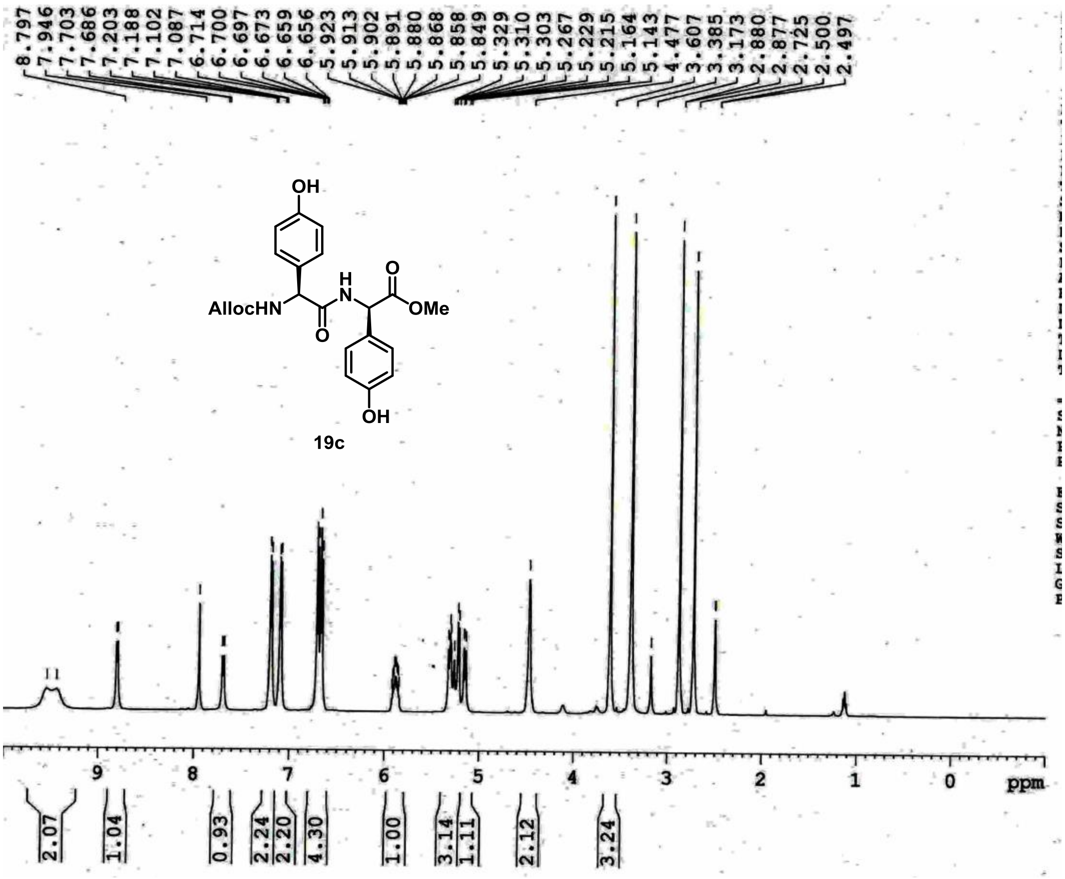

Supplementary Figure 19. ^{13}C NMR spectrum of Alloc-L-Hpg-D-Hpg-OMe (**19c**)

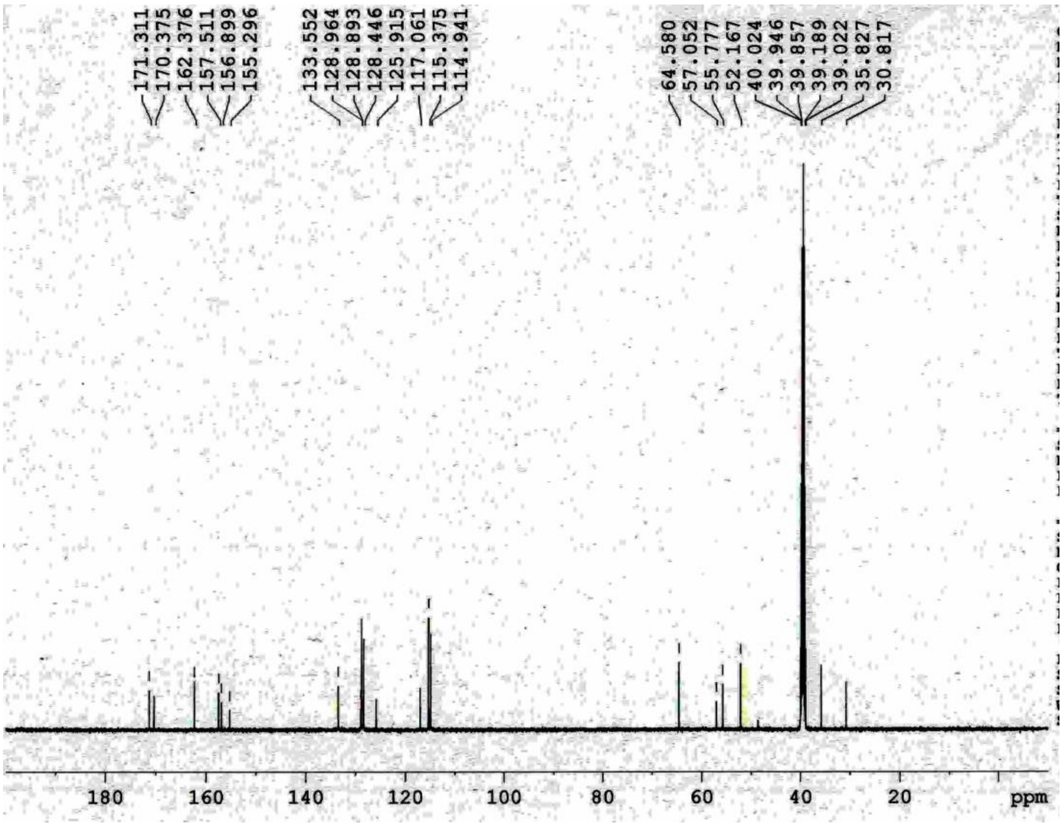

Supplementary Figure 20. ^{1}H NMR spectrum of Alloc-L-Phe-L-Asp(*O*-Bn)-OBn (**20**)

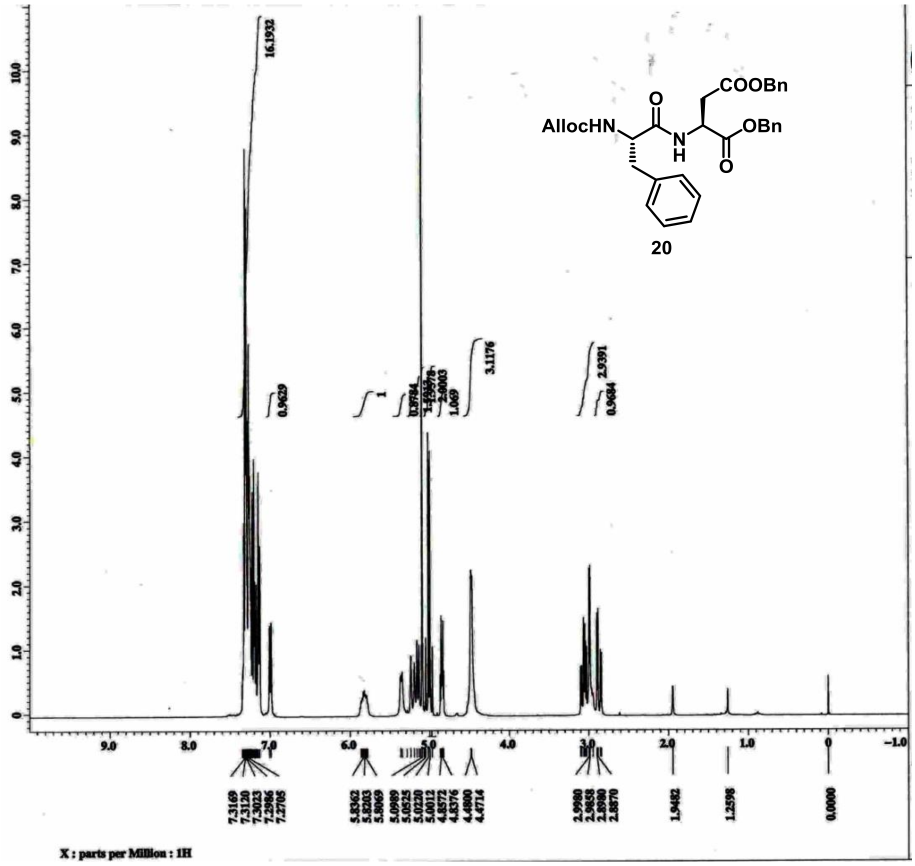

Supplementary Figure 21. ^{13}C NMR spectrum of Alloc-L-Phe-L-Asp(*O*-Bn)-OBn (**20**)

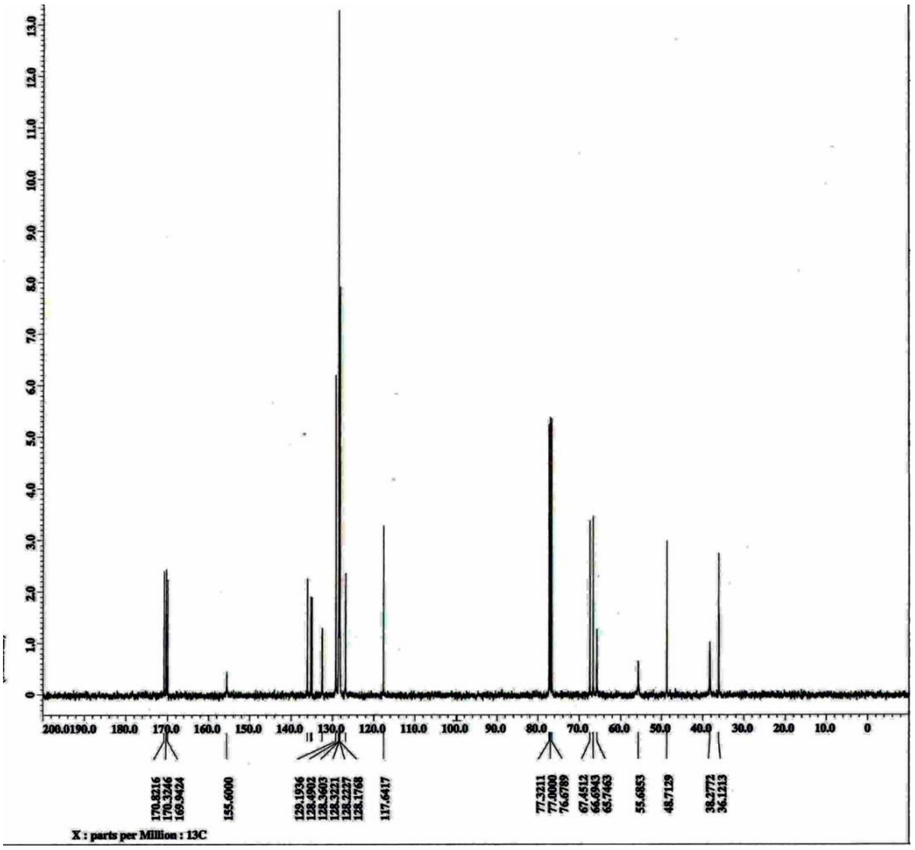

Supplementary Figure 22. ^{1}H NMR spectrum of Alloc-L-Hpg-L-Phe-L-Asp(*O*-Bn)-OBn (**21**)

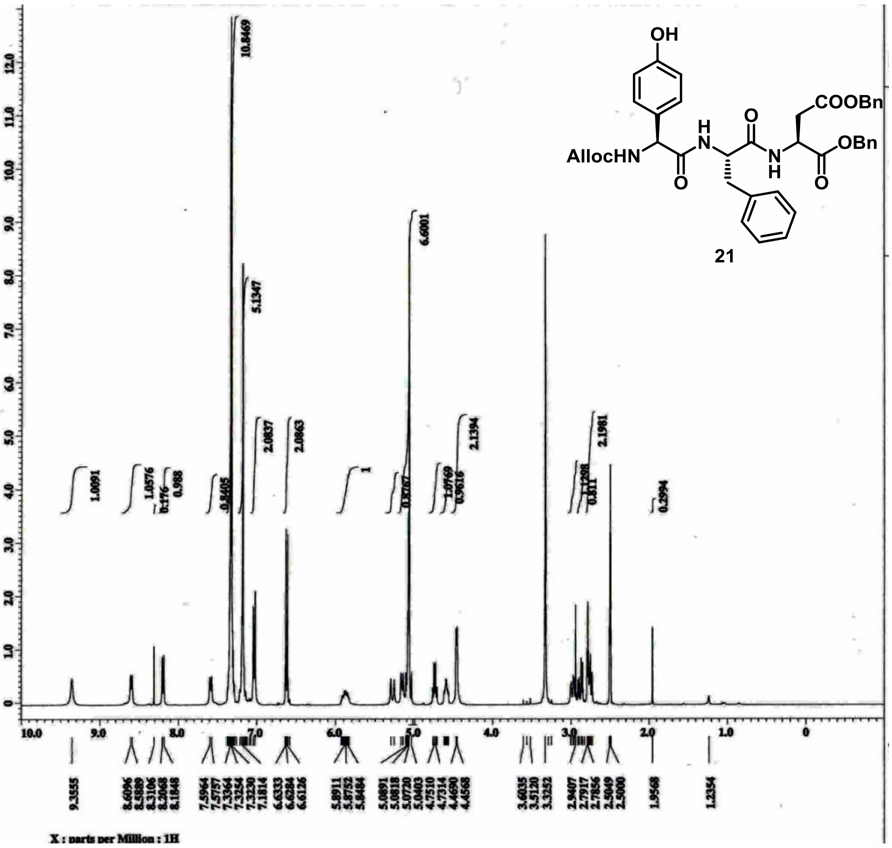

Supplementary Figure 23. ^{13}C NMR spectrum of Alloc-L-Hpg-L-Phe-L-Asp(*O*-Bn)-OBn (**21**)

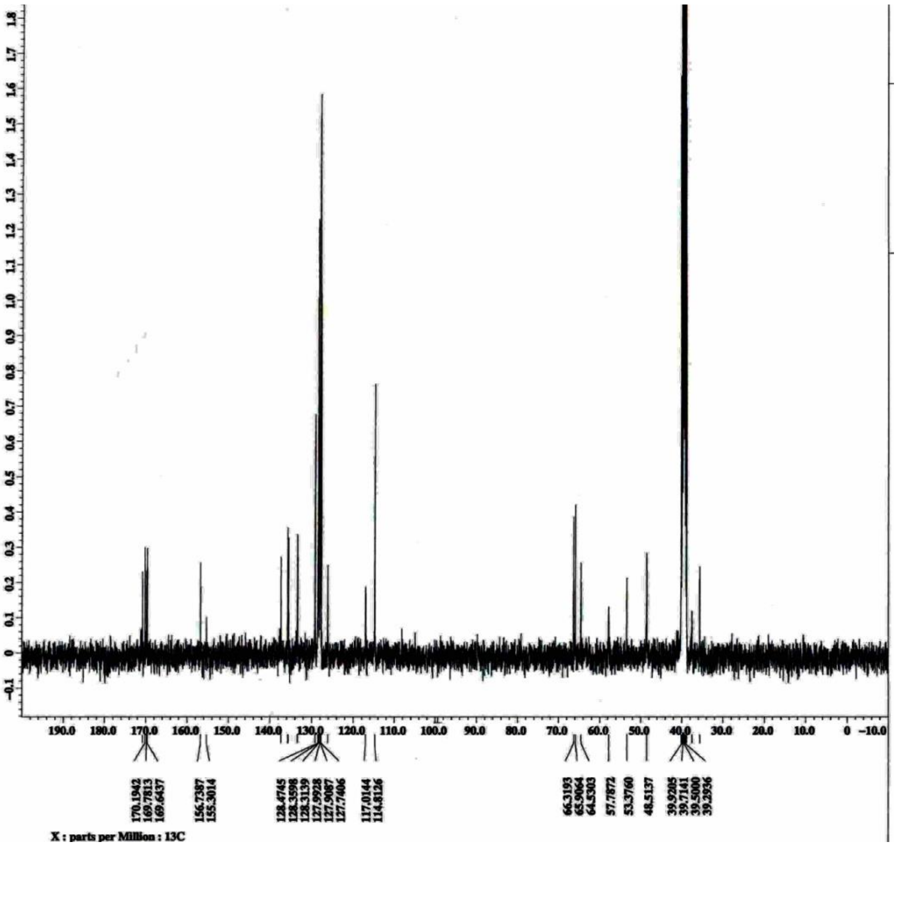

Supplementary Figure 24. ^{1}H NMR spectrum of Alloc-D-Dpg-L-Hpg-L-Phe-L-Asp(*O*-Bn)-OBn (**23**)

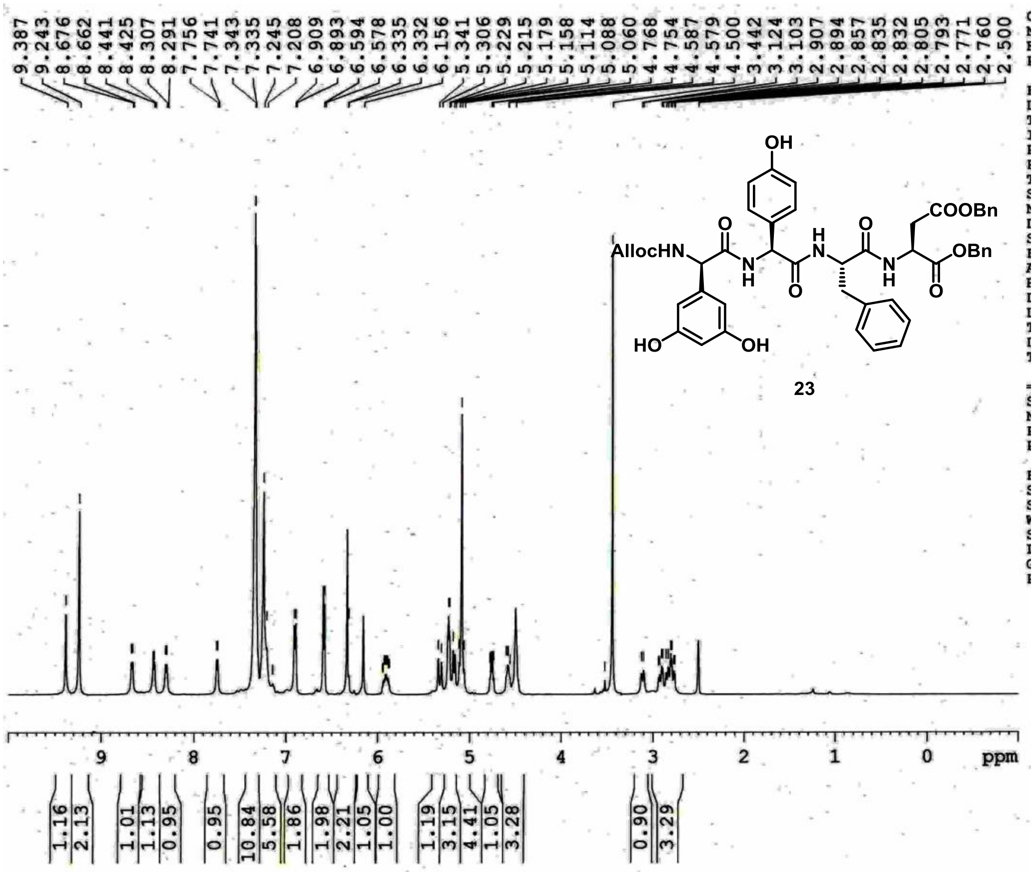

Supplementary Figure 25. ^{13}C NMR spectrum of Alloc-D-Dpg-L-Hpg-L-Phe-L-Asp(*O*-Bn)-OBn (**23**)

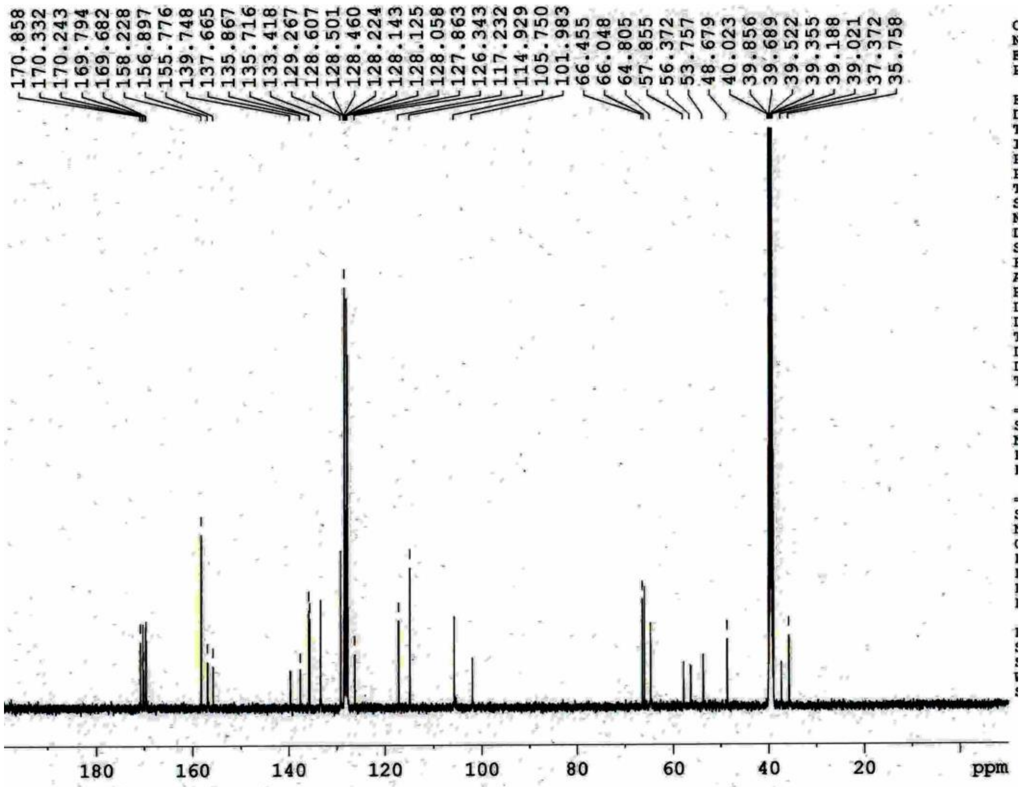

Supplementary Figure 26. ^{1}H NMR spectrum of Alloc-L-Val-D-Dpg-L-Hpg-L-Phe-L-Asp(*O*-Bn)-OBn (**25**)

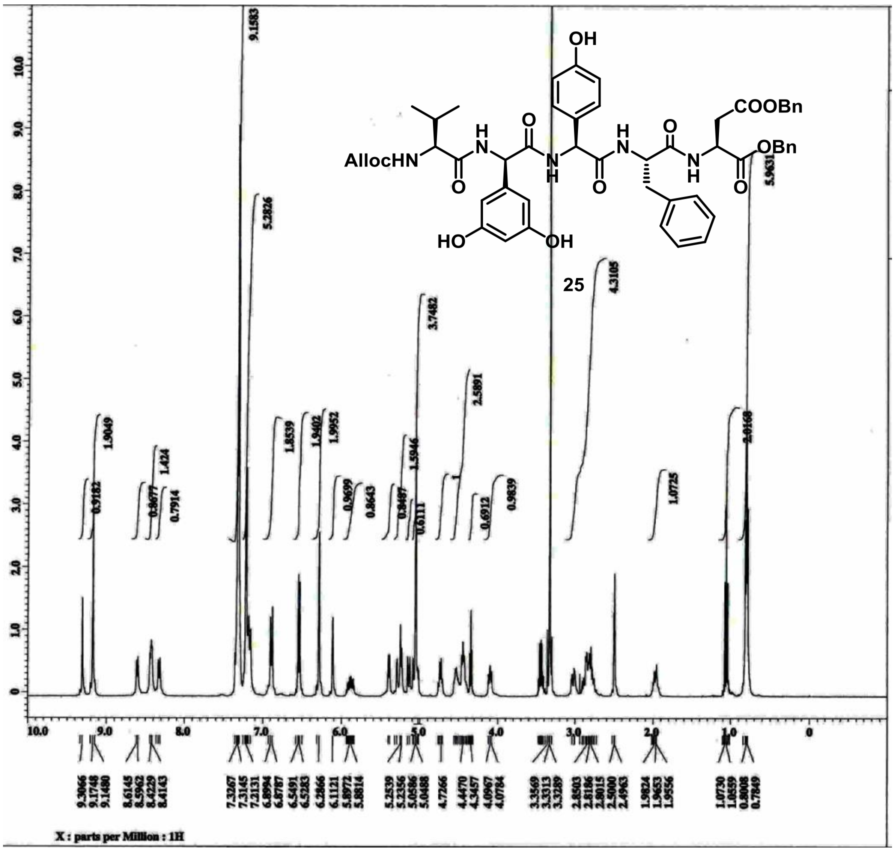

Supplementary Figure 27. ^{13}C NMR spectrum of Alloc-L-Val-D-Dpg-L-Hpg-L-Phe-L-Asp(*O*-Bn)-OBn (**25**)

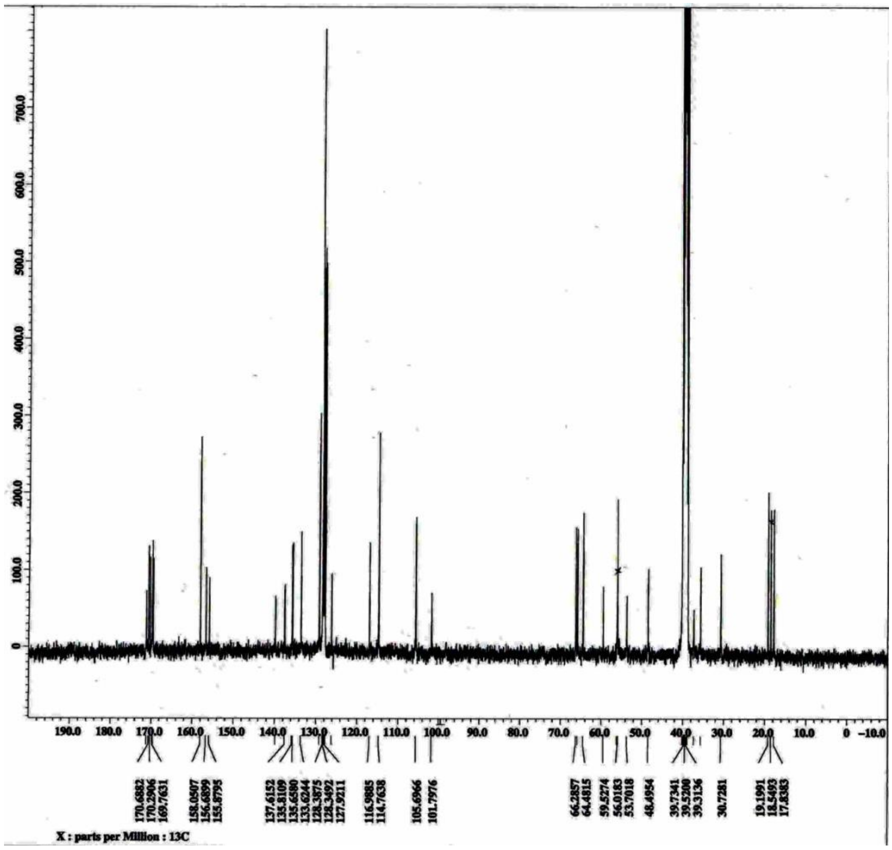

Supplementary Figure 28. ^{1}H NMR spectrum of Alloc-D-Dpg-L-Val-D-Dpg-L-Hpg-L-Phe-L-Asp(*O*-Bn)-OBn (**2**)

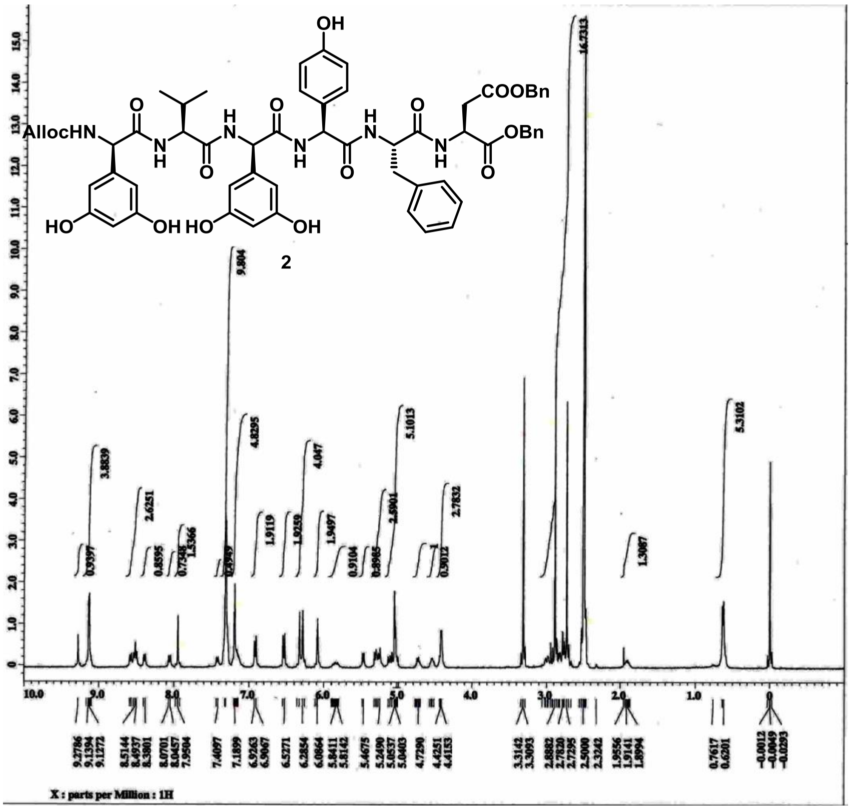

Supplementary Figure 29. ^{13}C NMR spectrum of Alloc-D-Dpg-L-Val-D-Dpg-L-Hpg-L-Phe-L-Asp(*O*-Bn)-OBn (**2**)

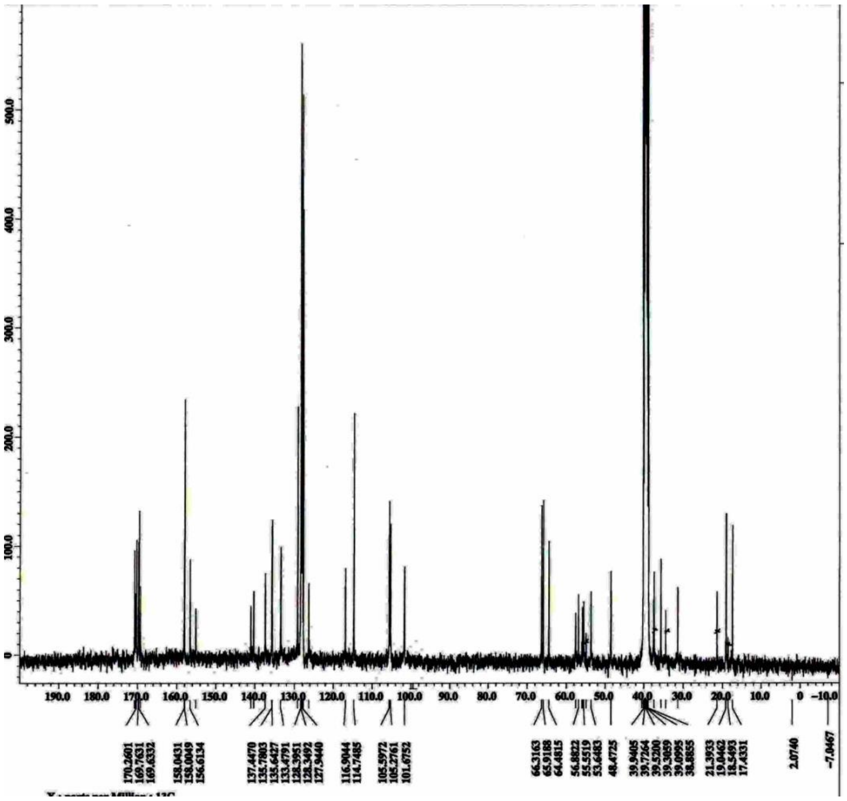

Supplementary Figure 30. ^{1}H NMR spectrum of Alloc-D-Dpg-L-Hpg-OMe (**27**)

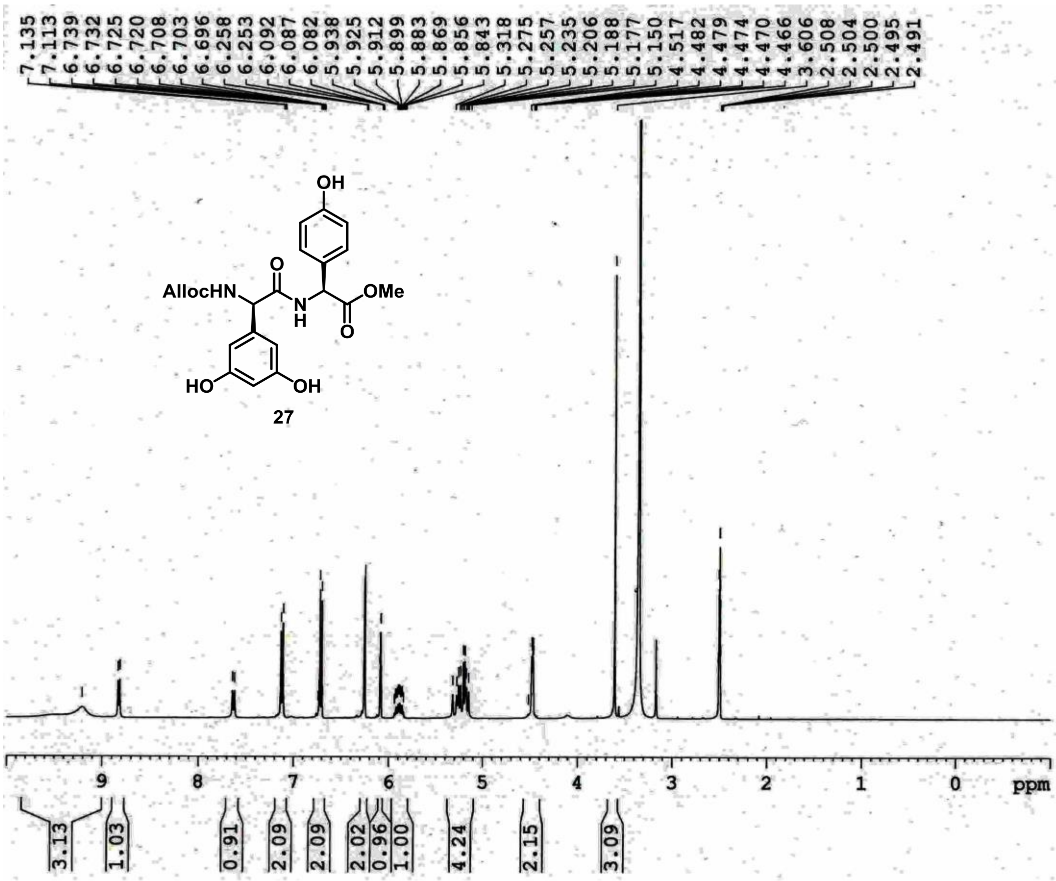

Supplementary Figure 31. ^{13}C NMR spectrum of Alloc-D-Dpg-L-Hpg-OMe (**27**)

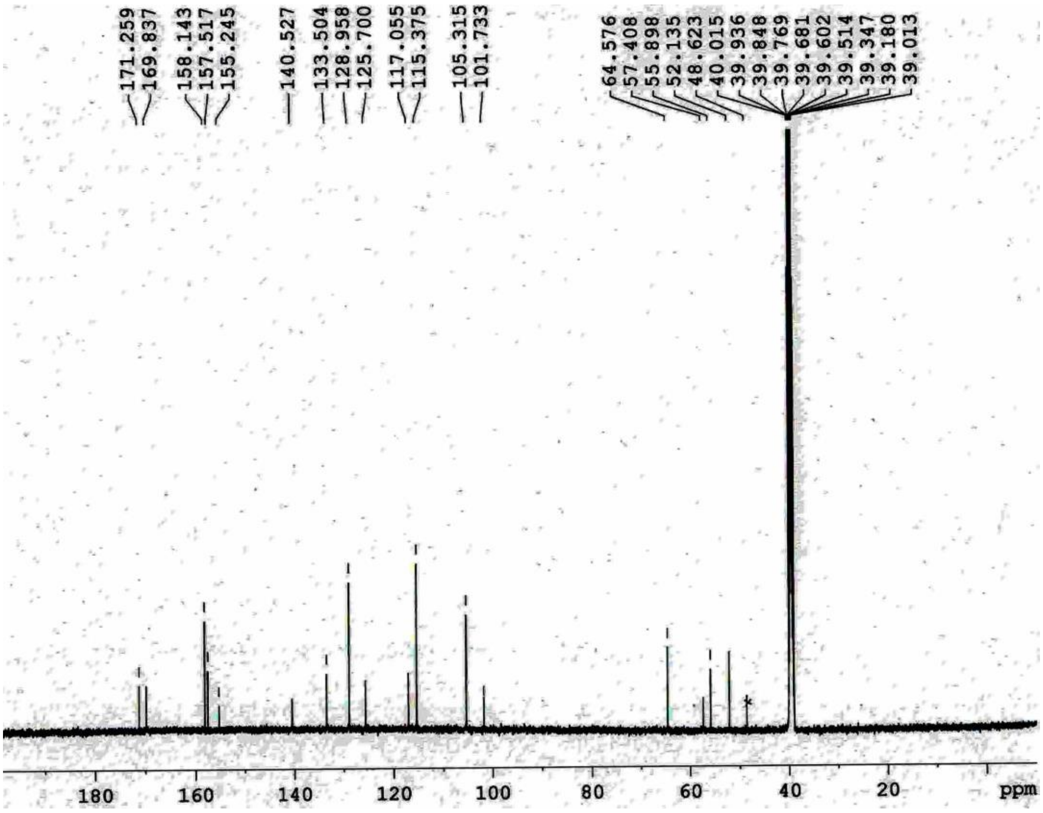

Supplementary Figure 32. ^{1}H NMR spectrum of Alloc-L-Hpg-D-Dpg-L-Hpg-OMe (**28**)

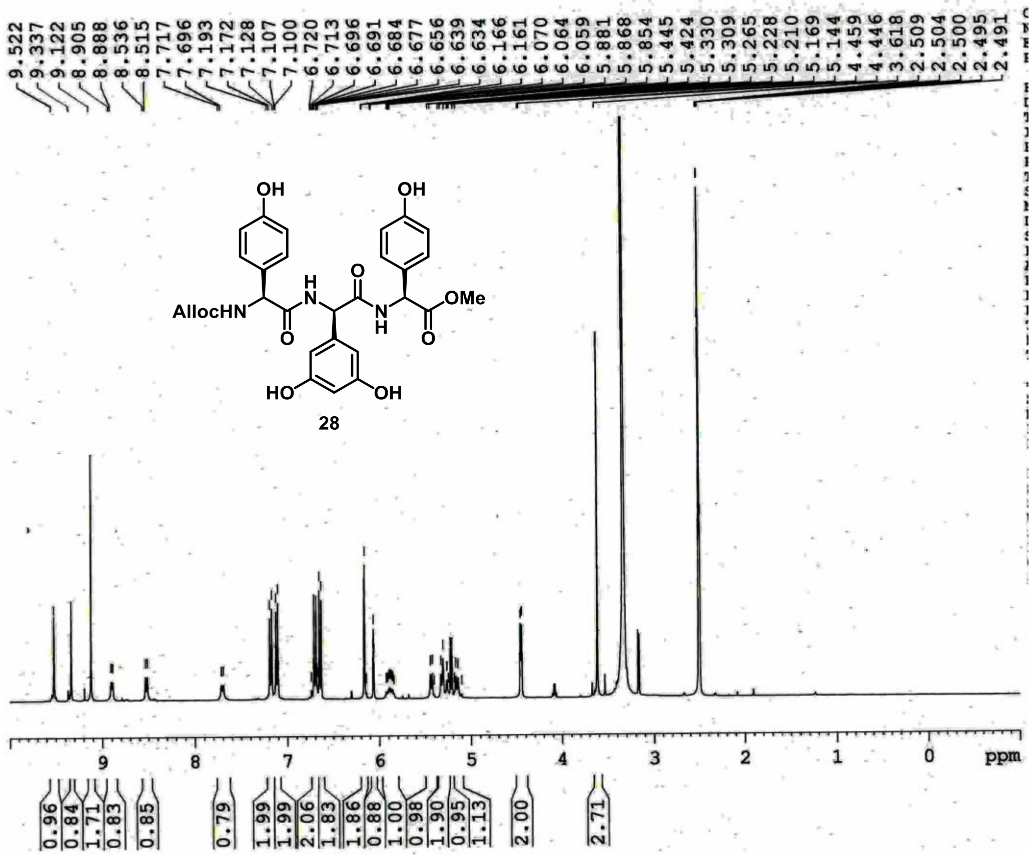

Supplementary Figure 33. ^{13}C NMR spectrum of Alloc-L-Hpg-D-Dpg-L-Hpg-OMe (**28**)

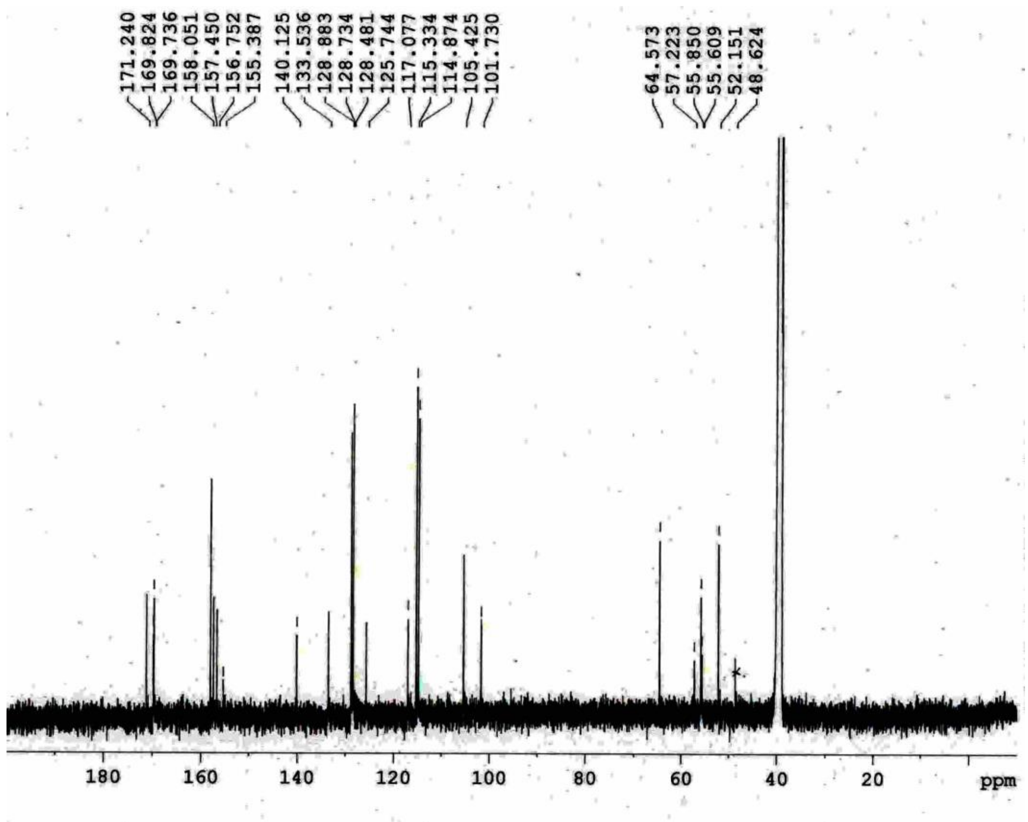

Supplementary Figure 34. ^{1}H NMR spectrum of Alloc-D-Dpg-L-Hpg-D-Dpg-L-Hpg-OMe (**30**)

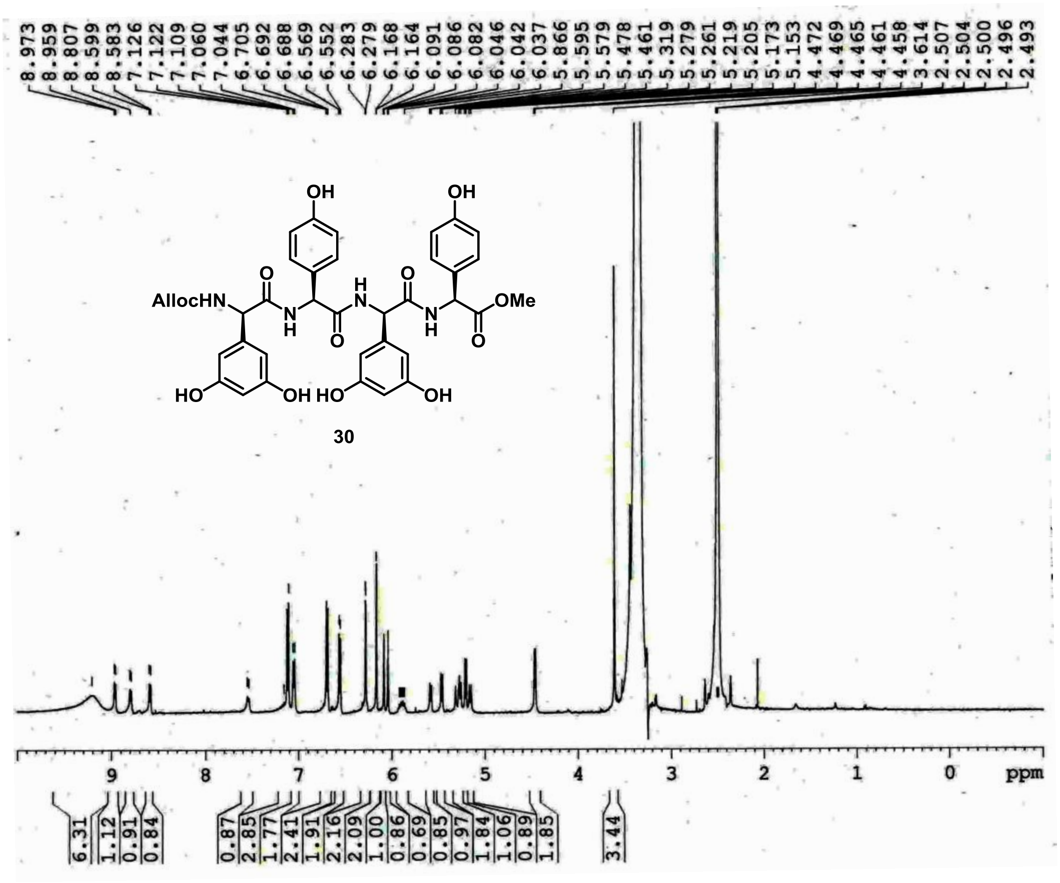

Supplementary Figure 35. ^{13}C NMR spectrum of Alloc-D-Dpg-L-Hpg-D-Dpg-L-Hpg-OMe (**30**)

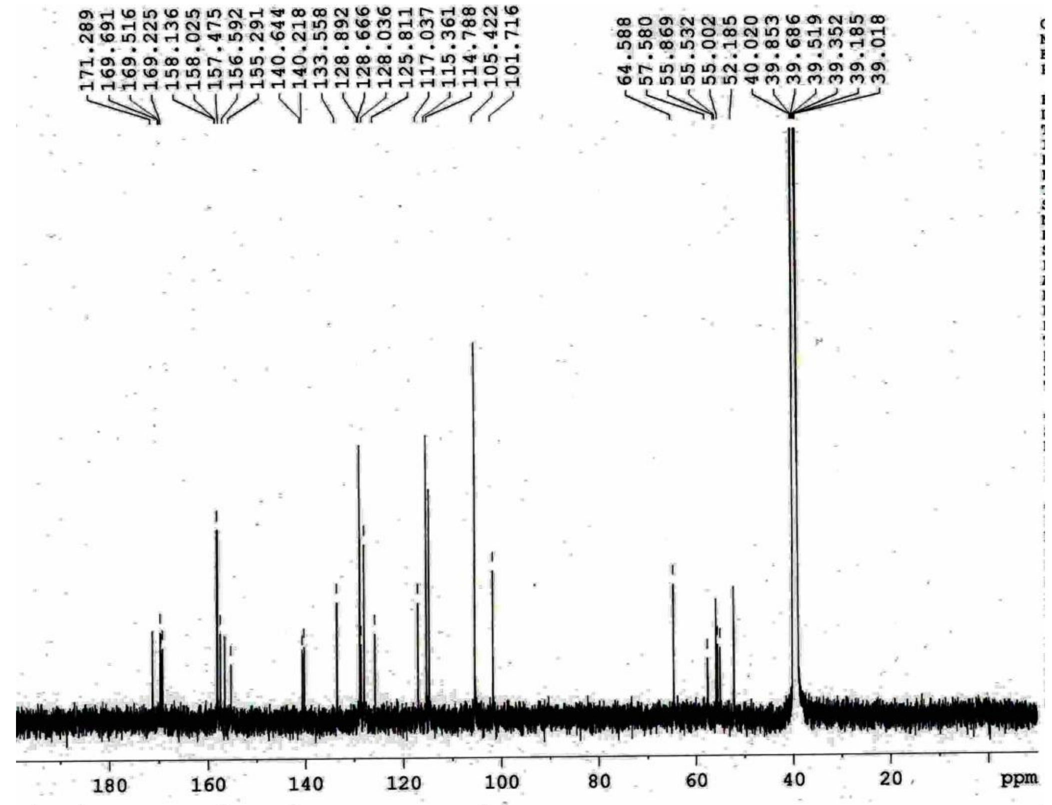

Supplementary Figure 36. ^{1}H NMR spectrum of Alloc-L-Val-D-Dpg-L-Hpg-D-Dpg-L-Hpg-OMe (**32**)

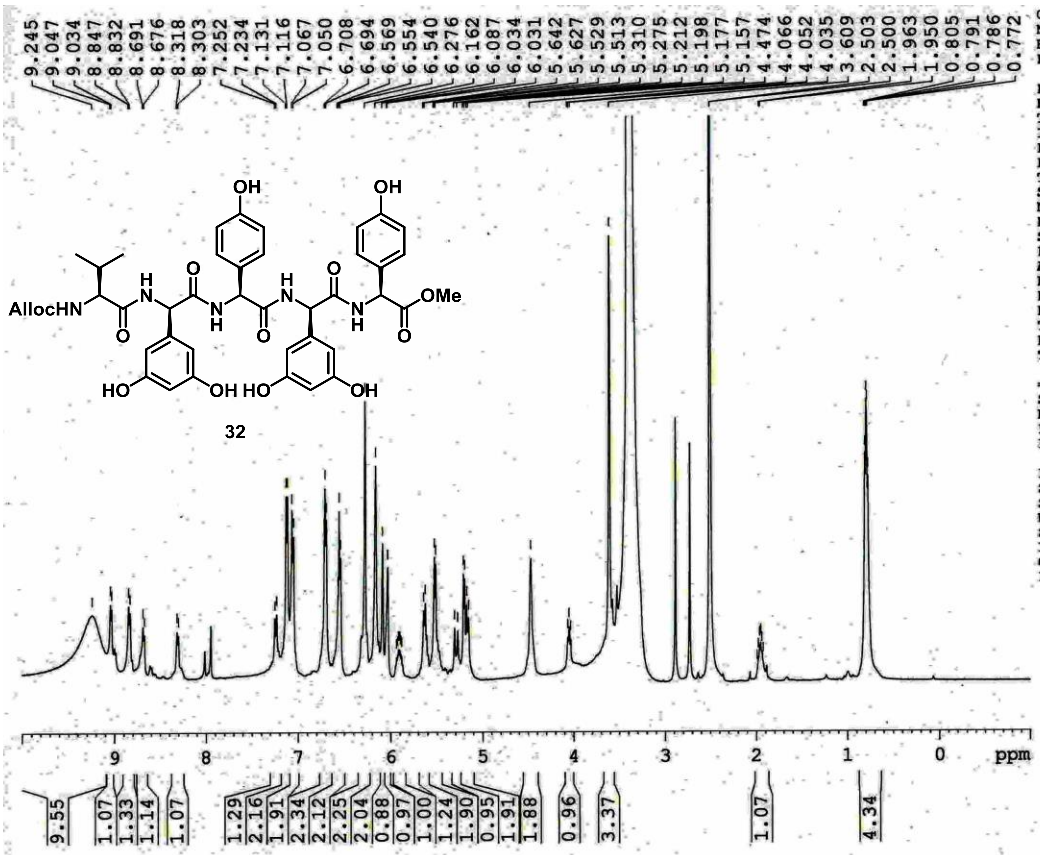

Supplementary Figure 37. ^{13}C NMR spectrum of Alloc-L-Val-D-Dpg-L-Hpg-D-Dpg-L-Hpg-OMe (**32**)

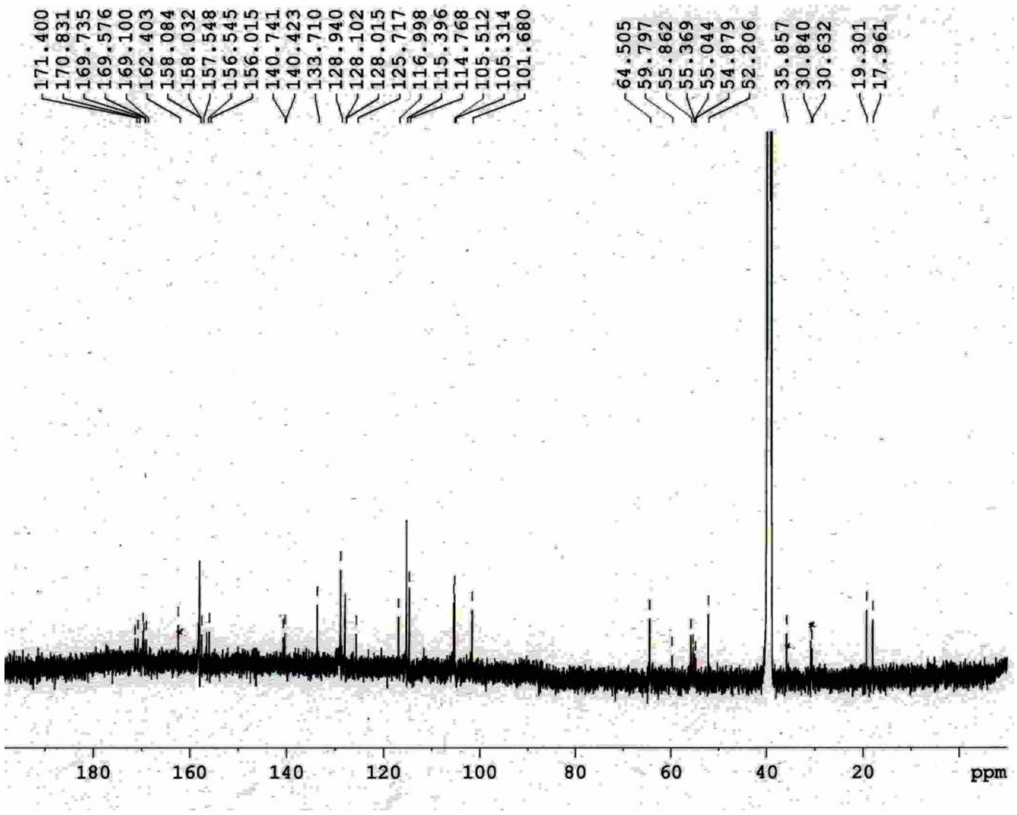

Supplementary Figure 38. ^{1}H NMR spectrum of Alloc-D-Dpg-L-Val-D-Dpg-L-Hpg-D-Dpg-L-Hpg-OMe (**34**)

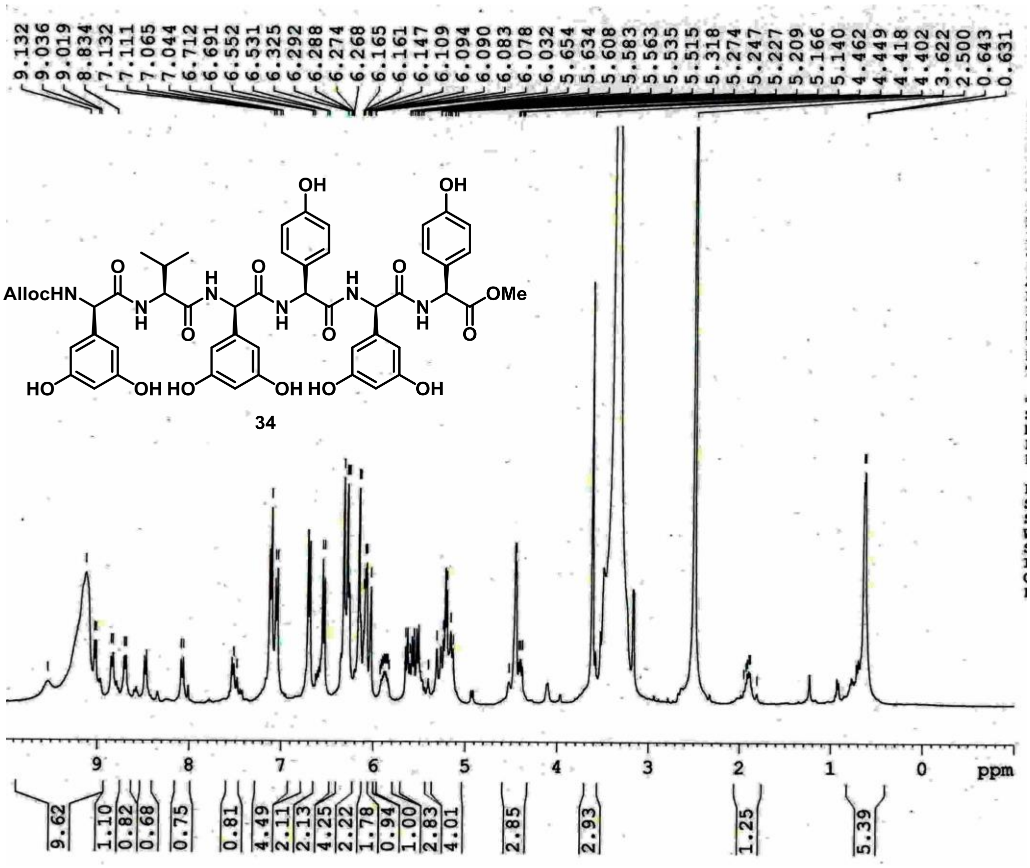

Supplementary Figure 39. ^{13}C NMR spectrum of Alloc-D-Dpg-L-Val-D-Dpg-L-Hpg-D-Dpg-L-Hpg-OMe (**34**)

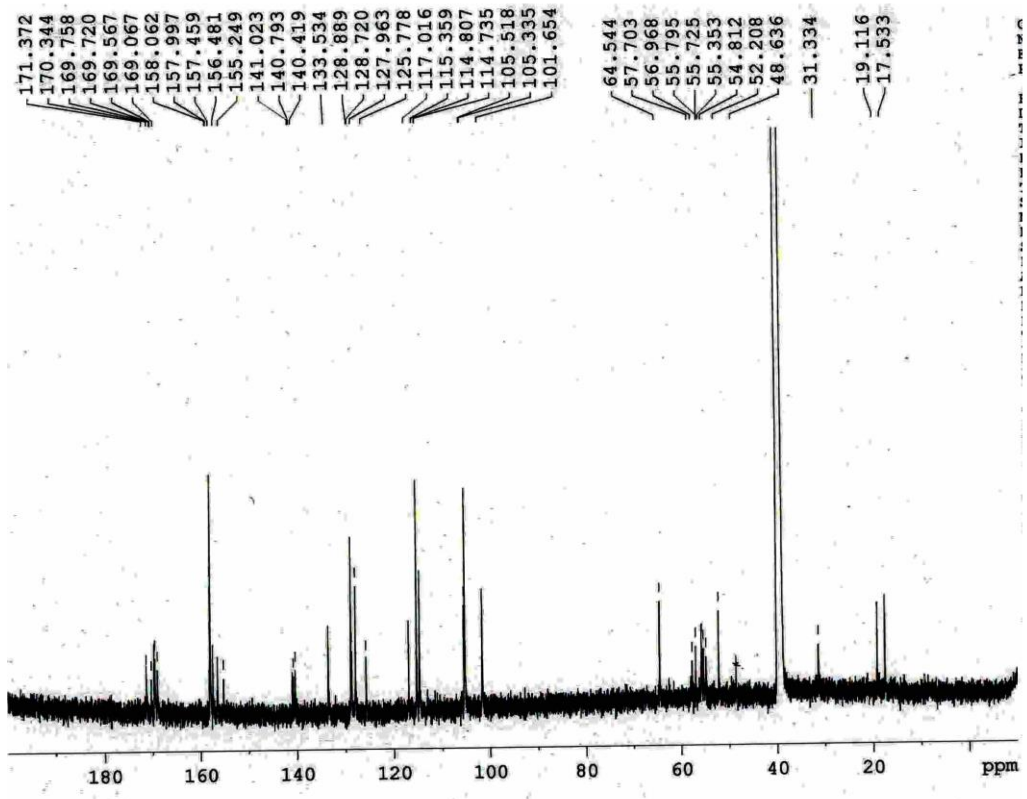

Supplementary Figure 40. ^{1}H NMR spectrum of Cbz-D-Hpg-D-Dpg-L-Val-D-Dpg-L-Hpg-D-Dpg-L-Hpg-OMe (**3**)

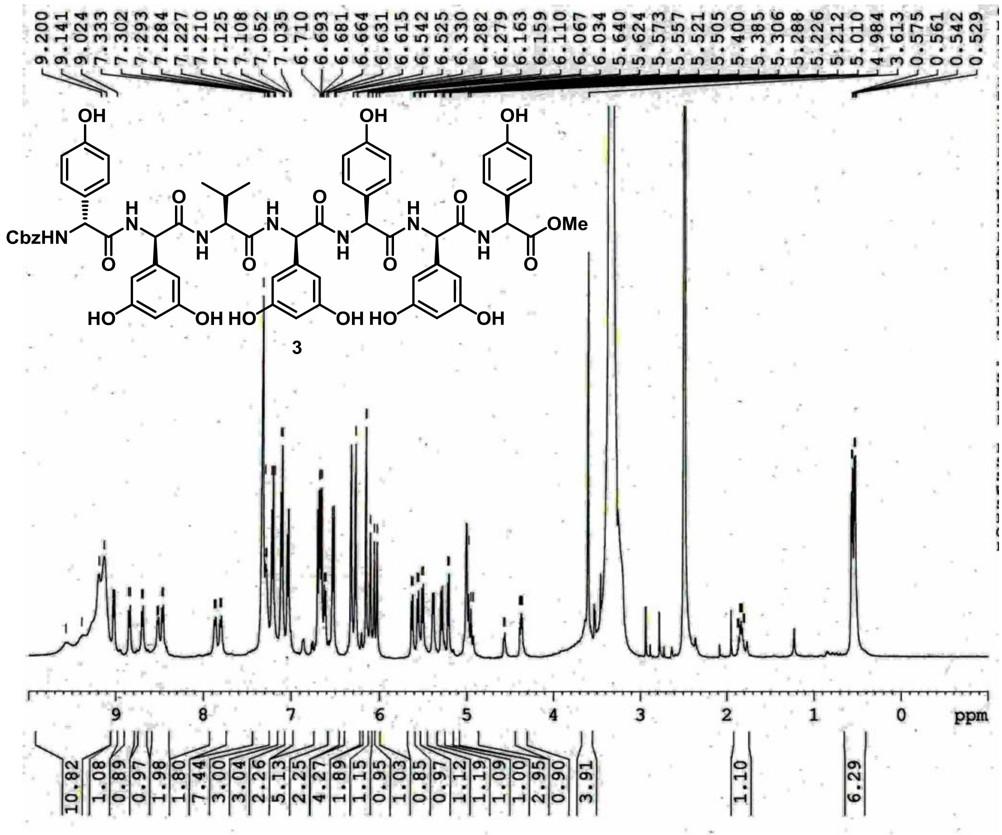

Supplementary Figure 41. ^{13}C NMR spectrum of Cbz-D-Hpg-D-Dpg-L-Val-D-Dpg-L-Hpg-D-Dpg-L-Hpg-OMe (**3**)

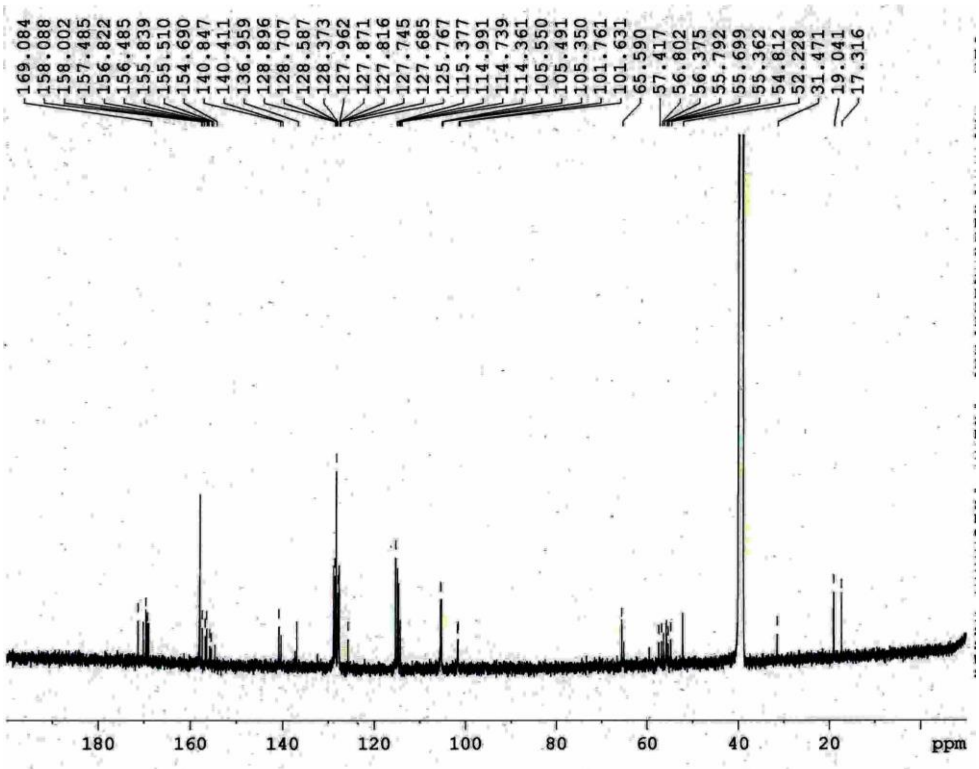

Supplementary Figure 42. ^{1}H NMR spectrum of Cbz-D-Hpg-D-Dpg-L-Val-D-Dpg-L-Hpg-D-Dpg-L-Hpg-D-Dpg-L-Val-D-Dpg-L-Hpg-L-Phe-L-Asp(*O*-Bn)-OBn (**38**)

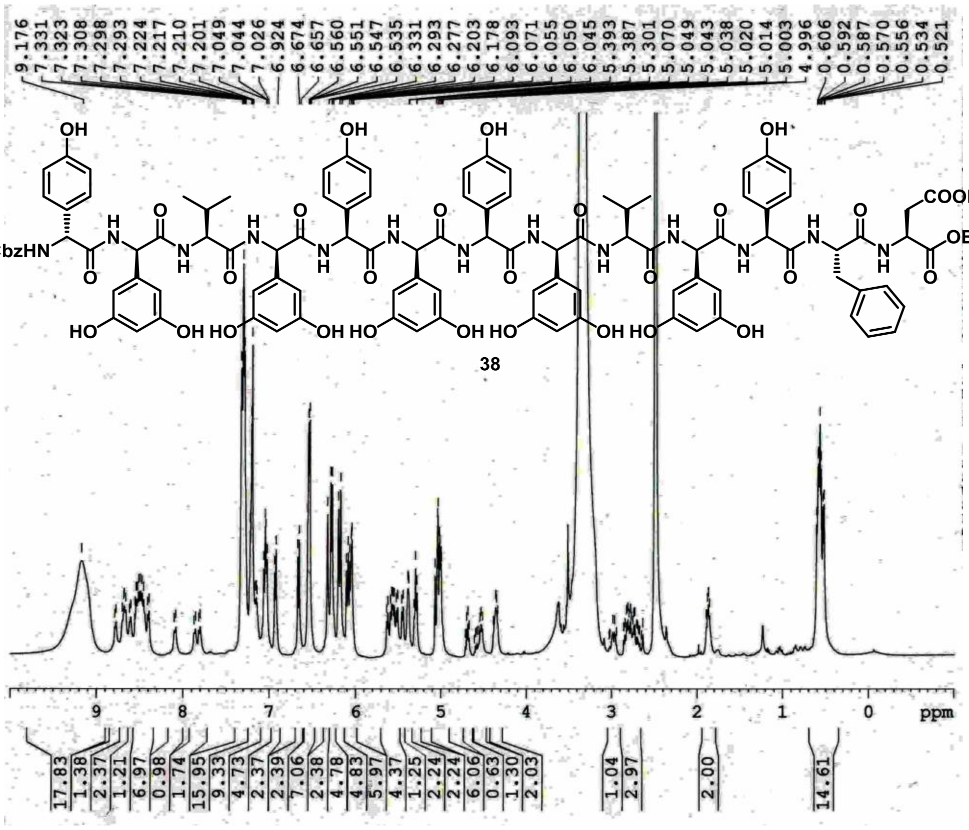

Supplementary Figure 43. ^{13}C NMR spectrum of

Cbz-D-Hpg-D-Dpg-L-Val-D-Dpg-L-Hpg-D-Dpg-L-Hpg-D-Dpg-L-Val-D-Dpg-L-Hpg-L-Phe-L-Asp(*O*-Bn)-OBn (**38**)

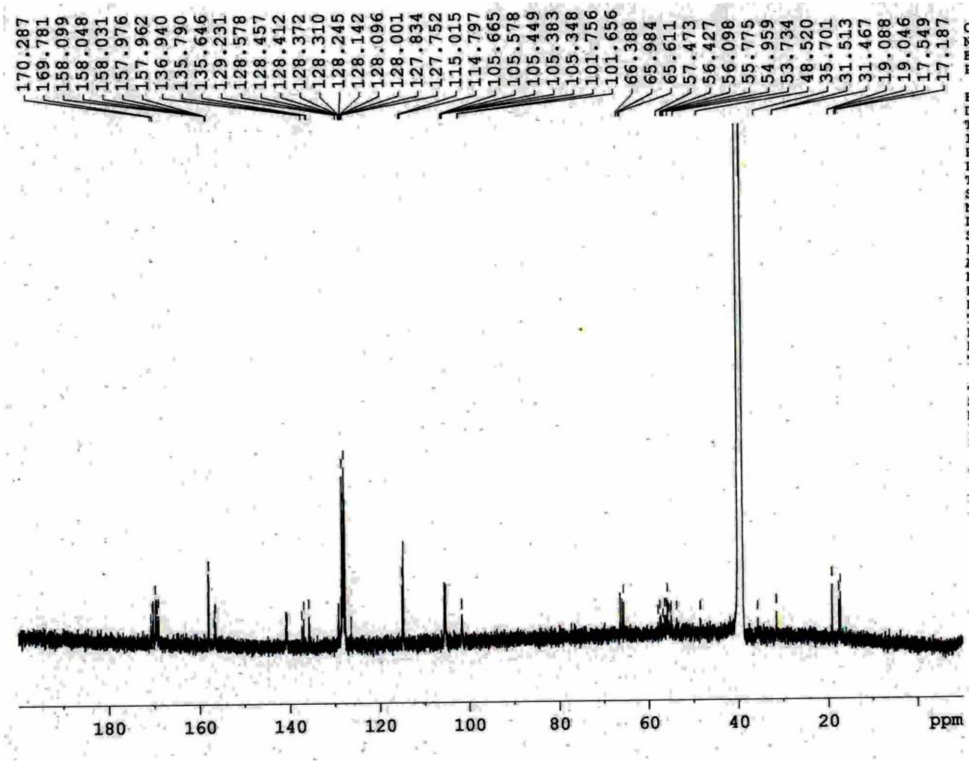

Supplementary Figure 44. ^{1}H NMR spectrum of feglymycin (**1**)

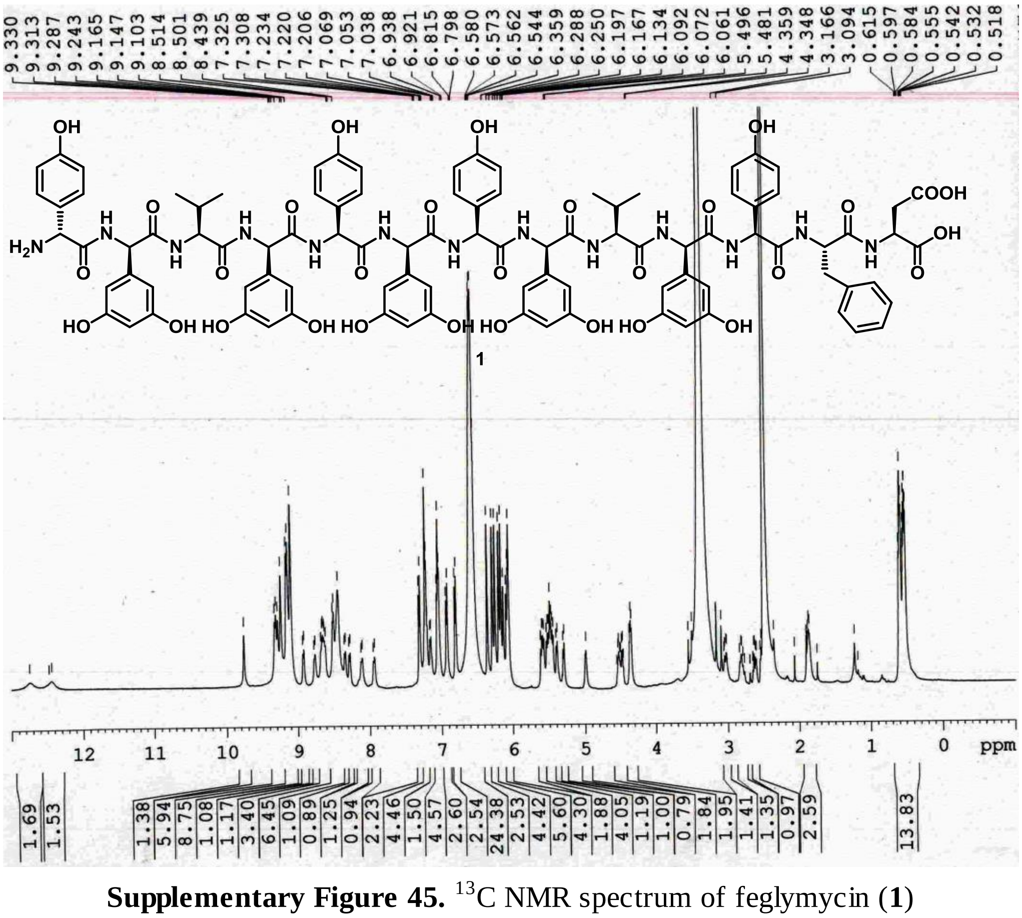

Supplementary Figure 45. ^{13}C NMR spectrum of feglymycin (**1**)

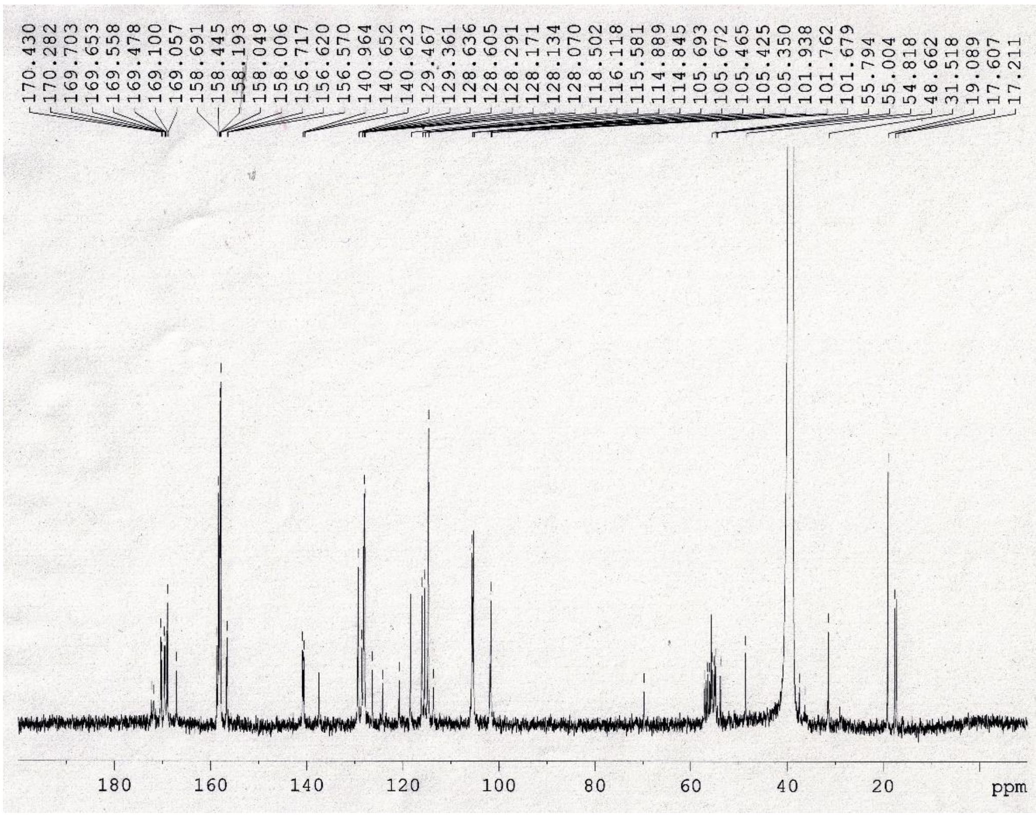

Supplementary Methods

1. Micro-flow reactor setup

Stainless steel T-shaped mixers were purchased from Sanko Seiki Co. Ltd. (inner diameter: 0.25 mm). Teflon® tube with inner diameter 0.8 mm was purchased from Senshu Scientific Co. Ltd. The T-shaped mixers and Teflon® tube were connected with PEEK fittings purchased from GL Science Co. Ltd. Solutions were introduced to micro-flow system with the syringe pumps (Harvard Pump 11 and Harvard PHD ULTRA) equipped gastight syringes (SGE). The gastight syringes and Teflon® tube were connected with joints purchased from Techno Applications Co. Ltd.

The employed micro-flow system is shown in Figure E-1. The gastight syringes and T-shaped mixers 1 and 2 were connected with Teflon® tube (inner diameter: 0.8 mm, length: 800 mm, volume 402 μL). T-shaped mixers 1 and 2 were connected with reaction tube 1 (Teflon® tube). T-shaped mixer 2 was connected with reaction tube 2 (Teflon® tube). The reaction tube 2 and Teflon® tube (inner diameter: 0.25 mm, length 100 mm, volume 5 μL) were connected to generate back pressure. T-shaped mixers and reaction tube were immersed in water bath.

2. General procedure for micro-flow amide bond formation of Hpgs

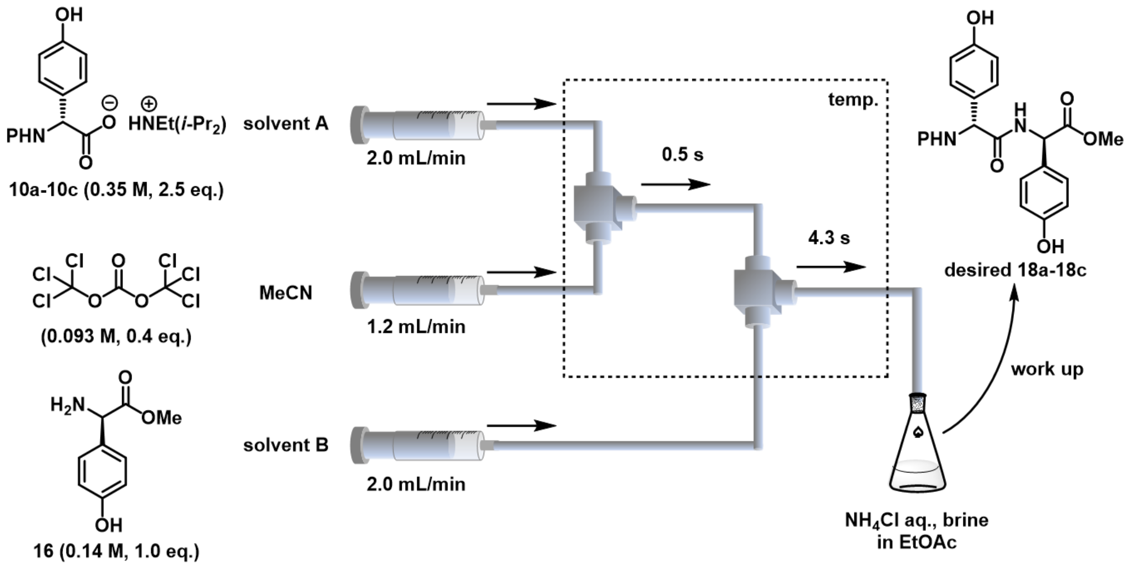

A solution of **protected D-Hpg 10a-10c** (0.35 M, 2.5 equiv.) and DIEA (0.35 M, 2.5 equiv.) in **solvent A** (flow rate: 2.0 mL/min) and a solution of triphosgene (0.093 M, 0.4 equiv.) in MeCN (flow rate: 1.2 mL/min) were introduced to T-shaped mixer 1 (inner diameter: 0.25 mm) at **temp.** with the syringe pumps. The resulting mixture was passed through reaction tube 1 (inner diameter: 0.8 mm, length: 54 mm, volume: 27 μL, reaction time: 0.5 s) at the same temperature. Then, the resulting mixture and a solution of H-D-Hpg-OMe (**16**) (0.14 M, 1.0 equiv.) in **solvent B** (flow rate: 2.0 mL/min) were introduced to T-shaped mixer 2 (inner diameter: 0.25 mm) at **temp.**. The resulting mixture was passed through reaction tube 2 (inner diameter: 0.8 mm, length: 742 mm, volume: 373 μL, reaction time: 4.3 s) at the same temperature. After being eluted for 20-40 s to reach a steady state, the resulting mixture was poured into saturated aqueous NH_4Cl, brine and EtOAc suspension for 30 s at room temperature. The aqueous layer was acidified with 1 M HCl and extracted three times with EtOAc. The combined organic layer was washed with 1 M HCl, twice with saturated aqueous $NaHCO_3$ and with brine, dried over Na_2SO_4, filtered and concentrated in vacuo. The residue was purified by column chromatography on silica gel (7% MeOH in $CHCl_3$) to give a mixture of **desired product 18a-18c** and **epimer 19a-19c**. Yield was determined by HPLC-UV analysis (conditions: DAICEL CHIRALPAK ID 4.6 mm × 25 cm, 20% or 25% EtOH in hexane, flow rate 1 mL/min, detection wavelength 277 nm).

Boc-D-Hpg-D-Hpg-OMe (18a)

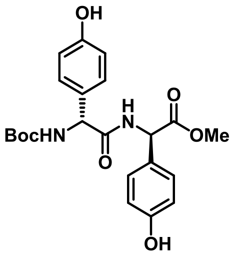

HPLC condition: 20% EtOH in hexane; retention time: 10.39 min

Colorless solid; ^{1}H NMR (400 MHz, DMSO-d$_6$): δ 9.56 (s, 1H), 9.37 (s, 1H), 8.72 (d, *J* = 6.4 Hz, 1H), 7.19 (d, *J* = 8.8 Hz, 2H), 7.16 (d, *J* = 8.8 Hz, 2H), 7.12 (d, *J* = 9.2 Hz, 1H), 6.75 (d, *J* = 8.8 Hz, 2H), 6.69 (d, *J* = 8.4

Hz, 2H), 5.25 (d, J = 6.4 Hz, 1H), 5.21 (d, J = 8.4 Hz, 1H), 3.54 (s, 3H), 1.37 (s, 9H); ^{13}C NMR (125 MHz, DMSO-d$_6$): δ 171.1, 170.4, 157.5, 156.7, 154.8, 129.1, 128.9, 128.4, 126.1, 115.4, 114.9, 78.3, 56.6, 55.8, 52.0, 28.2; IR (neat): 3295, 1724, 1638, 1611, 1510, 1490, 1433, 1210, 1162, 1025, 982, 775, 748 cm^{-1}; $[\alpha]^{30}_{D}$ = -114.0 (c 0.190, MeOH); mp 157-159 °C; HRMS (ESI-TOF): calcd for $[C_{22}H_{26}N_2O_7 + Na]^+$ 453.1632, found 453.1623.

Cbz-D-Hpg-D-Hpg-OMe (18b)

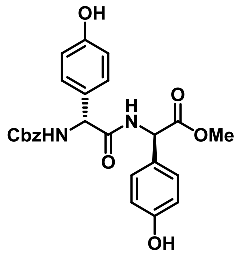

HPLC condition: 25% EtOH in hexane; retention time: 14.49 min

Colorless solid; ^{1}H NMR (500 MHz, DMSO-d$_6$): δ 9.57 (brs, 1H), 9.39 (brs, 1H), 8.80 (d, J = 7.0 Hz, 1H), 7.75 (d, J = 9.0 Hz, 1H), 7.35-7.30 (m, 5H), 7.22 (d, J = 8.5 Hz, 2H), 7.16 (d, J = 8.5 Hz, 2H), 6.75 (d, J = 8.5 Hz, 2H), 6.70 (d, J = 8.5 Hz, 2H), 5.30 (d, J = 9.0 Hz, 1H), 5.25 (d, J = 6.5 Hz, 1H), 5.01 (s, 2H), 3.53 (s, 3H); ^{13}C NMR (100 MHz, DMSO-d$_6$): δ 171.1, 170.3, 157.6, 156.9, 155.7, 137.0, 129.1, 128.6, 128.4, 127.9, 127.8, 126.2, 115.5, 115.0, 65.6, 57.1, 56.0, 52.1; IR (neat): 3303, 1731, 1673, 1627, 1511, 1439, 1266, 1211, 1170, 812, 773, 730 cm^{-1}; $[\alpha]^{29}_{D}$ = -105.4 (c 0.220, MeOH); mp 213-216 °C; HRMS (ESI-TOF): calcd for $[C_{25}H_{24}N_2O_7 + Na]^+$ 487.1476, found 487.1468.

Alloc-D-Hpg-D-Hpg-OMe (18c)

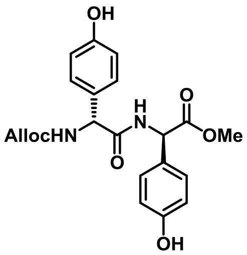

HPLC condition: 25% EtOH in hexane; retention time: 11.18 min

Colorless solid; ^{1}H NMR (500 MHz, DMSO-d$_6$): δ 9.58 (brs, 1H), 9.42 (brs, 1H), 8.01 (d, J = 6.5 Hz, 1H), 7.69 (d, J = 8.5 Hz, 1H), 7.23 (d, J = 8.5 Hz, 2H), 7.17 (d, J = 8.5 Hz, 2H), 6.77 (d, J = 8.5 Hz, 2H), 6.72 (d, J = 8.5 Hz, 2H), 5.93-5.85 (m, 1H), 5.30 (d, J = 9.0 Hz, 1H), 5.29 (d, J = 15.5 Hz, 1H), 5.27 (d, J = 6.5 Hz, 1H), 5.16 (d, J = 10.0 Hz, 1H), 4.47 (d, J = 5.5 Hz, 2H), 3.54 (s, 3H); ^{13}C NMR (125 MHz, DMSO-d$_6$): δ 171.1, 170.3, 157.6, 156.9, 155.4, 133.6, 129.2, 128.6, 128.6, 126.1, 117.1, 115.4, 114.9, 64.6, 57.0, 55.9, 52.1; IR (KBr): 3345, 1733, 1672, 1631, 1516, 1271, 1215, 1096, 960, 815 cm^{-1}; $[\alpha]^{30}_{D}$ = -122.6 (c 0.206, MeOH); mp 208-210 °C; HRMS (ESI-TOF): calcd for $[C_{21}H_{22}N_2O_7 + Na]^+$ 437.1319, found 437.1315.

3. Preparation of epimers

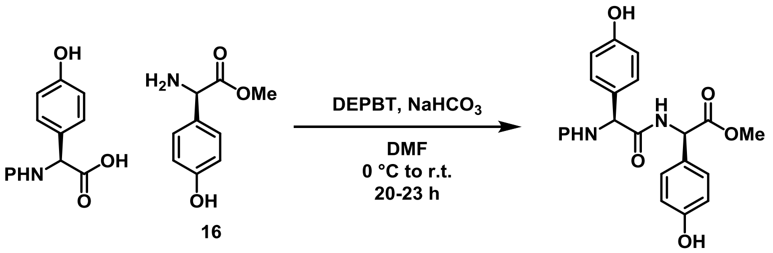

19a-19c

To a solution of H-D-Hpg-OMe (**16**) (0.20-0.23 mmol, 1.0 equiv.) and **protected L-Hpg** (0.20-0.23 mmol, 1.0 equiv.) in DMF (2.0-2.3 mL), $NaHCO_3$ (1.0 equiv.) and DEPBT (2.0 equiv.) was added at 0 °C under argon. After being stirred at same temperature for 1 h, the reaction mixture was warmed to room temperature. After being stirred at same temperature for 20-23 h, the reaction mixture was quenched with water. The aqueous layer was extracted three times with EtOAc. The combined organic layer was washed with water, saturated aqueous $NaHCO_3$ and brine, dried over $MgSO_4$, filtered and concentrated in vacuo. The residue was purified under **conditions** to give **product 19a-19c**.

Boc-L-Hpg-D-Hpg-OMe (19a)

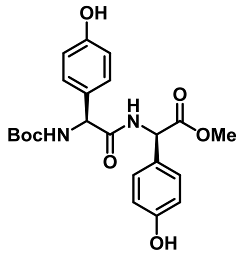

Purification conditions: column chromatography on silica gel (7% MeOH in $CHCl_3$)

100 mg, 0.230 mmol, quant.

HPLC condition: 20% EtOH in hexane; retention time: 18.51 min

Colorless solid; ^{1}H NMR (400 MHz, DMSO-d_6): δ 9.54 (s, 1H), 9.36 (s, 1H), 8.70 (d, *J* = 6.8 Hz, 1H), 7.18 (d, *J* = 8.4 Hz, 2H), 7.10-7.08 (m, 3H), 6.70 (d, *J* = 8.4 Hz, 2H), 6.65 (d, *J* = 8.4 Hz, 2H), 5.22 (d, *J* = 11.6 Hz, 1H), 5.21 (d, *J* = 6.8 Hz, 1H), 3.61 (s, 3H), 1.37 (s, 9H); ^{13}C NMR (125 MHz, DMSO-d_6): δ 171.3, 170.5, 157.5, 156.8, 154.7, 129.2, 128.9, 128.4, 125.9, 115.3, 114.9, 78.4, 56.7, 55.8, 52.1, 28.2; IR (neat): 3297, 1733, 1681, 1612, 1596, 1511, 1441, 1365, 1254, 1215, 1160, 835, 815, 747 cm^{-1}; $[\alpha]^{32}_D$ = -20.5 (c 0.216, MeOH); mp 110-113 °C; HRMS (ESI-TOF): calcd for $[C_{22}H_{26}N_2O_7 + Na]^+$ 453.1632, found 453.1630.

Cbz-L-Hpg-D-Hpg-OMe (19b)

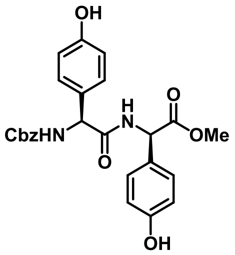

Purification conditions: preparative TLC (13% MeOH in CH_2Cl_2), Rf = 0.70

80.2 mg, 0.173 mmol, 87%

HPLC condition: 25% EtOH in hexane; retention time: 21.57 min

Colorless solid; ^{1}H NMR (500 MHz, DMSO-d_6): δ 9.56 (brs, 1H), 9.46 (brs, 1H), 8.83 (d, *J* = 7.0 Hz, 1H), 7.78 (d, *J* = 9.0 Hz, 1H), 7.36-7.28 (m, 5H), 7.22 (d, *J* = 8.5 Hz, 2H), 7.11 (d, *J* = 8.0 Hz, 2H), 6.72 (d, *J* = 8.5 Hz, 2H), 6.68 (d, *J* = 9.0 Hz, 2H), 5.37 (d, *J* = 9.0 Hz, 1H), 5.25 (d, *J* = 7.0 Hz, 1H), 5.06 (d, *J* = 12.5 Hz, 1H), 5.03 (d, *J* = 12.5 Hz, 1H), 3.61 (s, 3H); ^{13}C NMR (125 MHz, DMSO-d_6): δ 171.3, 170.4, 157.5, 156.9, 155.5, 137.0, 129.0, 128.9, 128.5, 128.4, 127.9, 127.7, 126.0, 115.4, 115.0, 65.6, 57.2, 55.8, 52.2; IR (neat): 3305, 1734, 1681, 1646, 1533, 1513, 1254, 1216, 984, 701 cm^{-1}; $[\alpha]^{30}_{D}$ = -26.2 (c 0.156, MeOH); mp 177-179 °C; HRMS (ESI-TOF): calcd for $[C_{25}H_{24}N_2O_7 + Na]^+$ 487.1476, found 487.1466.

Alloc-L-Hpg-D-Hpg-OMe (19c)

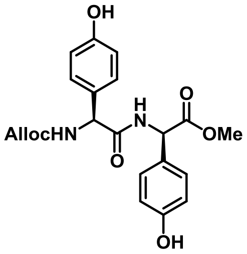

Purification conditions: preparative TLC (13% MeOH in CH_2Cl_2), Rf = 0.70

50.5 mg, 0.122 mmol, 61%

HPLC condition: 25% EtOH in hexane; retention time: 18.65 min

Colorless amorphous; ^{1}H NMR (500 MHz, DMSO-d_6): δ 9.54 (brs, 1H), 9.44 (brs, 1H), 8.80 (d, *J* = 6.5 Hz, 1H), 7.69 (d, *J* = 8.5 Hz, 1H), 7.19 (d, *J* = 7.5 Hz, 2H), 7.09 (d, *J* = 7.5 Hz, 2H), 6.71 (d, *J* = 7.0 Hz, 2H), 6.67 (d, *J* = 7.0 Hz, 2H), 5.93-5.83 (m, 1H), 5.32 (d, *J* = 9.5 Hz, 1H), 5.29 (d, *J* = 18.0 Hz, 1H), 5.22 (d, *J* = 7.0 Hz, 1H), 5.15 (d, *J* = 10.5 Hz, 1H), 3.61 (s, 3H); ^{13}C NMR (125 MHz, DMSO-d_6): δ 171.3, 170.4, 157.5, 156.9, 155.3, 133.6, 129.0, 128.9, 128.4, 125.9, 117.1, 115.4, 114.9, 64.6, 57.1, 55.8, 52.2; IR (KBr): 3297, 1655, 1613, 1512, 1439, 1219, 1173, 1046, 749 cm^{-1}; $[\alpha]^{30}_{D}$ = -24.9 (c 0.222, MeOH); HRMS (ESI-TOF): calcd for $[C_{21}H_{22}N_2O_7 + Na]^+$ 437.1319, found 437.1316.

4. Synthesis of C-terminal hexapeptide 2

Alloc-L-Phe-L-Asp(*O*-Bn)-OBn (20)

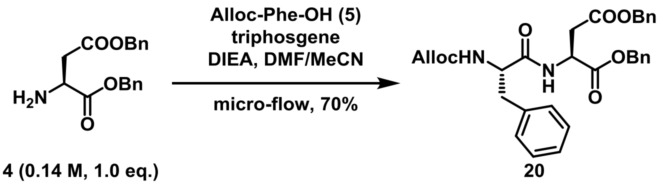

A solution of Alloc-L-Phe-OH (**5**) (0.35 M, 2.5 equiv.) and DIEA (0.42 M, 3.0 equiv.) in DMF (flow rate: 2.0 mL/min) and a solution of triphosgene (0.093 M, 0.4 equiv.) in MeCN (flow rate: 1.2 mL/min) were introduced to T-shaped mixer 1 at 20 °C with the syringe pumps. The resultant mixture was passed through reaction tube 1 (inner diameter: 0.8 mm, length: 54 mm, volume: 27 μL, reaction time: 0.5 s) at the same temperature. Then, the resultant mixture and a solution of H-L-Asp(*O*-Bn)-OBn (**4**) (0.14 M, 1.0 equiv.) in MeCN (flow rate: 2.0 mL/min) were introduced to T-shaped mixer 2 at 20 °C. The resultant mixture was passed through reaction tube 2 (inner diameter: 0.8 mm, length: 742 mm, volume: 373 μL, reaction time: 4.3 s) at the same temperature. After being eluted for *ca.* 20 s to reach a steady state, the resultant mixture was poured into saturated aqueous NH_4Cl and EtOAc suspension for 2 h 10 min at room temperature. The resultant suspension was acidified with 1 M HCl. The aqueous layer was extracted twice with EtOAc. The combined organic layer was washed twice with 3 M HCl, with brine, twice with saturated aqueous $NaHCO_3$ and with brine, dried over Na_2SO_4, filtered and concentrated in vacuo. The residue was recrystallized from EtOAc/hexane to give Alloc-L-Phe-L-Asp(*O*-Bn)-OBn (**20**) (13.9 g, 25.5 mmol, 70%) as a colorless solid. The combined aqueous layer was acidified with 6 M HCl at 0 °C and extracted twice with EtOAc. The organic layer was washed with brine, dried over $MgSO_4$, filtered and concentrated in vacuo. The residue was recrystallized with dicyclohexylamine from Et_2O. The obtained colorless solid was dissolved in EtOAc and desalinated by washing with 3 M HCl. After removing insoluble HCl salt of dicyclohexylamine by filtering through a pad of Celite, the resultant organic layer was concentrated in vacuo to give Alloc-L-Phe-OH (**5**) (10.3 g, 41.2 mmol, 63% based on unreacted **5**) as a colorless oil.

^1H NMR (400 MHz, $CDCl_3$): δ 7.35-7.13 (m, 15H), 7.00 (d, *J* = 8.3 Hz, 1H), 5.89-5.75 (m, 1H), 5.36 (brd, *J* = 7.3 Hz, 1H), 5.22 (d, *J* = 17.6 Hz, 1H), 5.15 (d, *J* = 10.8 Hz, 1H), 5.10 (s, 2H), 5.04 (d, *J* = 12.2 Hz, 1H), 4.99 (d, *J* = 12.2 Hz, 1H), 4.85 (ddd, *J* = 4.9, 4.9, 8.3 Hz, 1H), 4.48 (br, 3H), 3.08 (dd, *J* = 5.8, 14.2 Hz, 1H), 3.01 (dd, *J* = 4.9, 17.1 Hz, 1H), 2.99 (br, 1H), 2.87 (dd, *J* = 4.9, 17.1 Hz, 1H); ^{13}C NMR (100 MHz, $CDCl_3$): δ 170.8, 170.3, 169.9, 155.6, 136.1, 135.2, 135.0, 132.5, 129.2, 128.5, 67.5, 66.7, 65.7, 55.7, 48.7, 38.3, 36.1; IR (KBr): 3302, 3065, 3033, 2952, 1730, 1693, 1653, 1541, 1286, 1246, 1171, 702, 731, 547 cm^{-1}; $[\alpha]^{17}_D$ = +8.99 (c 1.07, $CHCl_3$); mp 111-114 °C; HRMS (ESI-TOF): calcd for $[C_{31}H_{32}N_2O_7+Na]^+$ 567.2102, found 567.2090.

Alloc-L-Hpg-L-Phe-L-Asp(*O*-Bn)-OBn (21)

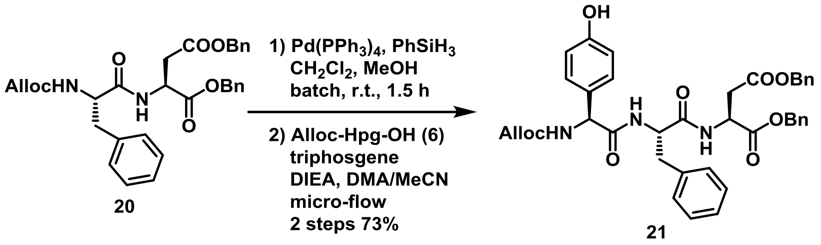

A solution of Alloc-L-Phe-L-Asp(*O*-Bn)-OBn (**20**) (17.2 g, 31.6 mmol, 1.0 equiv.) in CH_2Cl_2 (253 mL) and MeOH (63 mL) was degassed with argon. Phenylsilane (7.77 mL, 63.2 mmol, 2.0 equiv.) and tetrakis(triphenylphosphine)palladium (1.83 g, 1.58 mmol, 0.050 equiv.) were added at room temperature under argon. After being stirred at the same temperature for 1.5 h, the reaction mixture was concentrated in vacuo. The residue was purified by short path column chromatography on silica gel (4% MeOH in $CHCl_3$) and used for the next reaction without further purification.

A solution of Alloc-L-Hpg-OH (**6**) (0.35 M, 2.5 equiv.) and DIEA (0.35 M, 2.5 equiv.) in DMA (flow rate: 2.0 mL/min) and a solution of triphosgene (0.093 M, 0.4 equiv.) in MeCN (flow rate: 1.2 mL/min) were introduced to T-shaped mixer 1 at 10 °C with the syringe pumps. The resultant mixture was passed through reaction tube 1 (inner diameter: 0.8 mm, length: 106 mm, volume: 53 μL, reaction time: 1.0 s) at the same temperature. Then, the resultant mixture and a solution of amine **11** (0.14 M, 1.0 equiv.) in DMA (flow rate: 2.0 mL/min) were introduced to T-shaped mixer 2 at 10 °C. The resultant mixture was passed through reaction tube 2 (inner diameter: 0.8 mm, length: 742 mm, volume: 373 μL, reaction time: 4.3 s) at the same temperature. After being eluted for *ca.* 20 s to reach a steady state, the resultant mixture was poured into saturated aqueous NH_4Cl and EtOAc suspension for 113 min at room temperature. The resultant suspension was acidified with 3 M HCl. The aqueous layer was extracted twice with EtOAc. The combined organic layer was washed twice with 3 M HCl, with brine, twice with saturated aqueous $NaHCO_3$ and with brine, dried over Na_2SO_4, filtered and concentrated in vacuo. The residue was recrystallized from EtOAc/hexane to give Alloc-L-Hpg-L-Phe-L-Asp(*O*-Bn)-OBn (**21**) (16.1 g, 23.2 mmol, 2 steps 73%) as a colorless solid.

^{1}H NMR (400 MHz, DMSO-d$_6$): δ 9.36 (s, 1H), 8.60 (d, *J* = 8.3 Hz, 1H), 8.20 (d, *J* = 8.8 Hz, 1H), 7.59 (d, *J* = 8.3 Hz, 1H), 7.35-7.30 (m, 10H), 7.19-7.16 (m, 5H), 7.03 (d, *J* = 8.8 Hz, 2H), 6.62 (d, *J* = 8.3 Hz, 2H), 5.93-5.82 (m, 1H), 5.28 (d, *J* = 17.6 Hz, 1H), 5.16 (d, *J* = 10.2 Hz, 1H), 5.11-5.04 (m, 5H), 4.74 (ddd, *J* = 6.8, 6.8, 7.8 Hz, 1H), 4.59 (ddd, *J* = 4.4, 9.3, 9.3 Hz, 1H), 4.46 (d, *J* = 4.9 Hz, 2H), 2.98 (dd, *J* = 4.4, 14.2 Hz, 1H), 2.88 (dd, *J* = 6.8, 17.1 Hz, 1H), 2.79-2.73 (m, 2H); ^{13}C NMR (100 MHz, DMSO-d$_6$): δ 170.8, 170.2, 169.8, 169.6, 156.7, 155.3, 137.4, 135.7, 135.6, 133.4, 129.2, 128.5, 128.4, 128.3, 128.0, 127.9, 127.7, 126.2, 117.0, 114.8, 66.3, 65.9, 64.5, 57.8, 53.4, 48.5, 37.6, 35.6; IR (KBr): 3412, 3314, 3065, 3038, 2940, 2929, 1739, 1679, 1659, 1637, 1522, 1367, 1220, 985, 931, 813, 748, 697, 677 cm^{-1}; $[\alpha]^{21}_D$ = +13.1 (c 0.283, MeOH); mp 170-172 °C; HRMS (ESI-TOF): calcd for $[C_{39}H_{39}N_3O_9 + Na]^+$ 716.2579, found 716.2563.

Alloc-D-Dpg-L-Hpg-L-Phe-L-Asp(*O*-Bn)-OBn (23)

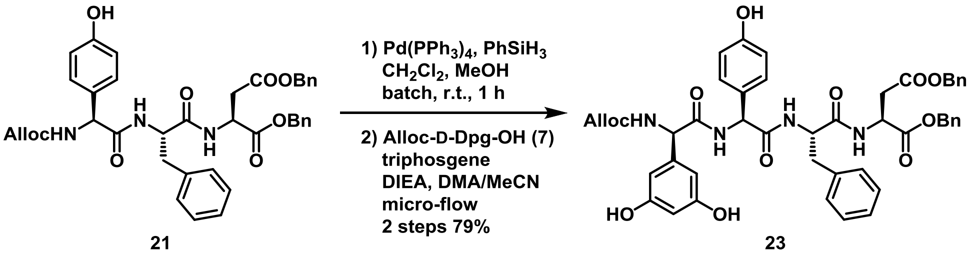

A solution of Alloc-L-Hpg-L-Phe-L-Asp(*O*-Bn)-OBn (**21**) (4.86 g, 7.01 mmol, 1.0 equiv.) in CH_2Cl_2 (56 mL) and MeOH (14 mL) was degassed with argon. Phenylsilane (1.72 mL, 14.0 mmol, 2.0 equiv.) and tetrakis(triphenylphosphine)palladium (404 mg, 0.35 mmol, 0.050 equiv.) were added at room temperature under argon. After being stirred at the same temperature for 1 h, SiliaMetS® Thiourea (1.08 mmol/g, 2.59 g, 2.80 mmol, 0.4 equiv.) was added at room temperature. After being stirred at the same temperature over 30 min, the reaction mixture was filtered and concentrated in vacuo. The residue was purified by short path column chromatography on silica gel (5% MeOH in $CHCl_3$) and used for the next reaction without further purification.

Alloc-D-Dpg-OH (**7**) was azeotroped twice with dry MeCN. A solution of Alloc-D-Dpg-OH (**7**) (0.35 M, 2.5 equiv.) and DIEA (0.35 M, 2.5 equiv.) in DMA (flow rate: 2.0 mL/min) and a solution of triphosgene (0.093 M, 0.4 equiv.) in MeCN (flow rate: 1.2 mL/min) were introduced to T-shaped mixer 1 at 10 °C with the syringe pumps. The resultant mixture was passed through reaction tube 1 (inner diameter: 0.8 mm, length: 106 mm, volume: 53 μL, reaction time: 1.0 s) at the same temperature. Then, the resultant mixture and a solution of amine **22** (0.14 M, 1.0 equiv.) in DMA (flow rate: 2.0 mL/min) were introduced to T-shaped mixer 2 at 10 °C. The resultant mixture was passed through reaction tube 2 (inner diameter: 0.8 mm, length: 742 mm, volume: 373 μL, reaction time: 4.3 s) at the same temperature. After being eluted for *ca.* 20 s to reach a steady state, the resultant mixture was poured into saturated aqueous NH_4Cl and EtOAc suspension for 25 min at room temperature. The resultant suspension was acidified with 1 M HCl. The aqueous layer was extracted three times with EtOAc. The combined organic layer was washed twice with 3 M HCl, with brine, twice with saturated aqueous $NaHCO_3$ and with brine, dried over Na_2SO_4, filtered and concentrated in vacuo. The residue was recrystallized from EtOH/hexane to give Alloc-D-Dpg-L-Hpg-L-Phe-L-Asp(*O*-Bn)-OBn (**23**) (4.76 g, 5.54 mmol, 2 steps 79%) as a colorless solid.

^{1}H NMR (500 MHz, DMSO-d_6): δ 9.39 (s, 1H), 9.24 (s, 2H), 8.67 (d, *J* = 7.0 Hz, 1H), 8.43 (d, *J* = 8.0 Hz, 1H), 8.30 (d, *J* = 8.0 Hz, 1H), 7.75 (d, *J* = 7.5 Hz, 1H), 7.35-7.30 (m, 10H), 7.25-7.20 (m, 5H), 6.90 (d, *J* = 8.0 Hz, 2H), 6.58 (d, *J* = 8.0 Hz, 2H), 6.33 (s, 2H), 6.16 (s, 1H), 5.95-5.86 (m, 1H), 5.32 (d, *J* = 17.5 Hz, 1H), 5.26-5.20 (m, 2H), 5.17 (d, *J* = 10.5 Hz, 1H), 5.11-5.06 (m, 4H), 4.76 (ddd, *J* = 6.5, 7.0, 7.0 Hz, 1H), 4.58 (ddd, *J* = 4.0, 9.0, 9.0 Hz, 1H), 4.50 (br, 2H), 3.11 (br, 1H), 2.92 (dd, *J* = 7.5, 17.0 Hz, 1H), 2.86-2.76 (m, 2H); ^{13}C NMR (125 MHz, DMSO-d_6): δ 170.9, 170.3, 170.2, 169.8, 169.7, 158.2, 156.9, 155.8, 139.7, 137.7, 135.9, 135.7, 133.4, 129.3, 128.6, 128.5, 128.5, 128.2, 128.1, 128.1, 128.1, 127.9, 126.3, 117.2, 114.9, 105.8, 102.0, 66.5, 66.0, 64.8, 57.9, 56.4, 53.8, 48.7, 37.4, 35.8; IR (KBr): 3278, 3065, 2944, 1734, 1707, 1672, 1639, 1602, 1514, 1455, 1389, 1335, 1250, 1164, 1049, 1005, 970, 839, 750, 699, 589, 527 cm^{-1}; $[\alpha]^{22}_D$ = -4.05 (c 0.200, MeOH); mp 146-149 °C; HRMS (ESI-TOF): calcd for $[C_{47}H_{46}N_4O_{12}+Na]^+$ 881.3004, found 881.3018.

Alloc-L-Val-D-Dpg-L-Hpg-L-Phe-L-Asp(*O*-Bn)-OBn (25)

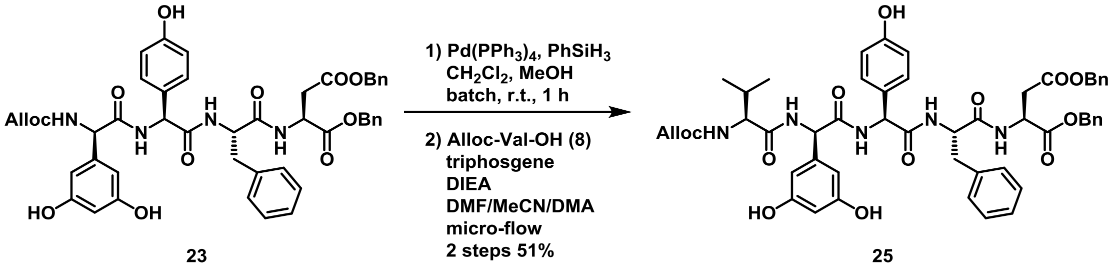

A solution of Alloc-D-Dpg-L-Hpg-L-Phe-L-Asp(*O*-Bn)-OBn (**23**) (1.20 g, 1.40 mmol, 1.0 equiv.) in CH_2Cl_2 (11.2 mL) and MeOH (2.8 mL) was degassed with argon. Phenylsilane (344 μL, 2.80 mmol, 2.0 equiv.) and tetrakis(triphenylphosphine)palladium (81 mg, 0.0700 mmol, 0.050 equiv.) were added at room temperature under argon. After being stirred at the same temperature for 1 h, SiliaMetS® Thiourea (1.08 mmol/g, 519 mg, 0.560 mmol, 0.4 equiv.) was added at room temperature. After being stirred at the same temperature over 30 min, the reaction mixture was filtered and concentrated in vacuo. The residue was purified by short path column chromatography on silica gel (1.5% H_2O, 13.5% MeOH in $CHCl_3$) and used for the next reaction without further purification.

Alloc-L-Val-OH (**8**) was azeotroped twice with dry MeCN. A solution of Alloc-L-Val-OH (**8**) (0.35 M, 2.5 equiv.) and DIEA (0.42 M, 3.0 equiv.) in DMF (flow rate: 2.0 mL/min) and a solution of triphosgene (0.093 M, 0.4 equiv.) in MeCN (flow rate: 1.2 mL/min) were introduced to T-shaped mixer 1 at 20 °C with the syringe pumps. The resultant mixture was passed through reaction tube 1 (inner diameter: 0.8 mm, length: 54 mm, volume: 27 μL, reaction time: 0.5 s) at the same temperature. Then, the resultant mixture and a solution of amine **24** (0.14 M, 1.0 equiv.) in DMA (flow rate: 2.0 mL/min) were introduced to T-shaped mixer 2 at 20 °C. The resultant mixture was passed through reaction tube 2 (inner diameter: 0.8 mm, length: 742 mm, volume: 373 μL, reaction time: 4.3 s) at the same temperature. After being eluted for *ca.* 20 s to reach a steady state, the resultant mixture was poured into saturated aqueous NH_4Cl and EtOAc suspension for 4 min 40 s at room temperature. The resultant suspension was acidified with 1 M HCl. The aqueous layer was extracted three times with EtOAc. The combined organic layer was washed twice with 3 M HCl, with brine, twice with saturated aqueous $NaHCO_3$ and with brine, dried over Na_2SO_4, filtered and concentrated in vacuo. The residue was recrystallized from EtOH/Et_2O/hexane to give Alloc-L-Val-D-Dpg-L-Hpg-L-Phe-L-Asp(*O*-Bn)-OBn (**25**) (639 mg, 0.667 mmol, 2 steps 51%) as a colorless solid.

^{1}H NMR (400 MHz, DMSO-d$_6$): δ 9.31 (s, 1H), 9.18 (s, 2H), 8.61 (d, *J* = 7.8 Hz, 1H), 8.44 (d, *J* = 7.8 Hz, 1H), 8.43 (d, *J* = 6.8 Hz, 1H), 8.33 (d, *J* = 8.3 Hz, 1H), 7.35-7.30 (m, 10H), 7.23-7.16 (m, 6H), 6.89 (d, *J* = 8.8 Hz, 2H), 6.54 (d, *J* = 8.8 Hz, 2H), 6.29 (d, *J* = 2.0 Hz, 2H), 6.11 (t, *J* = 2.0 Hz, 1H), 5.94-5.84 (m, 1H), 5.40 (d, *J* = 6.8 Hz, 1H), 5.28 (d, *J* = 16.1 Hz, 1H), 5.25 (d, *J* = 7.3 Hz, 1H), 5.14 (d, *J* = 10.8 Hz, 1H), 5.09-5.02 (m, 4H), 4.74 (ddd, *J* = 6.8, 6.8, 7.8 Hz, 1H), 4.54 (ddd, *J* = 4.4, 9.3, 9.3 Hz, 1H), 4.50-4.39 (m, 2H), 4.10 (dd, *J* = 6.8, 9.3 Hz, 1H), 3.03 (dd, *J* = 4.4, 14.2 Hz, 1H), 2.91-2.76 (m, 3H), 2.03-1.91 (m, 1H), 0.81 (d, *J* = 5.4 Hz, 3H), 0.79 (d, *J* = 6.4 Hz, 3H); ^{13}C NMR (100 MHz, DMSO-d$_6$): δ 171.2, 170.7, 170.3, 169.8, 169.7, 169.6, 158.1, 156.7, 155.9, 139.9, 137.6, 135.8, 135.7, 129.2, 128.4, 128.3, 128.1, 128.0, 127.9, 127.7, 126.2, 117.0,

114.8, 105.7, 101.8, 66.3, 65.9, 64.5, 59.5, 56.1, 55.9, 53.7, 48.5, 37.3, 35.6, 30.7, 19.2, 17.8; IR (KBr): 3275, 3069, 3032, 2971, 1727, 1690, 1630, 1509, 1456, 1381, 1342, 1286, 1241, 1159, 1047, 996, 842, 747, 697, 588, 526, 489 cm^{-1}; $[\alpha]^{22}_{D}$ = -8.15 (c 0.200, MeOH); mp 213-216 °C; HRMS (ESI-TOF): calcd for $[C_{52}H_{55}N_5O_{13}+Na]^+$ 980.3689, found 980.3688.

Alloc-D-Dpg-L-Val-D-Dpg-L-Hpg-L-Phe-L-Asp(*O*-Bn)-OBn (2)

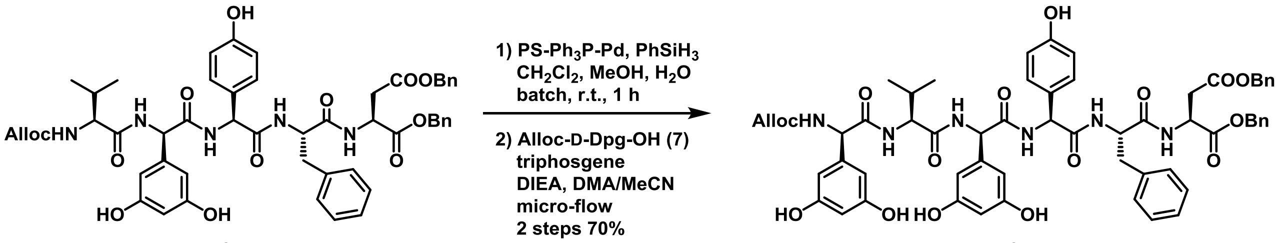

25 2

A solution of Alloc-L-Val-D-Dpg-L-Hpg-L-Phe-L-Asp(*O*-Bn)-OBn (**25**) (870 mg, 0.908 mmol, 1.0 equiv.) in CH_2Cl_2 (7.3 mL), MeOH (5.4 mL) and H_2O (0.9 mL) was degassed with argon. Phenylsilane (224 μL, 1.82 mmol, 2.0 equiv.) and tetrakis(triphenylphosphine)palladium, polymer-bound (0.07 mmol/g, 240 mg, 0.0168 mmol, 0.019 equiv.) were added at room temperature under argon. After being stirred at the same temperature for 1 h, the reaction mixture was filtered through a pad of Celite and concentrated in vacuo. The residue was azeotroped with MeOH/toluene and used for the next reaction without further purification.

Alloc-D-Dpg-OH (**7**) was azeotroped twice with dry MeCN. A solution of Alloc-D-Dpg-OH (**7**) (0.35 M, 2.5 equiv.) and DIEA (0.35 M, 2.5 equiv.) in DMA (flow rate: 2.0 mL/min) and a solution of triphosgene (0.093 M, 0.4 equiv.) in MeCN (flow rate: 1.2 mL/min) were introduced to T-shaped mixer 1 at 10 °C with the syringe pumps. The resultant mixture was passed through reaction tube 1 (inner diameter: 0.8 mm, length: 106 mm, volume: 53 μL, reaction time: 1.0 s) at the same temperature. Then, the resultant mixture and a solution of amine **26** (0.14 M, 1.0 equiv.) in DMA (flow rate: 2.0 mL/min) were introduced to T-shaped mixer 2 at 10 °C. The resultant mixture was passed through reaction tube 2 (inner diameter: 0.8 mm, length: 742 mm, volume: 373 μL, reaction time: 4.3 s) at the same temperature. After being eluted for *ca.* 20 s to reach a steady state, the resultant mixture was poured into saturated aqueous NH_4Cl and EtOAc suspension for 3 min at room temperature. The resultant suspension was acidified with 1 M HCl that was saturated with NaCl. The aqueous layer was extracted three times with EtOAc. The combined organic layer was washed twice with 3 M HCl that was saturated with NaCl, twice with saturated aqueous $NaHCO_3$ and with brine, dried over Na_2SO_4, filtered and concentrated in vacuo. The residue was recrystallized from EtOH/hexane to give Alloc-D-Dpg-L-Val-D-Dpg-L-Hpg-L-Phe-L-Asp(*O*-Bn)-OBn (**2**) (656 mg, 0.584 mmol, 2 steps 70%) as a colorless solid.

^{1}H NMR (400 MHz, DMSO-d$_6$): δ 9.28 (s, 1H), 9.14 (s, 2H), 9.13 (s, 2H), 8.58 (d, *J* = 7.3 Hz, 1H), 8.53 (d, *J* = 8.8 Hz, 1H), 8.50 (d, *J* = 8.3 Hz, 1H), 8.39 (d, *J* = 8.8 Hz, 1H), 8.06 (d, *J* = 9.8 Hz, 1H), 7.42 (d, *J* = 8.3 Hz, 1H), 7.35-7.38 (m, 10H), 7.22-7.14 (m, 5H), 6.92 (d, *J* = 7.8 Hz, 2H), 6.54 (d, *J* = 8.8 Hz, 2H), 6.33 (s, 2H), 6.29 (s, 2H), 6.09 (s, 2H), 5.91-5.77 (m, 1H), 5.48 (d, *J* = 7.8 Hz, 1H), 5.31 (d, *J* = 8.3 Hz, 1H), 5.27 (d, *J* = 17.6 Hz, 1H), 5.26 (d, *J* = 8.8 Hz, 1H), 5.12 (d, *J* = 9.8 Hz, 1H), 5.09-5.01 (m, 4H), 4.74 (ddd, *J* = 6.4, 6.8,

6.8 Hz, 1H), 4.55 (ddd, J = 4.9, 8.8, 9.3 Hz, 1H), 4.44-4.42 (m, 3H), 3.00 (dd, J = 4.4, 13.6 Hz, 1H), 2.91-2.76 (m, 3H), 1.98-1.84 (m, 1H), 0.63 (d, J = 6.4 Hz, 6H); ^{13}C NMR (100 MHz, DMSO-d$_6$): δ 170.7, 170.4, 170.3, 169.8, 169.6, 169.3, 158.0, 158.0, 156.6, 155.1, 141.1, 140.4, 137.4, 135.8, 135.6, 133.5, 129.2, 128.4, 128.3, 128.3, 128.2, 128.0, 127.9, 127.8, 126.3, 116.9, 114.7, 105.6, 105.3, 101.7, 66.3, 65.9, 64.5, 57.6, 56.9, 55.8, 55.6, 53.6, 48.5, 35.7, 31.4, 19.0, 17.4; IR (KBr): 3264, 3064, 2965, 1729, 1704, 1691, 1649, 1631, 1601, 1507, 1385, 1265, 1157, 1105, 1058, 1050, 1004, 840, 701 cm^{-1}; $[\alpha]^{23}_{D}$ = -21.9 (c 0.205, MeOH); mp 257-259 °C; HRMS (ESI-TOF): calcd for $[C_{60}H_{62}N_6O_{16} + Na]^+$ 1145.4115, found 1145.4113.

5. Synthesis of N-terminal heptapeptide 3

Alloc-D-Dpg-L-Hpg-OMe (27)

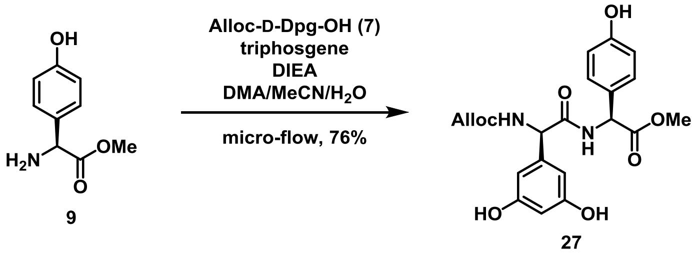

Alloc-D-Dpg-OH (**7**) was azeotroped twice with dry MeCN. A solution of Alloc-D-Dpg-OH (**7**) (0.35 M, 2.5 equiv.) and DIEA (0.35 M, 2.5 equiv.) in DMA (flow rate: 2.0 mL/min) and a solution of triphosgene (0.093 M, 0.4 equiv.) in MeCN (flow rate: 1.2 mL/min) were introduced to T-shaped mixer 1 at 10 °C with the syringe pumps. The resultant mixture was passed through reaction tube 1 (inner diameter: 0.8 mm, length: 54 mm, volume: 27 μL, reaction time: 0.5 s) at the same temperature. Then, the resultant mixture and a solution of H-L-Hpg-OMe (**9**) (0.14 M, 1.0 equiv.) in H_2O/MeCN (1/2, flow rate: 2.0 mL/min) were introduced to T-shaped mixer 2 at 10 °C. The resultant mixture was passed through reaction tube 2 (inner diameter: 0.8 mm, length: 742 mm, volume: 373 μL, reaction time: 4.3 s) at the same temperature. After being eluted for *ca.* 20 s to reach a steady state, the resultant mixture was poured into saturated aqueous NH_4Cl and EtOAc suspension for 30 s at room temperature. The resultant suspension was acidified with 1 M HCl. The aqueous layer was extracted three times with EtOAc. The combined organic layer was washed twice with 3 M HCl, twice with saturated aqueous $NaHCO_3$ and with brine, dried over Na_2SO_4, filtered and concentrated in vacuo. The residue was purified by column chromatography on silica gel (10% MeOH in $CHCl_3$) to give Alloc-D-Dpg-L-Hpg-OMe (**27**) (46.4 mg, 0.108 mmol, 76%, epimer 1%) as a colorless amorphous. The ratio of desired product and epimer was determined by ^{1}H NMR analysis.

^{1}H NMR (400 MHz, DMSO-d$_6$): δ 9.65-9.05 (br, 3H), 8.83 (d, J = 6.8 Hz, 1H), 7.63 (d, J = 8.8 Hz, 1H), 7.12 (d, J = 8.8 Hz, 2H), 6.72 (d, J = 8.8 Hz, 2H), 6.25 (d, J = 2.0 Hz, 2H), 6.09 (t, J = 2.0 Hz, 1H), 5.94-5.84 (m, 1H), 5.30 (d, J = 17.2 Hz, 1H), 5.25 (d, J = 8.8 Hz, 1H), 5.20 (d, J = 7.2 Hz, 1H), 5.16 (d, J = 8.8 Hz, 1H), 4.49-4.46 (m, 2H), 3.61 (s, 3H); ^{13}C NMR (125 MHz, DMSO-d$_6$): δ 171.3, 169.8, 158.1, 157.5, 155.2, 140.5, 133.5, 129.0, 125.7, 117.1, 115.4, 105.3, 101.7, 64.6, 57.4, 55.9, 52.1; IR (neat): 3327, 1731, 1698, 1667, 1606, 1516, 1456, 1339, 1261, 1232, 1167, 1010 cm^{-1}; $[\alpha]^{30}_{D}$ = +29.9 (c 0.228, MeOH); HRMS (ESI-TOF): calcd for $[C_{21}H_{22}N_2O_8 + Na]^+$ 453.1268, found 453.1274.

Alloc-L-Hpg-D-Dpg-L-Hpg-OMe (28)

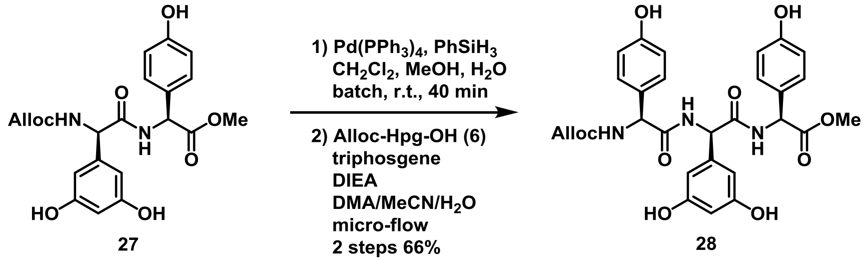

A solution of Alloc-D-Dpg-L-Hpg-OMe (**27**) (143 mg, 0.332 mmol, 1.0 equiv.) in CH_2Cl_2 (2.6 mL), MeOH (0.66 mL) and H_2O (0.07 mL) was degassed with argon. Phenylsilane (81 μL, 0.664 mmol, 2.0 equiv.) and tetrakis(triphenylphosphine)palladium (7.0 mg, 0.0061 mmol, 0.018 equiv.) were added at room temperature under argon. After being stirred at the same temperature for 40 min, SiliaMetS® Thiourea (1.08 mmol/g, 122 mg, 0.132 mmol, 0.40 equiv.) was added at room temperature. After being stirred at the same temperature over 30 min, the reaction mixture was filtered and concentrated in vacuo. The residue was purified by short path column chromatography on silica gel (18% MeOH in CH_2Cl_2) and used for the next reaction without further purification.

A solution of Alloc-L-Hpg-OH (**6**) (0.35 M, 2.5 equiv.) and DIEA (0.35 M, 2.5 equiv.) in DMA (flow rate: 2.0 mL/min) and a solution of triphosgene (0.093 M, 0.40 equiv.) in MeCN (flow rate: 1.2 mL/min) were introduced to T-shaped mixer 1 at 10 °C with the syringe pumps. The resultant mixture was passed through reaction tube 1 (inner diameter: 0.8 mm, length: 54 mm, volume: 27 μL, reaction time: 0.5 s) at the same temperature. Then, the resultant mixture and a solution of amine **14** (0.14 M, 1.0 equiv.) in H_2O/MeCN (1/2, flow rate: 2.0 mL/min) were introduced to T-shaped mixer 2 at 10 °C. The resultant mixture was passed through reaction tube 2 (inner diameter: 0.8 mm, length: 742 mm, volume: 373 μL, reaction time: 4.3 s) at the same temperature. After being eluted for *ca.* 20 s to reach a steady state, the resultant mixture was poured into saturated aqueous NH_4Cl and EtOAc suspension for 44 s at 10 °C. The resultant suspension was acidified with 1 M HCl that was saturated with NaCl. The aqueous layer was extracted three times with EtOAc. The combined organic layer was washed twice with 3 M HCl that was saturated with NaCl, with brine, twice with saturated aqueous $NaHCO_3$ and with brine, dried over Na_2SO_4, filtered and concentrated in vacuo. The residue was purified by column chromatography on silica gel (10% MeOH in CH_2Cl_2) to give Alloc-L-Hpg-D-Dpg-L-Hpg-OMe (**28**) (83.6 mg, 0.144 mmol, 2 steps 66%) as a colorless amorphous.

^{1}H NMR (400 MHz, DMSO-d_6): δ 9.52 (s, 1H), 9.34 (s, 1H), 9.12 (s, 2H), 8.90 (d, *J* = 6.8 Hz, 1H), 8.53 (d, *J* = 8.4 Hz, 1H), 7.71 (d, *J* = 8.4 Hz, 1H), 7.18 (d, *J* = 8.4 Hz, 2H), 7.12 (d, *J* = 8.4 Hz, 2H), 6.70 (d, *J* = 8.8 Hz, 2H), 6.65 (d, *J* = 8.8 Hz, 2H), 6.16 (d, *J* = 2.0 Hz, 2H), 6.06 (t, *J* = 2.0 Hz, 1H), 5.93-5.84 (m, 1H), 5.43 (d, *J* = 7.2 Hz, 1H), 5.32 (d, *J* = 8.4 Hz, 1H), 5.29 (d, *J* = 17.6 Hz, 1H), 5.22 (d, *J* = 7.2 Hz, 1H), 5.16 (d, *J* = 10.0 Hz, 1H), 4.45 (d, *J* = 5.2 Hz, 2H), 3.62 (s, 3H); ^{13}C NMR (125 MHz, DMSO-d_6): δ 171.2, 169.8, 169.7, 158.1, 157.5, 156.8, 155.4, 140.1, 133.5, 128.9, 128.7, 128.5, 125.7, 117.1, 115.3, 114.9, 105.4, 101.7, 64.6, 57.2, 55.9, 55.6, 52.2; IR (neat): 3309, 1697, 1660, 1652, 1613, 1515, 1455, 1339, 1231, 1173, 839 cm^{-1}; $[\alpha]^{29}_D$ = +53.0 (c 0.190, MeOH); HRMS (ESI-TOF): calcd for $[C_{29}H_{29}N_3O_{10}+Na]^+$ 602.1745, found 602.1736.

Alloc-D-Dpg-L-Hpg-D-Dpg-L-Hpg-OMe (30)

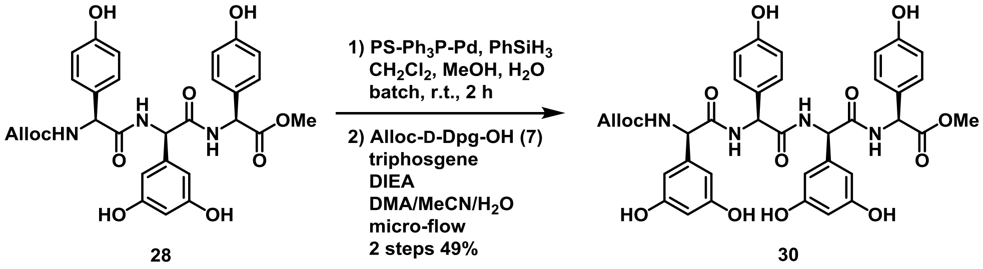

A solution of Alloc-L-Hpg-D-Dpg-L-Hpg-OMe (**28**) (432 mg, 0.745 mmol, 1.0 equiv.) in CH_2Cl_2 (6.0 mL), MeOH (1.5 mL) and H_2O (0.15 mL) was degassed with argon. Phenylsilane (183 μL, 1.49 mmol, 2.0 equiv.) and tetrakis(triphenylphosphine)palladium, polymer-bound (0.07 mmol/g, 213 mg, 0.0149 mmol, 0.020 equiv.) were added at room temperature under argon. After being stirred at the same temperature for 2 h, the reaction mixture was filtered and concentrated in vacuo. The residue was purified by short path column chromatography on silica gel (2% H_2O, 18% MeOH in CH_2Cl_2) and used for the next reaction without further purification.

Alloc-D-Dpg-OH (**7**) was azeotroped twice with dry MeCN. A solution of Alloc-D-Dpg-OH (**7**) (0.35 M, 2.5 equiv.) and DIEA (0.35 M, 2.5 equiv.) in DMA (flow rate: 2.0 mL/min) and a solution of triphosgene (0.093 M, 0.40 equiv.) in MeCN (flow rate: 1.2 mL/min) were introduced to T-shaped mixer 1 at 10 °C with the syringe pumps. The resultant mixture was passed through reaction tube 1 (inner diameter: 0.8 mm, length: 54 mm, volume: 27 μL, reaction time: 0.5 s) at the same temperature. Then, the resultant mixture and a solution of amine **29** (0.14 M, 1.0 equiv.) in H_2O/MeCN (1/2, flow rate: 2.0 mL/min) were introduced to T-shaped mixer 2 at 10 °C. The resultant mixture was passed through reaction tube 2 (inner diameter: 0.8 mm, length: 742 mm, volume: 373 μL, reaction time: 4.3 s) at the same temperature. After being eluted for *ca.* 20 s to reach a steady state, the resultant mixture was poured into saturated aqueous NH_4Cl and EtOAc suspension for 130 s at room temperature. The resultant suspension was acidified with 1 M HCl that was saturated with NaCl. The aqueous layer was extracted three times with EtOAc. The combined organic layer was washed twice with 3 M HCl that was saturated with NaCl, with brine, twice with saturated aqueous $NaHCO_3$ and with brine, dried over Na_2SO_4, filtered and concentrated in vacuo. The residue was purified by column chromatography on silica gel (12% MeOH in CH_2Cl_2) to give Alloc-D-Dpg-L-Hpg-D-Dpg-L-Hpg-OMe (**30**) (253 mg, 0.340 mmol, 2 steps 49%) as a colorless amorphous.

^{1}H NMR (500 MHz, DMSO-d_6): δ 9.65-9.05 (br, 6H), 8.97 (d, *J* = 7.0 Hz, 1H), 8.80 (d, *J* = 8.0 Hz, 1H), 8.59 (d, *J* = 8.0 Hz, 1H), 7.55 (d, *J* = 8.5 Hz, 1H), 7.12 (d, *J* = 8.5 Hz, 2H), 7.05 (d, *J* = 8.0 Hz, 2H), 6.70 (d, *J* = 8.5 Hz, 2H), 6.56 (d, *J* = 8.5 Hz, 2H), 6.28 (d, *J* = 2.0 Hz, 2H), 6.17 (d, *J* = 2.0 Hz, 2H), 6.09 (t, *J* = 2.0 Hz, 1H), 6.04 (t, *J* = 2.0 Hz, 1H), 5.93-5.85 (m, 1H), 5.59 (d, *J* = 8.0 Hz, 1H), 5.47 (d, *J* = 8.5 Hz, 1H), 5.30 (d, *J* = 20.0 Hz, 1H), 5.27 (d, *J* = 9.0 Hz, 1H), 5.21 (d, *J* = 7.0 Hz, 1H), 5.16 (d, *J* = 10.0 Hz, 1H), 4.48-4.45 (m, 2H), 3.61 (s, 3H); ^{13}C NMR (125 MHz, DMSO-d_6): δ 171.3, 169.7, 169.5, 169.2, 158.1, 158.0, 157.5, 156.6, 155.3, 140.6, 140.2, 133.6, 128.9, 128.7, 128.0, 125.8, 117.0, 115.4, 114.8, 105.4, 101.7, 64.6, 57.6, 55.9, 55.5, 55.0, 52.2; IR (neat): 3308, 1652, 1606, 1514, 1456, 1339, 1260, 1232, 1164, 1009, 839, 693 cm^{-1}; $[\alpha]^{29}_{D}$ =

+27.8 (c 0.316, MeOH); HRMS (ESI-TOF): calcd for $[C_{37}H_{36}N_4O_{13}+Na]^+$ 767.2171, found 767.2171.

Alloc-L-Val-D-Dpg-L-Hpg-D-Dpg-L-Hpg-OMe (32)

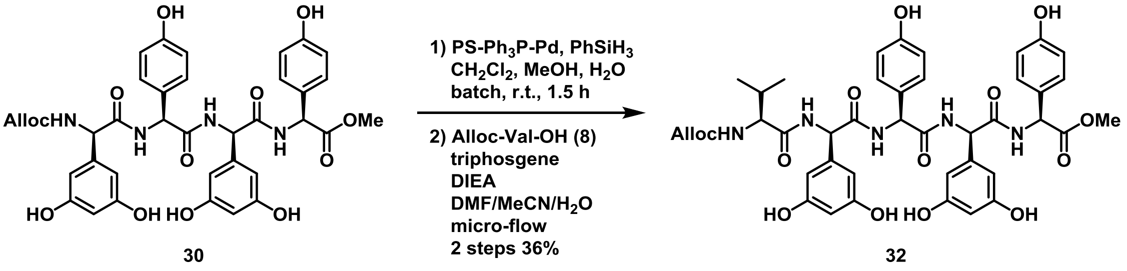

A solution of Alloc-D-Dpg-L-Hpg-D-Dpg-L-Hpg-OMe (**30**) (431 mg, 0.579 mmol, 1.0 equiv.) in CH_2Cl_2 (4.6 mL), MeOH (1.2 mL) and H_2O (0.10 mL) was degassed with argon. Phenylsilane (143 μL, 1.16 mmol, 2.0 equiv.) and tetrakis(triphenylphosphine)palladium, polymer-bound (0.07 mmol/g, 166 mg, 0.0116 mmol, 0.020 equiv.) were added at room temperature under argon. After being stirred at the same temperature for 1.5 h, the reaction mixture was filtered and concentrated in vacuo. The residue was purified by short path column chromatography on silica gel (3% H_2O, 27% MeOH in $CHCl_3$) and used for the next reaction without further purification.

Alloc-L-Val-OH (**8**) was azeotroped twice with dry MeCN. A solution of Alloc-L-Val-OH (**8**) (0.35 M, 2.5 equiv.) and DIEA (0.35 M, 2.5 equiv.) in DMF (flow rate: 2.0 mL/min) and a solution of triphosgene (0.093 M, 0.40 equiv.) in MeCN (flow rate: 1.2 mL/min) were introduced to T-shaped mixer 1 at 20 °C with the syringe pumps. The resultant mixture was passed through reaction tube 1 (inner diameter: 0.8 mm, length: 54 mm, volume: 27 μL, reaction time: 0.5 s) at the same temperature. Then, the resultant mixture and a solution of amine **31** (0.14 M, 1.0 equiv.) in H_2O/MeCN (1/2, flow rate: 2.0 mL/min) were introduced to T-shaped mixer 2 at 20 °C. The resultant mixture was passed through reaction tube 2 (inner diameter: 0.8 mm, length: 742 mm, volume: 373 μL, reaction time: 4.3 s) at the same temperature. After being eluted for *ca.* 20 s to reach a steady state, the resultant mixture was poured into saturated aqueous NH_4Cl and EtOAc suspension for 103 s at 20 °C. The resultant suspension was acidified with 1 M HCl that was saturated with NaCl. The aqueous layer was extracted three times with EtOAc. The combined organic layer was washed twice with 3 M HCl that was saturated with NaCl, with brine, twice with saturated aqueous $NaHCO_3$ and with brine, dried over Na_2SO_4, filtered and concentrated in vacuo. The residue was recrystallized from IPA/EtOAc/hexane to give Alloc-L-Val-D-Dpg-L-Hpg-D-Dpg-L-Hpg-OMe (**32**) (170 mg, 0.201 mmol, 2 steps 36%) as a colorless solid. The combined aqueous HCl layer was neutralized by saturated aqueous $NaHCO_3$ (pH 7.0), desalinated by reverse phase column chromatography (InertSep C18 10 g/60 mL, H_2O to MeOH) and purified by short path column chromatography on silica gel (3% H_2O, 27% MeOH in $CHCl_3$) to give amine **31** (98.4 mg, 0.148 mmol, 31% recovery) as a colorless solid.

^{1}H NMR (500 MHz, DMSO-d$_6$): δ 9.65-9.05 (br, 6H), 9.04 (d, *J* = 6.5 Hz, 1H), 8.84 (d, *J* = 7.5 Hz, 1H), 8.68 (d, *J* = 7.5 Hz, 1H), 8.31 (d, *J* = 7.5 Hz, 1H), 7.24 (d, *J* = 9.0 Hz, 1H), 7.12 (d, *J* = 7.5 Hz, 2H), 7.06 (d, *J* = 8.5 Hz, 2H), 6.70 (d, *J* = 7.0 Hz, 2H), 6.55 (d, *J* = 7.0 Hz, 2H), 6.28 (s, 2H), 6.16 (s, 2H), 6.09 (s, 1H), 6.03 (s, 1H), 5.95-5.86 (m, 1H), 5.63 (d, *J* = 7.5 Hz, 1H), 5.52 (d, *J* = 8.0 Hz, 2H), 5.29 (d, *J* = 17.5 Hz, 1H), 5.21

(d, J = 7.0 Hz, 1H), 5.17 (d, J = 10.0 Hz, 1H), 4.47 (br, 2H), 4.05 (dd, J = 7.0, 8.5 Hz, 1H), 3.61 (s, 3H), 2.00-1.90 (m, 1H), 0.80 (d, J = 7.0 Hz, 3H), 0.78 (d, J = 7.0 Hz, 3H); ^{13}C NMR (125 MHz, DMSO-d_6): δ 171.4, 170.8, 169.7, 169.6, 169.1, 158.1, 158.0, 157.5, 156.5, 156.0, 140.7, 140.4, 133.7, 128.9, 128.1, 128.0, 125.7, 117.0, 115.4, 114.8, 105.5, 105.3, 101.7, 64.5, 59.8, 55.9, 55.4, 55.0, 54.9, 52.2, 30.6, 19.3, 18.0; IR (KBr): 3296, 2966, 1650, 1611, 1342, 1261, 1231, 1164, 1009, 840 cm^{-1}; $[\alpha]^{29}_{D}$ = +10.4 (c 0.200, MeOH); mp 187-189 °C; HRMS (ESI-TOF): calcd for $[C_{42}H_{45}N_5O_{14}+Na]^+$ 866.2855, found 862.2842.

Alloc-D-Dpg-L-Val-D-Dpg-L-Hpg-D-Dpg-L-Hpg-OMe (34)

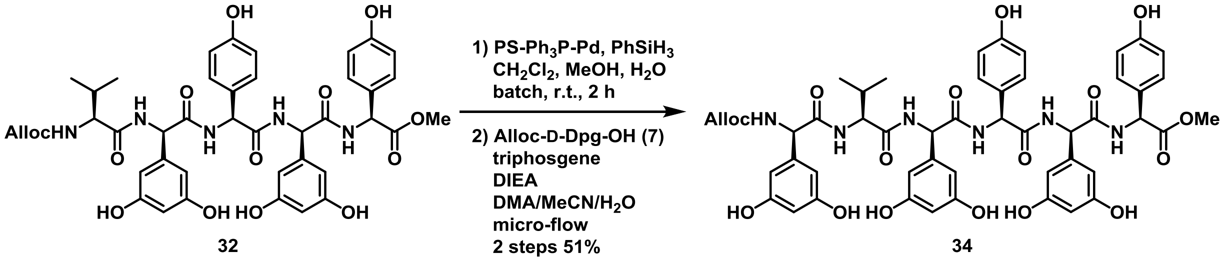

A solution of Alloc-L-Val-D-Dpg-L-Hpg-D-Dpg-L-Hpg-OMe (**32**) (129 mg, 0.153 mmol, 1.0 equiv.) in CH_2Cl_2 (1.8 mL), MeOH (0.6 mL) and H_2O (0.06 mL) was degassed with argon. Phenylsilane (38 μL, 0.306 mmol, 2.0 equiv.) and tetrakis(triphenylphosphine)palladium, polymer-bound (0.07 mmol/g, 44 mg, 0.00306 mmol, 0.020 equiv.) were added at room temperature under argon. After being stirred at the same temperature for 2 h, the reaction mixture was filtered and concentrated in vacuo. The residue was purified by short path column chromatography on silica gel (3% H_2O, 27% MeOH in $CHCl_3$) and used for the next reaction without further purification.

Alloc-D-Dpg-OH (**7**) was azeotroped twice with dry MeCN. A solution of Alloc-D-Dpg-OH (**7**) (0.35 M, 5.0 equiv.) and DIEA (0.35 M, 5.0 equiv.) in DMA (flow rate: 2.0 mL/min) and a solution of triphosgene (0.093 M, 0.8 equiv.) in MeCN (flow rate: 1.2 mL/min) were introduced to T-shaped mixer 1 at 10 °C with the syringe pumps. The resultant mixture was passed through reaction tube 1 (inner diameter: 0.8 mm, length: 54 mm, volume: 27 μL, reaction time: 0.5 s) at the same temperature. Then, the resultant mixture and a solution of amine **33** (0.070 M, 1.0 equiv.) in H_2O/MeCN (1/2, flow rate: 2.0 mL/min) were introduced to T-shaped mixer 2 at 10 °C. The resultant mixture was passed through reaction tube 2 (inner diameter: 0.8 mm, length: 742 mm, volume: 373 μL, reaction time: 4.3 s) at the same temperature. After being eluted for *ca.* 20 s to reach a steady state, the resultant mixture was poured into saturated aqueous NH_4Cl and EtOAc suspension for 60 s at 10 °C. The resultant suspension was acidified with 1 M HCl that was saturated with NaCl. The aqueous layer was extracted three times with EtOAc. The combined organic layer was washed twice with 3 M HCl that was saturated with NaCl, with brine, twice with saturated aqueous $NaHCO_3$ and with brine, dried over Na_2SO_4, filtered and concentrated in vacuo. The residue was recrystallized from IPA/hexane to give Alloc-D-Dpg-L-Val-D-Dpg-L-Hpg-D-Dpg-L-Hpg-OMe (**34**) (81.5 mg, 0.0808 mmol, 2 steps 51%) as a colorless solid. The combined aqueous HCl layer was neutralized by saturated aqueous $NaHCO_3$ (pH 7.0) and desalinated by reverse phase column chromatography (InertSep C18 10 g/60 mL, H_2O to MeOH) to give amine **33** (35.5 mg, 0.0467 mmol, 34% recovery) as a colorless solid.

^{1}H NMR (400 MHz, DMSO-d$_6$): δ 9.65-9.05 (br, 8H), 9.03 (d, *J* = 6.8 Hz, 1H), 8.84 (d, *J* = 8.0 Hz, 1H), 8.70 (d, *J* = 8.0 Hz, 1H), 8.48 (d, *J* = 8.0 Hz, 1H), 8.08 (d, *J* = 8.8 Hz, 1H), 7.53 (d, *J* = 8.4 Hz, 1H), 7.12 (d, *J* = 8.4 Hz, 2H), 7.05 (d, *J* = 8.4 Hz, 2H), 6.70 (d, *J* = 8.4 Hz, 2H), 6.54 (d, *J* = 8.4 Hz, 2H), 6.33 (s, 2H), 6.29 (s, 2H), 6.16 (s, 2H), 6.09 (s, 1H), 6.07 (s, 1H), 6.03 (s, 1H), 5.94-5.83 (m, 1H), 5.64 (d, *J* = 8.0 Hz, 1H), 5.57 (d, *J* = 8.0 Hz, 1H), 5.53 (d, *J* = 8.0 Hz, 1H), 5.30 (d, *J* = 17.6 Hz, 1H), 5.24 (d, *J* = 8.0 Hz, 1H), 5.22 (d, *J* = 7.2 Hz, 1H), 5.15 (d, *J* = 10.4 Hz, 1H), 4.45 (br, 2H), 4.40 (dd, *J* = 6.4, 8.4 Hz, 1H), 3.62 (s, 3H), 1.96-1.83 (m, 1H), 0.72-0.52 (m, 6H); ^{13}C NMR (125 MHz, DMSO-d$_6$): δ 171.4, 170.3, 169.8, 169.7, 169.6, 169.0, 158.1, 158.0, 157.5, 156.5, 155.2, 141.0, 140.8, 140.4, 133.5, 128.9, 128.7, 128.0, 125.8, 117.0, 115.4, 114.8, 114.7, 105.5, 105.3, 101.7, 64.5, 57.7, 57.0, 55.8, 55.7, 55.4, 54.8, 52.2, 31.3, 19.1, 17.5; IR (KBr): 3287, 2962, 2920, 2850, 1647, 1604, 1513, 1456, 1338, 1259, 1231, 1157, 1007, 840 cm^{-1}; $[\alpha]^{28}_{D}$ = -7.53 (c 0.222, MeOH); mp 198-200 °C; HRMS (ESI-TOF): calcd for $[C_{50}H_{52}N_6O_{17}+Na]^+$ 1031.3281, found 1031.3280.

Cbz-D-Hpg-D-Dpg-L-Val-D-Dpg-L-Hpg-D-Dpg-L-Hpg-OMe (3)

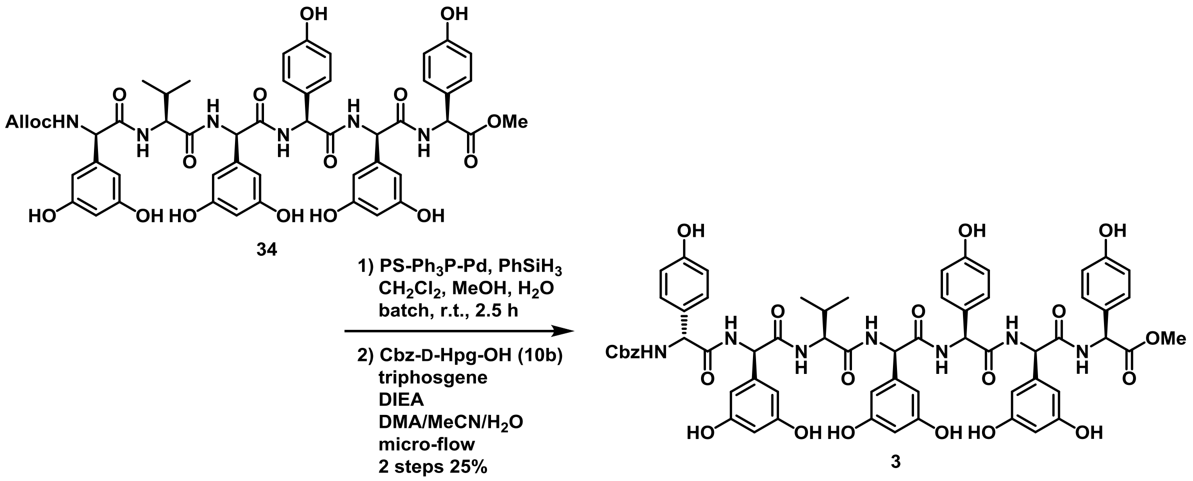

A solution of Alloc-D-Dpg-L-Val-D-Dpg-L-Hpg-D-Dpg-L-Hpg-OMe (**34**) (130 mg, 0.129 mmol, 1.0 equiv.) in CH_2Cl_2 (1.5 mL), MeOH (1.0 mL) and H_2O (0.1 mL) was degassed with argon. Phenylsilane (32 μL, 0.258 mmol, 2.0 equiv.) and tetrakis(triphenylphosphine)palladium, polymer-bound (0.07 mmol/g, 37 mg, 0.00258 mmol, 0.020 equiv.) were added at room temperature under argon. After being stirred at the same temperature for 2.5 h, the reaction mixture was filtered and concentrated in vacuo. The residue was purified by short path column chromatography on silica gel (3% H_2O, 27% MeOH in $CHCl_3$) and used for the next reaction without further purification.

A solution of Cbz-D-Hpg-OH (**10b**) (0.18 M, 5.0 equiv.) and DIEA (0.18 M, 5.0 equiv.) in DMA (flow rate: 2.0 mL/min) and a solution of triphosgene (0.047 M, 0.80 equiv.) in MeCN (flow rate: 1.2 mL/min) were introduced to T-shaped mixer 1 at 10 °C with the syringe pumps. The resultant mixture was passed through reaction tube 1 (inner diameter: 0.8 mm, length: 54 mm, volume: 27 μL, reaction time: 0.5 s) at the same temperature. Then, the resultant mixture and a solution of amine **35** (0.035 M, 1.0 equiv.) in H_2O/MeCN (1/2, flow rate: 2.0 mL/min) were introduced to T-shaped mixer 2 at 10 °C. The resultant mixture was passed through reaction tube 2 (inner diameter: 0.8 mm, length: 742 mm, volume: 373 μL, reaction time: 4.3 s) at the

same temperature. After being eluted for *ca.* 20 s to reach a steady state, the resultant mixture was poured into saturated aqueous NH_4Cl and EtOAc suspension for 48 s at 10 °C. The resultant suspension was acidified with 1 M HCl that was saturated with NaCl. The aqueous layer was extracted three times with EtOAc. The combined organic layer was washed twice with 3 M HCl that was saturated with NaCl, with brine, twice with saturated aqueous $NaHCO_3$ and with brine, dried over Na_2SO_4, filtered and concentrated in vacuo. The residue was purified by preparative TLC (25% MeOH in $CHCl_3$, Rf = 0.43) to give Cbz-D-Hpg-D-Dpg-L-Val-D-Dpg-L-Hpg-D-Dpg-L-Hpg-OMe (**3**) (25.7 mg, 0.0213 mmol, 2 steps 25%) as a colorless solid. The combined aqueous HCl layer was neutralized by saturated aqueous $NaHCO_3$ (pH 7.0) and desalinated by reverse phase column chromatography (InertSep C18 10 g/60 mL, H_2O to MeOH) to give amine **35** (12.6 mg, 0.0136 mmol, 24% recovery) as a colorless solid.

^{1}H NMR (500 MHz, DMSO-d$_6$): δ 9.65-9.05 (br, 9H), 9.03 (d, *J* = 7.0 Hz, 1H), 8.86 (d, *J* = 8.0 Hz, 1H), 8.70 (d, *J* = 8.0 Hz, 1H), 8.53 (d, *J* = 6.5 Hz, 1H), 8.47 (d, *J* = 7.5 Hz, 1H), 7.87 (d, *J* = 8.0 Hz, 1H), 7.81 (d, *J* = 8.5 Hz, 1H), 7.35-7.25 (m, 5H), 7.22 (d, *J* = 8.5 Hz, 2H), 7.12 (d, *J* = 8.5 Hz, 2H), 7.04 (d, *J* = 8.5 Hz, 2H), 6.70 (d, *J* = 8.5 Hz, 2H), 6.67 (d, *J* = 8.5 Hz, 2H), 6.53 (d, *J* = 8.5 Hz, 2H), 6.33 (s, 2H), 6.28 (s, 2H), 6.16 (s, 2H), 6.11 (s, 1H), 6.07 (s, 1H), 6.03 (s, 1H), 5.63 (d, *J* = 8.0 Hz, 1H), 5.57 (d, *J* = 8.0 Hz, 1H), 5.51 (d, *J* = 8.0 Hz, 1H), 5.39 (d, *J* = 7.5 Hz, 1H), 5.30 (d, *J* = 9.0 Hz, 1H), 5.22 (d, *J* = 7.0 Hz, 1H), 5.01 (brs, 2H), 4.38 (dd, *J* = 6.0, 9.0 Hz, 1H), 3.61 (s, 3H), 1.88-1.79 (m, 1H), 0.57 (d, *J* = 7.0 Hz, 3H), 0.54 (d, *J* = 6.5 Hz, 3H); ^{13}C NMR (125 MHz, DMSO-d$_6$): δ 171.4, 170.2, 169.7, 169.6, 169.4, 169.1, 158.1, 158.0, 157.5, 156.8, 156.5, 155.8, 155.5, 154.7, 140.8, 140.4, 137.0, 128.9, 128.7, 128.6, 128.4, 128.0, 127.9, 127.8, 127.7, 127.7, 125.8, 115.4, 115.0, 114.7, 114.4, 105.6, 105.5, 105.4, 101.8, 101.6, 65.6, 57.4, 56.8, 56.4, 55.8, 55.7, 55.4, 54.8, 52.2, 31.5, 19.0, 17.3; IR (KBr): 3298, 2964, 1636, 1614, 1513, 1455, 1339, 1261, 1227, 1155, 1055, 1007, 839 cm^{-1}; $[\alpha]^{27}_D$ = -29.1 (c 0.482, MeOH); mp 217-219 °C; HRMS (ESI-TOF): calcd for $[C_{62}H_{61}N_7O_{19} +Na]^+$ 1230.3914, found 1230.3912.

6. Total synthesis of feglymycin (1)

Cbz-D-Hpg-D-Dpg-L-Val-D-Dpg-L-Hpg-D-Dpg-L-Hpg-D-Dpg-L-Val-D-Dpg-L-Hpg-L-Phe-L-Asp(*O*-Bn)-OBn (38)

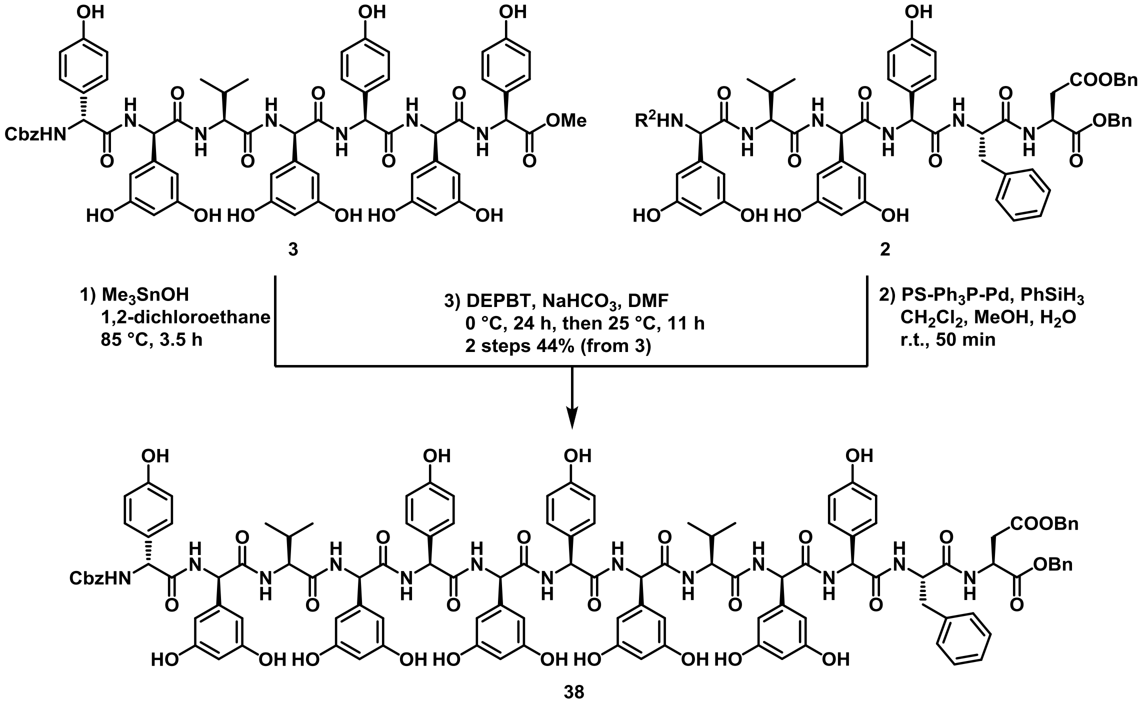

To a solution of heptapeptide **3** (24.3 mg, 0.0201 mmol, 1.0 equiv.) in 1,2-dichloroethane (1.8 mL), trimethyltin hydroxide (75.0 mg, 0.417 mmol, 20.75 equiv.) was added at room temperature under argon. After being stirred at 85 °C for 3.5 h, the reaction mixture was concentrated in vacuo and dissolved in saturated aqueous $NaHCO_3$ (3.0 mL). The aqueous layer was washed with EtOAc (2.5 mL). The organic layer was extracted three times with saturated aqueous $NaHCO_3$ (1.3 mL). The combined aqueous layer was acidified with citric acid (pH 3), extracted five times with EtOAc (2.5 mL). The combined organic layer was washed with 10% citric acid, with water, and twice with brine, dried over Na_2SO_4, filtered and concentrated in vacuo. The residue (23.2 mg) was used for the next reaction without further purification.

A solution of hexapeptide **2** (90.0 mg, 0.0801 mmol, 1.0 equiv.) in CH_2Cl_2 (1.4 mL), MeOH (0.9 mL) and H_2O (0.1 mL) was degassed with argon. Phenylsilane (39 μL, 0.320 mmol, 4.0 equiv.) and tetrakis(triphenylphosphine)palladium, polymer-bound (0.07 mmol/g, 84 mg, 0.00588 mmol, 0.0734 equiv.) were added at room temperature under argon. After being stirred at the same temperature for 50 min, the reaction mixture was filtered and concentrated in vacuo. The residue was reprecipitated from IPA/hexane and used for the next reaction without further purification.

To a solution of crude carboxylic acid **36** (4.1 mg, 0.00343 mmol, 1.0 equiv.) and crude amine **37** (17.6 mg, 0.0169 mmol, 4.93 equiv.) in DMF (206 μL), $NaHCO_3$ (2.7 mg, 0.0321 mmol, 9.36 equiv.) and DEPBT (13.6 mg, 0.0456 mmol, 13.3 equiv.) were added at 0 °C under argon. After being stirred at the same temperature for

24 h, the reaction mixture was warmed to 25 °C. After being stirred at the same temperature for 11 h, three drops of H_2O was added and concentrated in vacuo. The residue was purified by preparative TLC ($MeOH/H_2O/CH_2Cl_2$ 2.25/0.25/7.5 twice, Rf = 0.20) to give tridecapeptide **38** (5.2 mg, 0.00155 mmol, 2 steps 44% from heptapeptide **3**) containing 6.5 equiv. of sodium diethylphosphate or diethylphosphate as a colorless solid. The following spectra were measured after reverse phase column chromatographic purification (InertSep C18 1 g/6 mL, 0% MeOH in H_2O to 83% MeOH in H_2O).

^{1}H NMR (500 MHz, DMSO-d_6): δ 9.65-8.95 (br, 14H), 8.78 (d, *J* = 6.5 Hz, 1H), 8.69 (d, *J* = 8.5 Hz, 1H), 8.67 (d, *J* = 8.5 Hz, 1H), 8.61 (d, *J* = 6.5 Hz, 1H), 8.56-8.46 (m, 5H), 8.40 (d, *J* = 8.0 Hz, 1H), 8.09 (d, *J* = 8.0 Hz, 1H), 7.86 (d, *J* = 8.0 Hz, 1H), 7.81 (d, *J* = 8.0 Hz, 1H), 7.35-7.24 (m, 15H), 7.24-7.11 (m, 7H), 7.06 (d, *J* = 8.5 Hz, 2H), 7.03 (d, *J* = 9.0 Hz, 2H), 6.93 (d, *J* = 8.5 Hz, 2H), 6.66 (d, *J* = 8.5 Hz, 2H), 6.57-6.49 (m, 6H), 6.33 (s, 2H), 6.29 (s, 2H), 6.28 (s, 2H), 6.20 (s, 2H), 6.18 (s, 2H), 6.11 (s, 1H), 6.09 (s, 1H), 6.07 (s, 1H), 6.06-6.02 (m, 2H), 5.63 (d, *J* = 7.5 Hz, 1H), 5.59 (d, *J* = 7.5 Hz, 1H), 5.56 (d, *J* = 7.5 Hz, 1H), 5.52 (d, *J* = 8.0 Hz, 1H), 5.46 (d, *J* = 7.0 Hz, 1H), 5.42-5.35 (m, 2H), 5.31 (d, *J* = 8.0 Hz, 1H), 5.29 (d, *J* = 8.5 Hz, 1H), 5.08-4.95 (m, 6H), 4.69 (ddd, *J* = 6.5, 7.0, 7.0 Hz, 1H), 4.56-4.48 (m, 1H), 4.41-4.29 (m, 2H), 3.01-2.94 (m, 1H), 2.87-2.66 (m, 3H), 1.92-1.81 (m, 2H), 0.60 (d, *J* = 7.0 Hz, 3H), 0.58 (d, *J* = 8.5 Hz, 3H), 0.56 (d, *J* = 7.0 Hz, 3H), 0.53 (d, *J* = 6.5 Hz, 3H); ^{13}C NMR (125 MHz, DMSO-d_6): δ 170.8, 170.4, 170.3, 169.8, 169.7, 169.7, 169.5, 169.5, 169.4, 169.1, 169.0, 158.1, 158.0, 158.0, 158.0, 158.0, 156.8, 156.7, 156.6, 156.5, 156.5, 140.9, 140.8, 140.7, 140.7, 140.6, 137.4, 136.9, 135.8, 135.6, 129.2, 128.6, 128.5, 128.4, 128.4, 128.3, 128.2, 128.1, 128.1, 128.0, 127.8, 127.8, 126.4, 115.0, 114.8, 105.7, 105.6, 105.4, 105.4, 105.3, 101.8, 101.7, 66.4, 66.0, 65.6, 57.9, 57.5, 57.3, 56.8, 56.4, 56.1, 55.8, 55.5, 55.1, 55.0, 53.7, 48.5, 48.5, 35.7, 31.5, 31.5, 19.1, 19.0, 17.5, 17.2; IR (KBr): 3434, 2981, 2933, 2902, 2869, 1642, 1559, 1385, 1227, 1106, 1060, 957, 821 cm^{-1}; $[\alpha]^{26}_D$ = -32.4 (c 0.050, MeOH); HRMS (ESI-TOF): calcd for $[C_{117}H_{115}N_{13}O_{32}+Na]^+$ 2236.7663, found 2236.7642.

Feglymycin (1)

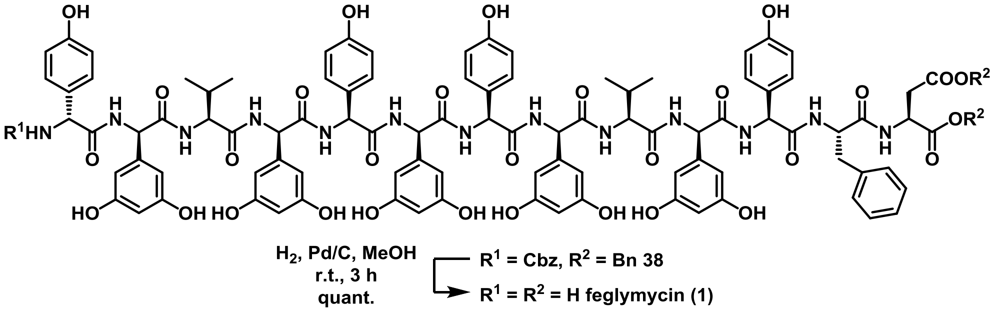

To a solution of tridecapeptide **38** (4.3 mg, 0.00128 mmol, 1.0 equiv.) containing 6.5 equiv. of sodium diethylphosphate or diethylphosphate in MeOH (1.9 mL), 10% Pd/C (2.7 mg, 0.00120 mmol, 0.94 equiv.) was added at room temperature under argon. After being stirred at the same temperature for 1.5 h under H_2, 10% Pd/C (2.7 mg, 0.00120 mmol, 0.94 equiv.) was added at room temperature under argon. After being stirred at the same temperature for 1.5 h under H_2, the reaction mixture was filtered through membrane filter (0.45 μm) and concentrated in vacuo. The residue was purified by reverse phase column chromatography (InertSep C18

1 g/6 mL, 0.05% TFA, 25% MeCN in H_2O to 100% MeCN) and freeze-dried to give feglymycin (**1**) (2.7 mg, quant.) as a colorless solid.

^{1}H NMR (400 MHz, DMSO-d_6): δ 12.75 (br, 1H), 12.44 (br, 1H), 9.76 (s, 1H), 9.33 (s, 1H), 9.31 (s, 1H), 9.29 (s, 1H), 9.24 (s, 2H), 9.17 (s, 2H), 9.15 (s, 2H), 9.10 (s, 4H), 8.93 (d, *J* = 7.5 Hz, 1H), 8.77 (d, *J* = 6.5 Hz, 1H), 8.67 (d, *J* = 7.5 Hz, 1H), 8.65 (d, *J* = 8.5 Hz, 1H), 8.62 (d, *J* = 8.0 Hz, 1H), 8.51 (d, *J* = 6.5 Hz, 2H), 8.44 (br, 3H), 8.34 (d, *J* = 7.5 Hz, 1H), 8.28 (d, *J* = 7.5 Hz, 1H), 8.11 (d, *J* = 8.5 Hz, 1H), 7.94 (d, *J* = 8.5 Hz, 1H), 7.32 (d, *J* = 8.5 Hz, 2H), 7.23 (m, 4H), 7.15 (m, 1H), 7.06 (d, *J* = 8.0 Hz, 2H), 7.05 (d, *J* = 7.5 Hz, 2H), 6.93 (d, *J* = 8.5 Hz, 2H), 6.81 (d, *J* = 8.5 Hz, 2H), 6.73-6.41 (m, 6H), 6.36 (s, 2H), 6.29 (s, 2H), 6.25 (s, 2H), 6.20 (s, 2H), 6.17 (s, 2H), 6.13 (s, 1H), 6.09 (s, 1H), 6.07 (s, 1H), 6.06 (s, 2H), 5.61 (d, *J* = 8.0 Hz, 1H), 5.59 (d, *J* = 8.0 Hz, 1H), 5.53 (d, *J* = 7.5 Hz, 1H), 5.50 (d, *J* = 8.0 Hz, 1H), 5.49 (d, *J* = 7.5 Hz, 1H), 5.46 (d, *J* = 8.0 Hz, 1H), 5.40 (d, *J* = 7.5 Hz, 1H), 5.30 (d, *J* = 7.5 Hz, 1H), 4.99 (br, 1H), 4.53 (ddd, *J* = 9.0, 4.0, 8.5 Hz, 1H), 4.48 (ddd, *J* = 6.5, 7.5, 6.0 Hz, 1H), 4.39-4.30 (m, 2H), 3.04 (dd, *J* = 4.0, 13.5 Hz, 1H), 2.81 (dd, *J* = 9.5, 13.5 Hz, 1H), 2.57-2.53 (m, 1H), 1.92-1.78 (m, 2H), 0.61 (d, *J* = 9.0 Hz, 3H), 0.59 (d, *J* = 6.5 Hz, 3H), 0.55 (d, *J* = 6.5 Hz, 3H), 0.53 (d, *J* = 7.0 Hz, 3H); ^{13}C NMR (100 MHz, DMSO-d_6): δ 172.3, 171.8, 170.4, 170.3, 169.7, 169.7, 169.6, 169.5, 169.1, 169.1, 167.1, 158.4, 158.2, 158.0, 158.0, 156.7, 156.6, 156.6, 141.0, 140.8, 140.7, 140.6, 137.5, 129.5, 129.4, 128.6, 128.6, 128.3, 128.2, 128.1, 128.1, 126.4, 124.3, 115.6, 114.9, 114.8, 105.7, 105.7, 105.5, 105.4, 105.4, 101.9, 101.8, 101.7, 57.2, 56.8, 56.5, 56.1, 55.8, 55.5, 55.2, 55.0, 54.8, 53.9, 48.7, 37.5, 36.3, 31.5, 19.1, 17.6, 17.2; IR (KBr): 3293, 1680, 1640, 1514, 1438, 1390, 1344, 1207, 1142, 1027, 1006, 841, 802, 723 cm^{-1}; $[\alpha]^{26}_D$ = -103.3 (c 0.024, MeOH); mp > 230 °C; HRMS (ESI-TOF): calcd for $[C_{95}H_{97}N_{13}O_{30}+Na]^+$ 1922.6356, found 1922.6358.
